# Light-Mediated Direct Decarboxylative Giese Aroylations without a Photocatalyst

**DOI:** 10.1021/acs.joc.4c02163

**Published:** 2024-10-22

**Authors:** David M. Kitcatt, Eva Pogacar, Le Mi, Simon Nicolle, Ai-Lan Lee

**Affiliations:** † Institute of Chemical Sciences, Heriot-Watt University, Edinburgh EH14 4AS, Scotland, United Kingdom; ‡ GlaxoSmithKline, Gunnels Wood Road, Stevenage SG1 2NY, United Kingdom

## Abstract

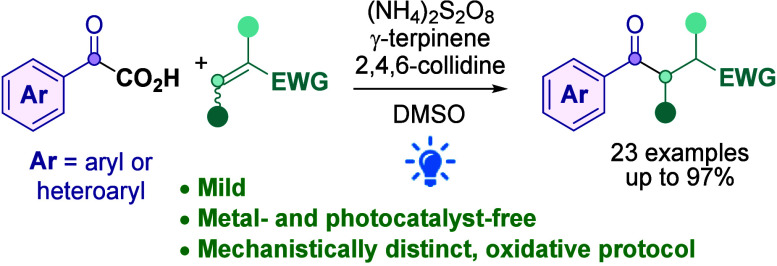

Previous light-mediated approaches to the direct decarboxylative Giese aroylation reaction have mainly relied on the use of a photocatalyst and a reductive quenching pathway. By exploiting a mechanistically distinct oxidative protocol, we have successfully developed a photocatalyst-free, light-mediated direct Giese aroylation methodology.

Recently, α-keto acids **1** have emerged as important acylating reagents due to their availability, stability, low toxicity, and loss of only nontoxic CO_2_ as a byproduct.^1,2^ In recent years, several photocatalytic methods have emerged to enable direct decarboxylative Giese^3,4^ aroylations using **1** to form important 1,4-difunctionalized building blocks^5^**3** (Scheme 1A). These procedures tend to follow a reductive quenching cycle^6^ and require either expensive, toxic, and nonsustainable metals as photocatalysts (Ir/Ru)^7^ or an organocatalyst/enzyme^8^ photocatalyst.^9^ There has so far only been one elegant and seminal example of a photocatalyst-free approach by Gilmour (Scheme 1B).^10^ However, since the mechanism requires formation of an iminium intermediate (from **4**+**5**) as well as an electron donor–acceptor (EDA) complex^11^ involving the aforementioned iminium, the substrate scope is restricted to 3-arylprop-2-enals **4**, rather than more general Michael acceptors **2** (EWG = electron-withdrawing group). In this report, we demonstrate the first mechanistically distinct, oxidative protocol for Giese aroylations and, crucially, show that this enables a photocatalyst-free protocol (Scheme 1C), which exhibits a significantly more general Michael acceptor scope **2** compared to Scheme 1B. Furthermore, the protocol is metal-free and benefits from faster reaction times (5 h vs. overnight–60 h) and greener solvents (DMSO vs. DMF, DCM, DME, 1,4-dioxane, or MeCN)^12^ than the photocatalytic approaches shown in Scheme 1A.

**Scheme 1:**
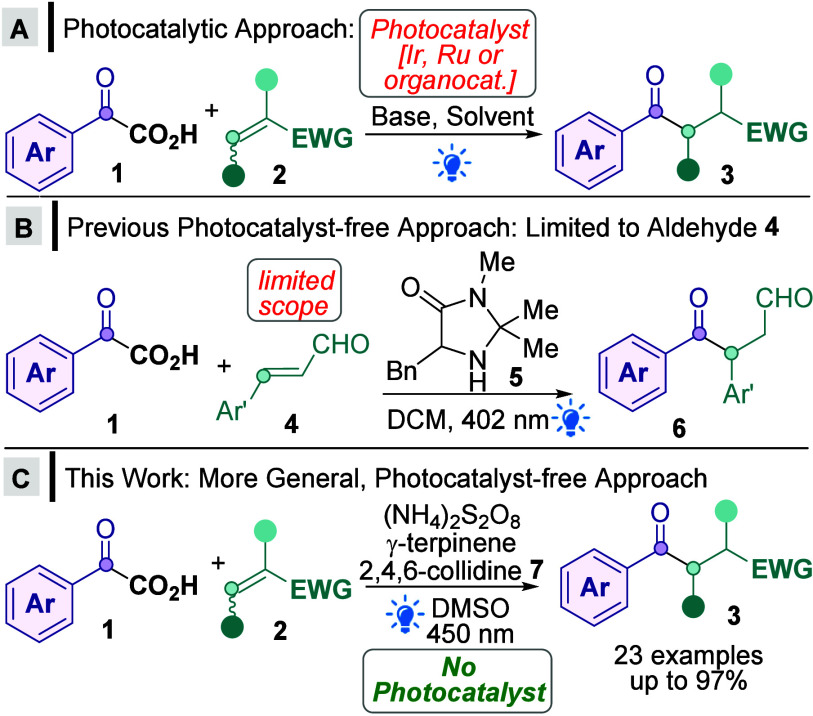
Direct, Decarboxylative Giese Aroylations

The initial impetus for our study was to develop and investigate the first oxidative protocol(s) for the direct decarboxylative Giese aroylation reaction, since our related study on Giese amidations^13^ as well as Baran’s Giese alkylation toward the total synthesis of (−)-maximiscin^14^ demonstrate that methods mediated by chemical oxidants can be very complementary to the established reductive quenching cycle pathway.

We initiated our studies on the model system **1a** + **2a** → **3a** using (NH_4_)_2_S_2_O_8_ as oxidant,^15^ 2,4,6-collidine **7** as base, and γ-terpinene as a hydrogen atom transfer (HAT) source (Table 1; see Supporting Information for full studies). Confusingly, the thermally mediated reaction (50 °C, Conditions A) gave inconsistent yields (57–74%) of **3a** with each repeated attempt (Entry 1). To our surprise, a control reaction in the dark at 50 °C produced a lower yield (Entry 2), indicating that the reaction in Entry 1 is *both* thermally- *and* light-mediated, and the inconsistent yields reflect the different levels of ambient light during each reaction.

**Table 1:**
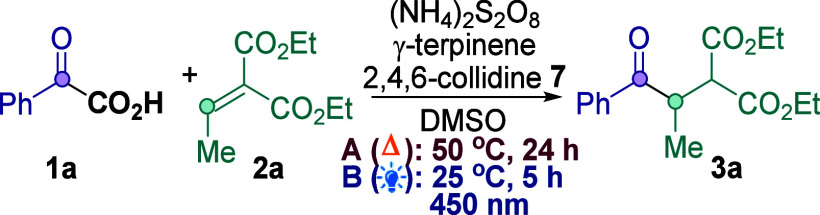

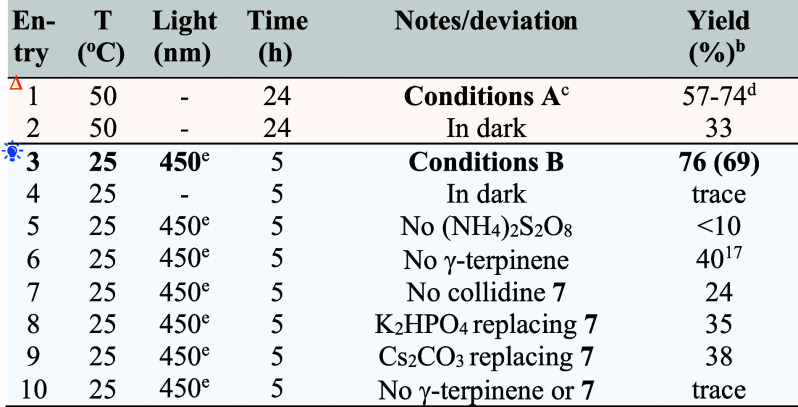
Selected Control Experiments^a^

a **1a** (2 equiv), (NH_4_)_2_S_2_O_8_ (3 equiv), γ-terpinene (2 equiv), **7** (2 equiv).

b Estimated by ^1^H NMR analysis of the crude mixture using CH_2_Br_2_ as the internal standard. Isolated yields in parentheses.

c Ambient light.

d Yields varied over 7 runs.

e Penn PhD M2 Photoreactor.

Since the reaction benefits from ambient light when run at 50 °C, we decided to test a purely light-mediated process to develop a milder, room temperature light-mediated reaction (Conditions B, Entry 3).^13,16^ Crucially, a control reaction in the dark at 25 °C gave only trace amounts of **3a** (entry 4), indicating that the reaction under Condition B is solely light-mediated. Further control reactions show that the persulfate, HAT source γ-terpinene,^17^ and 2,4,6-collidine base **7** are all required for good yields (Entries 5–7, Entry 10). Replacing **7** with inorganic bases results in poorer yields (Entries 8–9). In addition to being more reproducible, the new light-mediated reaction is clearly milder (25 vs. 50 °C) and more efficient (5 h vs. 24 h) than Conditions A.

With optimal conditions in hand, an α-keto acid **1** scope was investigated (Scheme 2), using both Conditions A and B. Replacing the Ph in model substrate **1a** with electron-rich aryls generally provided improved yields of products **3b**–**3d** (88–97%), including the more sterically encumbered mesityl (**3c**, 88%). Results with Me_2_N-substituted aryl (**3e**) exemplify the benefit of milder Conditions B. Conditions A gave a poor yield of **3e** (31%) due to competing aniline demethylation, presumably involving SET^18^ (22% **3e′**), whereas milder Conditions B gave 54% **3e** with no **3e′**. Electron-poor aryls on **1** also reacted poorly under Conditions A but gave good (**3f**, 92%) or moderate (**3g**, 40%) yields under Conditions B. Electron-rich aryls on **1** generally produced better results, reflecting the increased nucleophilicity of the corresponding acyl radical. Pleasingly, thiophene (**3h**, 55%), pyrrole (**3i**–**3j**, 66–79%), and indole (**3k**, 71%) heterocycles were tolerated well, although furan-substituted **1** reacted more sluggishly (**3l**, 35%).

**Scheme 2:**
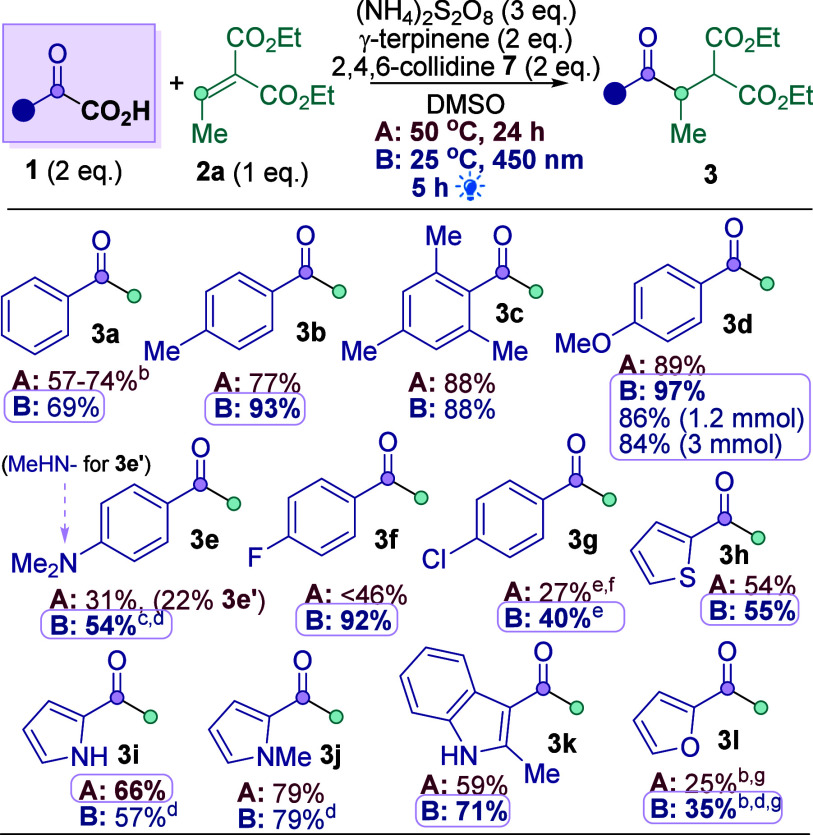
α-Keto Acid Scope Isolated yields reported unless otherwise stated. By ^1^H NMR using CH_2_Br_2_ as internal standard. 77% consumption of **2a**, no **3e**′ observed. 16 h. 3 eq. of **1**, 4 eq. of (NH_4_)_2_S_2_O_8_, 3 eq. of **7**. 75 °C. Approx. 50% conversion.

Light-mediated Conditions B gave better yields than the “thermally”-mediated Conditions A in almost all cases shown in Scheme 2. Since Conditions B are milder (25 vs 50 °C), more selective (see **3e**), more efficient (5 h vs. 24 h), generally higher yielding (Scheme 2) and more reproducible (see Table 1) than Conditions A, the light-mediated reaction is generally the preferred methodology and was thus adopted for the Michael acceptor scope study (Scheme 3). The light-mediated reaction was also readily scaled up to a 3 mmol scale under batch conditions (**3d**, 84%).

**Scheme 3:**
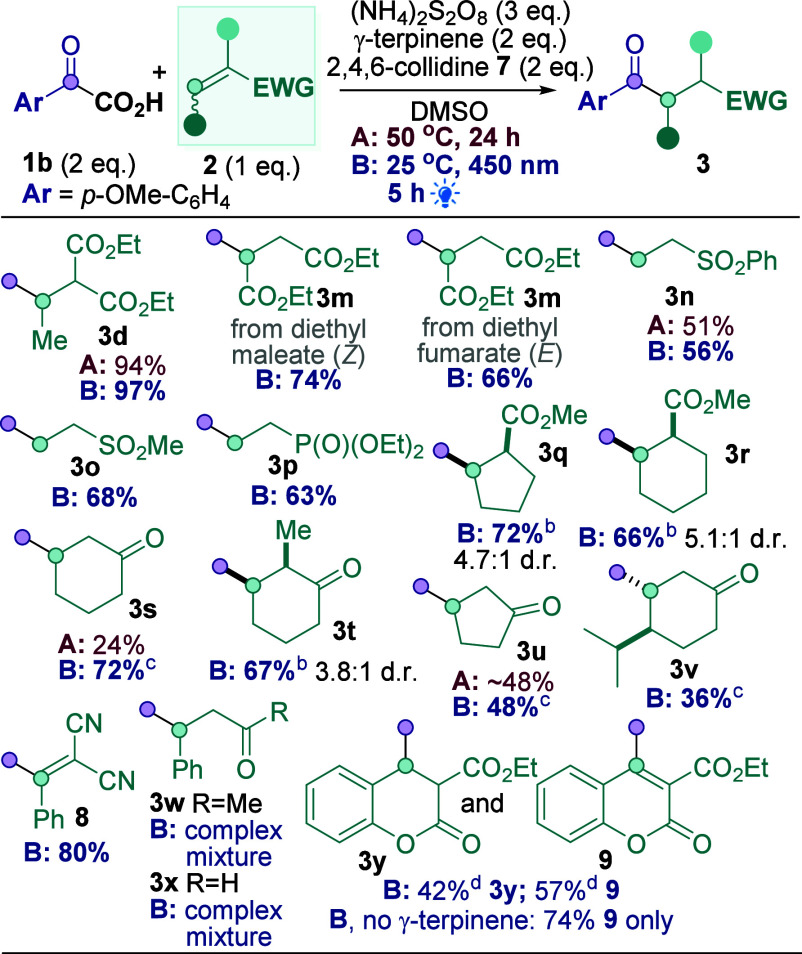
Michael Acceptor Scope lsolated yields unless otherwise stated. 16.5 h. 0.2 M, 24 h. By ^1^H NMR analysis of the crude mixture using CH_2_Br_2_ as the internal standard.

Scheme 3 shows a more general Michael acceptor scope^19^**2** compared to previous photocatalyst-free methodology (Scheme 1B). Comparing Conditions A and B with **3d**, **3n**, **3s**, and **3u** once again confirmed Conditions B to be superior, so only Conditions B were used for the rest of Scheme 3. Diethyl maleate and fumarate both reacted smoothly to give **3m** in 74% and 66%, respectively. Vinyl sulfones (**3n**–**o**, 56–68%) and diethyl vinyl phosphonate (**3p**, 63%) also reacted smoothly. Cyclic Michael acceptors are also suitable substrates (**3q**–**3u**, 48–72%), although the reaction appears to be sensitive to steric hindrance at the γ-position (**3v**, 36%). Surprisingly, reaction with 2-benzylidenemalononitrile gave the β-aroylation product **8** (80%) instead, presumably via a tandem Giese-dehydrogenation reaction. Reaction with ethyl 2-oxo-2*H*-chromene-3-carboxylate produced a mixture of **3y** and **9** under standard conditions, but only tandem Giese-dehydrogenation product **9** (74%) when γ-terpinene is omitted. This reaction implies that the production of **8** and **9** is in competition with HAT of the radical **V** to form **3** in these particular cases^20^ (see later, Scheme 5). A current limitation is that acyclic ketones and aldehydes do not react well (e.g., **3w**–**x**). Nevertheless, since **4** is a suitable substrate in Scheme 1B,^10^ the two methodologies are nicely complementary.

1,4-Difunctionalized compounds such as **3d** can readily undergo further modifications to useful building blocks such as dihydropyridazinone **11** and lactone **12**(21) via the versatile γ-keto carboxylic acid^22^**10** (Scheme 4).

**Scheme 4:**
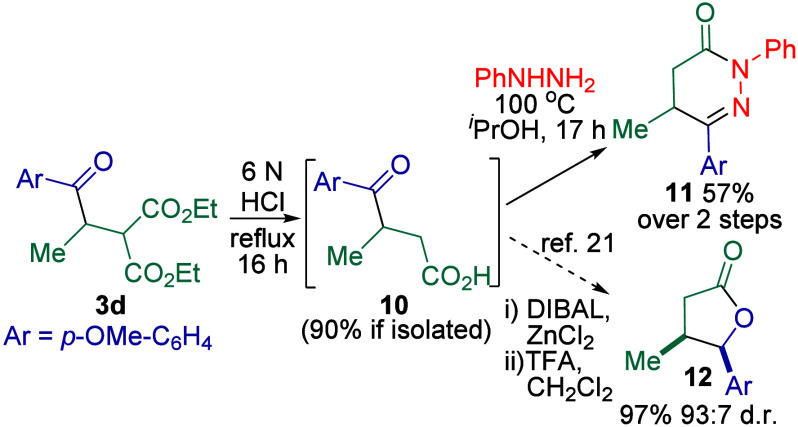
Further Modifications

Our proposed mechanism is shown in Scheme 5. For Conditions A, we have previously shown that the use of DMSO as solvent allows for the breakdown of S_2_O_8_^2–^ to SO_4_^–•^**II**^23^ under mild conditions (40–50 °C).^15c,24,25^ For new light-mediated Conditions B, key control reactions show that the reaction requires light, persulfate, and collidine to proceed well (Table 1). Nevertheless, it is known that the photodecomposition of S_2_O_8_^2–^ on its own is slow and requires 280 nm (UV light)^26^ and that the reaction is not thermally mediated at 25 °C (Entry 4, Table 1). The breakdown of S_2_O_8_^2–^ to **II** must therefore be light-mediated but assisted by other species absorbing at around 450 nm.

**Scheme 5:**
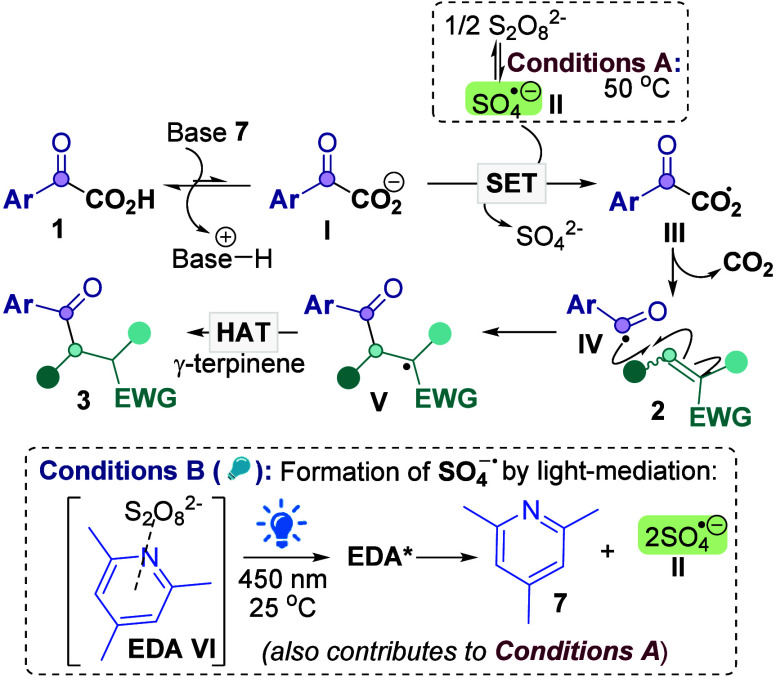
Proposed Mechanism

UV–vis studies show that **7**, persulfate, and **1a** do not absorb at 450 nm on their own (Figure 1). The combinations of **1a**+**7** and **7**+(NH_4_)_2_S_2_O_8_, however, do absorb at ≈450 nm and show color change and bathochromic shift. The first possibility is that the contact ion pair (CIP)^11b^ species formed between **1a**+**7** absorbs light to help photodecompose S_2_O_8_^2–^ to **II**; however, the lack of quenching of a **1**+**7** mixture by persulfate does not support this theory. Direct formation^27^ of **IV** from excited I* was also ruled out because it is inconsistent with our observation that persulfate is necessary (Entry 5, Table 1). Instead, we propose that EDA formation between **7**+(NH_4_)_2_S_2_O_8_ allows for absorption of visible light at ≈450 nm and causes photodecomposition of S_2_O_8_^2–^ to SO_4_^–•^**II**^23^ (Scheme 5).^15d,28,29^ Job’s plot analyses^30^ display maximum absorption at 4:1 S_2_O_8_^2–^:**7**, suggesting that the mechanism does not proceed via a SET from donor to acceptor of the EDA.^31^ Upon formation of **II**, SET between **II** and glyoxylate **I** can subsequently occur to give radical **III**,^32^ which can decarboxylate to **IV**,^33^ for the Giese addition with **2**. Radical **V** can then undergo HAT to yield product **3**.^34^

**Figure 1:**
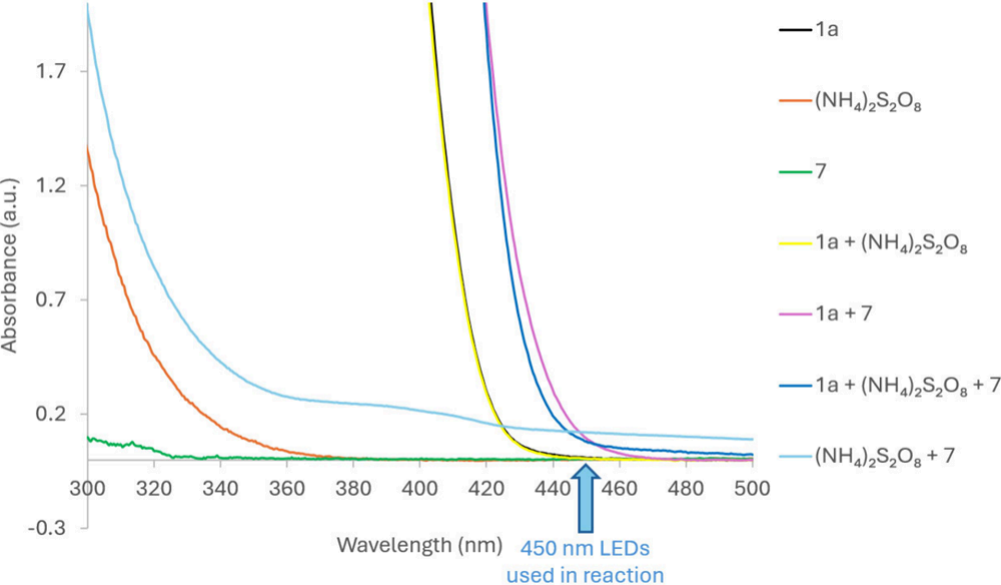
Selected UV–vis absorption studies.

In conclusion, we have demonstrated the first direct decarboxylative Giese aroylations to occur via a mechanistically distinct oxidative protocol. Crucially, the oxidative protocol allows for an efficient *photocatalyst-free* light mediated methodology. The EDA formed between **7**+(NH_4_)_2_S_2_O_8_ is likely the key light-absorbing species, and further investigations are currently underway to exploit combinations of appropriate bases with persulfate in other photocatalyst-free reactions.

## Data Availability

The data underlying this study are available in the published article and in its Supporting Information and openly available at 10.17861/f52bb21c-9082-4aba-9b24-3f6410e1030d.

## Supporting Information Available

Optimization studies, all experimental details, and characterization and copies of NMR data (PDF)
