# Supplementary material for: Light-Mediated Direct Decarboxylative Giese Aroylations without a Photocatalyst
Source: J Org Chem. 2024 Oct 22;89(21):16055–9. doi: 10.1021/acs.joc.4c02163 (PMC11536358; doi:10.1021/acs.joc.4c02163)

Supplementary Information

Light-Mediated Direct Decarboxylative Giese Aroylations without a Photocatalyst

David M. Kitcatt,[a] Eva Pogacar,[a] Le Mi,[a] Simon Nicolle,[b] and Ai-Lan Lee[a,*]

[a]Institute of Chemical Sciences, Heriot-Watt University, Edinburgh EH14 4AS, Scotland, U. K.

[b]GlaxoSmithKline, Gunnels Wood Rd, Stevenage SG1 2NY, U. K.

*Ailan.Lee@ed.ac.uk

Table of Contents

1. General Experimental S3
2. Optimisation Studies S4
3. UV-vis Absorption Studies S6
4. Stoichiometry of the EDA complexes in solution S8
5. Stern-Volmer Quenching Studies S9
7. Quantum Yield Determination S10
8. Starting Material Synthesis S14
9. Product Characterisation S16
10. References S55
11. Appendix S56

1. General Experimental

All reagents and solvents were purchased from commercially available sources and used without any further purification. ^{1}H and ^{13}C Nuclear Magnetic Resonances were recorded on Bruker® AV300 or AV400 (^{1}H NMR at 400 MHz, $^{13}C\{^{1}H\}$ NMR at 101 MHz, ^{19}F NMR at 376 MHz, and ^{31}P NMR at 162 MHz) spectrometers with chemical shifts (δ) given in parts per million (ppm), employing chloroform-*d* or acetone-d_6 as solvents with their respective residual solvent signals[1] reported as their standard reference peaks. Multiplicities are indicated as br (broad), s (singlet), d (doublet), t (triplet), q (quartet), quin (quintet), m (multiplet) or a combination of these. Coupling constants (*J*) are given in Hertz (Hz). Yields calculated by ^{1}H NMR analysis were determined using dibromomethane (2H, 4.93 ppm) as internal standard which were added after work-up. Note that for $^{13}C\{^{1}H\}$ NMR charactersisation, only signals that could not be differentiated by 1 d.p. were quoted to 2 d.p. High resolution mass spectrometric (HRMS) data were reported with ion mass/charge (m/z) ratios as values in atomic mass units. High-Resolution Mass Spectra were recorded under ESI conditions by the analytical services at the University of Edinburgh. Infrared spectra were obtained on Perkin-Elmer Spectrum 100 FT-IR Universal ATR Sampling Accessory, deposited neat to a diamond/ZnSe plate. Column chromatography was carried out using Matrix silica gel 60 from Fluorochem. TLC was performed using Merck silica gel 60 F254 and visualised by UV (254 nm) and/or stained using aqueous potassium permanganate solution. Unless otherwise stated, all reactions requiring heating were carried out using DrySyn® heating blocks on a stirrer-hotplate (see Figure S7). Unless otherwise stated, all reactions requiring irradiation were carried out using Penn PhD Photoreactor M2 (fan speed = 6800 rpm, light intensity = 100%, wavelength = 450 nm, temperature = 25 °C, stirring = 800 rpm) (see Figure S8).

2. Optimisation Studies

"Thermal":

Table S1. Optimisation studies for Conditions A.

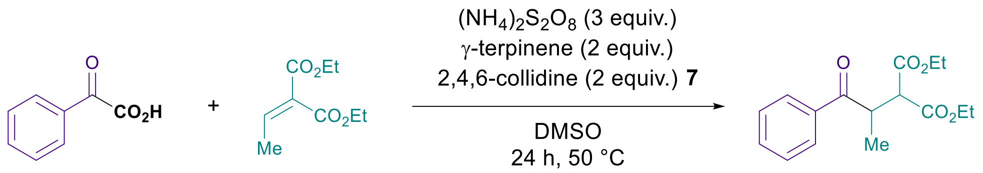

1a (2 equiv.) **2a** (0.16 mmol, 1 equiv.) **3a**

Entry	Deviations from standard conditions	% of 2a remaining[a]	Yield of 3a (%)[a]
1.	None[b]	5-16 (average = 10%)	57-74 (average = 63%)
2.	1 equiv. of **1a**	28	43
3.	3 equiv. of **1a**	14	61
4.	2 equiv. of $(NH_4)_2S_2O_8$	21	56
5.	4 equiv. of $(NH_4)_2S_2O_8$	31	48
6.	3 equiv. of γ-terpinene	28	53
7.	0.12 mmol of **2a**[c]	6	66
8.	1 equiv. of **7**[c]	10	57
9.	0.5 M[d]	6	61
10.	0.3 M[c]	trace	63
11.	0.15 M[c]	trace	53
12.	In dark	44	33

Reaction concentration is 0.4 M of **2a** in DMSO unless otherwise stated. [a]Yields were determine by ^1H NMR of the crude reaction mixture after aq. workup, using CH_2Br_2 as internal standard. n.d. = not detected. [b]Yields varied over 7 runs. [c]0.12 mmol of **2a**. [d]0.20 mmol of **2a**.

With Light:

Table S2. Optimisation studies for Conditions B.

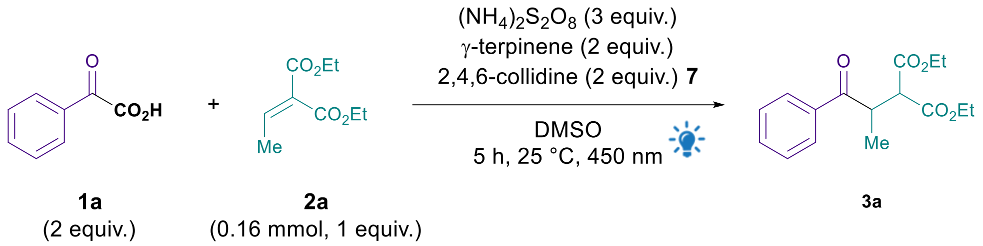

Entry	Deviations from standard conditions	% of 2a remaining[a]	Yield of 3a (%)[a]
1.	365 nm, 17 h	<5	64
2.	420 nm, 17 h	<5	76
3.	450 nm, 17 h	<5	77
4.	None	10	76[b] (69% isolated)
5.	0.32 mmol of **2a**	14	70
6.	In dark	91	trace
7.	No $(NH_4)_2S_2O_8$	96	<10
8.	No γ-terpinene	10	40
9.	No γ-terpinene, 17 h	trace	35
10.	1 equiv. of terpinene	29	55
11.	3 equiv. of terpinene	28	61
12.	No **7**	71	24
13.	No **7**, 17 h	30	49
14.	K_2HPO_4 instead of **7**	62	35
15.	Cs_2CO_3 instead of **7**	9	38
16.	No γ-terpinene and no **7**	53	trace
17.	With TEMPO (2 equiv.)	88	n.d.
18.	CH_2Cl_2 instead of DMSO	87	9
19.	Acetone instead of DMSO	86	13
20.	MeCN instead of DMSO	98	10
21.	MeOH instead of DMSO	86	n.d.

Reaction concentration is 0.4 M of **2a** in DMSO unless otherwise stated. [a]Yields were determine by ^1H NMR of the crude reaction mixture after aq. workup, using CH_2Br_2 as internal standard. [b]Reproducible over multiple runs. n.d. = not detected by ^1H NMR analysis.

3. UV-vis Absorption Studies

UV-vis absorption spectra of individual reactants and/or mixtures of reactants in DMSO were recorded on a Shimadzu UV-2550 spectrophotometer. The following concentrations were used for the studies, mirroring their concentrations in the reactions (Figures S1 and S2): 2-oxo-2-phenylacetic acid **1a** (0.4 M), $(NH_4)_2S_2O_8$ (0.6 M), γ-terpinene (0.4 M), and 2,4,6-collidine **7** (0.4 M). Baseline correction was carried out to ensure that the baseline was at zero for all spectra. The components that are consistently present in each different Giese aroylation reaction are an aryl α-keto ester **1**, ammonium persulfate, 2,4,6-collidine **7**, γ-terpinene and DMSO. Therefore, these were the components that were investigated in our UV-vis studies. The individual components of the reaction mixture are all colourless (**1a**, **7**, $(NH_4)_2S_2O_8$, γ-terpinene) and do not show any significant absorption in the UV-vis absorption spectra at the reaction irradiation wavelength of approx. 450 nm. Mixtures of these species were then observed for any potential colour change, and UV-vis absorption spectra were generally only recorded for mixtures that showed a colour change (with a few others for comparison purposes).

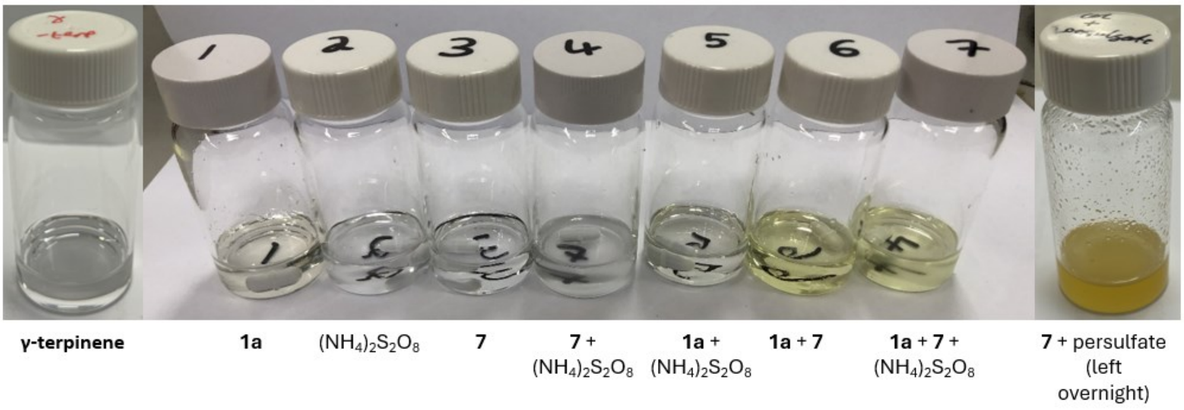

Figure S1. Solutions of **1a**, γ-terpinene, $(NH_4)_2S_2O_8$, **7**, and some of their mixtures in DMSO.

7 + persulfate takes approximately 30 min to dissolve and turns yellow over time, for example, see Figure S1. Only the mixtures of **1a** + **7**, **7** + persulfate, and **1a** + **7** + $(NH_4)_2S_2O_8$ showed a significant colour change.

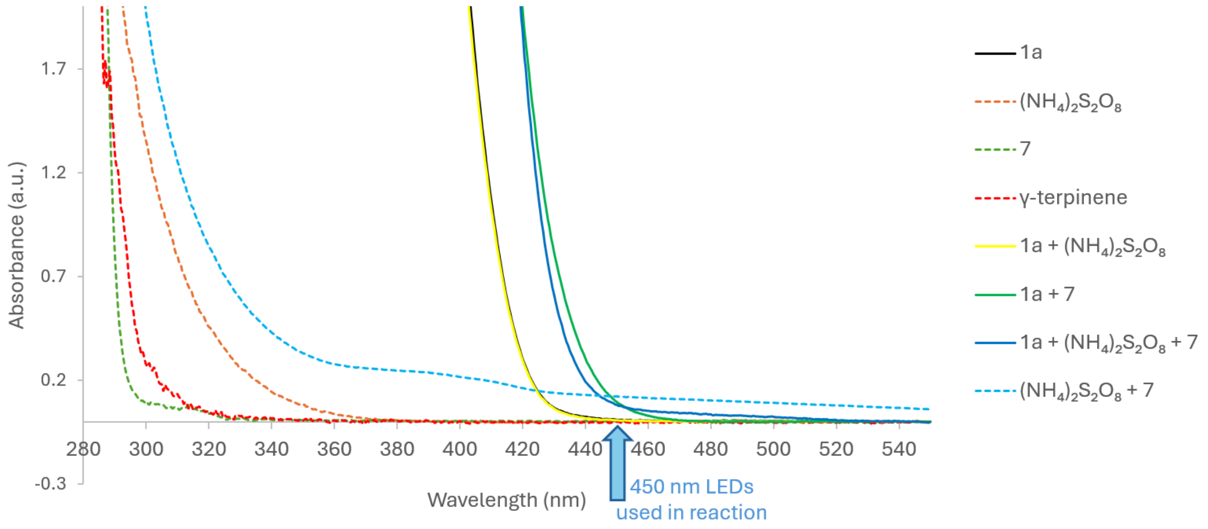

Figure S2. UV-vis absorption studies of solutions of **1a**, γ-terpinene, $(NH_4)_2S_2O_8$, **7**, and some their mixtures in DMSO.

4. Stoichiometry of the EDA complexes in solution

The Job's method of continuous variation was performed to establish the molar donor/acceptor ratio of the EDA complexes in solution.[2] A Job's plot was constructed in order to evaluate the stoichiometry of the EDA complexes between **7** with $(NH_4)_2S_2O_8$. The absorption at 450 nm of DMSO solutions with different donor/acceptor ratios but constant concentrations of the two components was measured. Two standard solutions were prepared. Solution A: 2,4,6-collidine **7** in DMSO (c = X M); and solution B: persulfate in DMSO (c = X M). Using solutions A and B, 6 samples were prepared with differing ratios of **7**:persulfate and the absorbance of these 6 samples at 450 nm was recorded. The absorbance values were plotted against the molar fraction (%) of persulfate. The maximum absorbance obtained was 4:1, **7**:persulfate indicating the stoichiometries of the EDA complexes in solution (Figures S3).

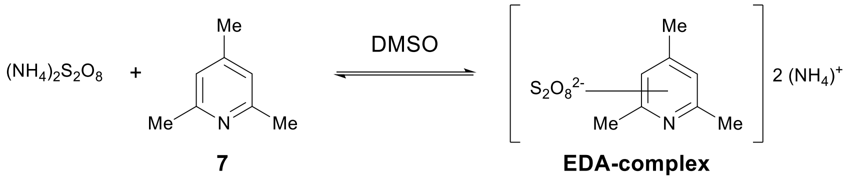

[persulfate] + [**7**] = 0.4 M

[persulfate] = from 0 to 0.4 M

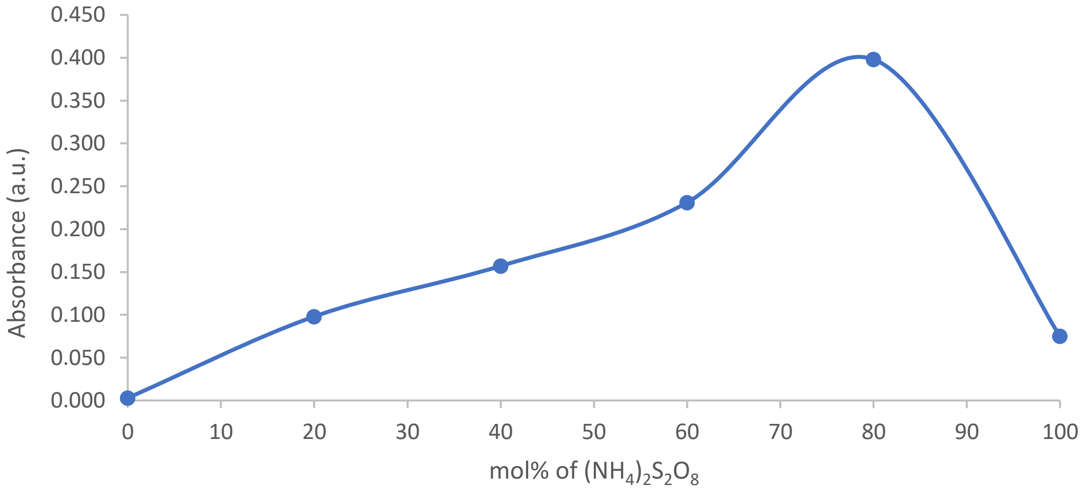

Figure S3: Job's plot for **7** with persulfate.

5. Stern-Volmer Quenching Studies

Stock solutions A (**1a** (0.020 mM) with **7** (0.020 mM) in DMSO) and B (**1a** (0.020 mM) with **7** (0.020 mM) and $(NH_4)_2S_2O_8$ (0.060 mM) in DMSO) were prepared. Solutions of different aliquot ratios of A and B were prepared *in situ* to change the concentration of $(NH_4)_2S_2O_8$ (0.004 mM, 0.008 Mm, 0.010 mM. 0.012 mM, 0.016 mM, 0.020 mM, 0.030 mM, 0.050 mM. and 0.060 mM in turn) whilst maintaining constant concentrations of **1a** and **7**. The fluorescence emission spectra for the solutions were measured using a Perkin Elmer LS 55 fluorescence spectrometer in a 10 x 10 mm fluorescence quartz cuvette, equipped with a PTFE lid. The solutions were irradiated at 450 nm approximately and the emission intensity was recorded from 400 nm to 600 nm. The Stern-Volmer was plotted from the fluorescence emission spectra (Figure S4). The addition of persulfate to **1a**+**7** does not quench the fluorescence, and is thus inconsistent with the initial theory that the contact ion pair species formed between **1a**+**7** absorbs light to help with the photodecomposition of persulfate to **II**.

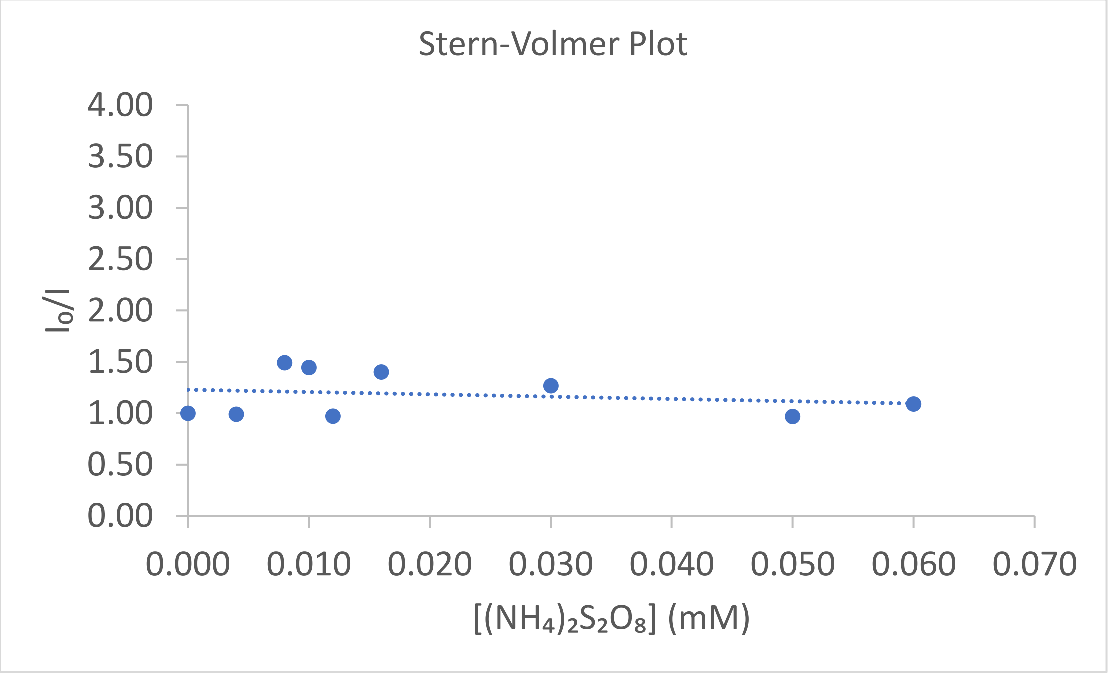

Figure S4. (A) Fluorescent emission spectrum of **1a** and **7** at different concentration of $(NH_4)_2S_2O_8$ and (B) Stern-Volmer plot.

7. Quantum Yield Determination

General Information:

The following procedure was adapted from the literature.[3] Samples were irradiated using Penn PhD M2 Photoreactor M2 at 450 nm with 50% light intensity and fan speed = 6800 rpm.

Photon flux measurements:

a. Potassium ferrioxalate trihydrate

To a warm, stirred aqueous solution of potassium oxalate monohydrate (12 g, 65.1 mmol, 3.3 eq.) in DI water (20 mL) at 70 °C was added an aqueous solution of iron (III) chloride (3.2 g, 19.7 mmol, 1.0 eq.) in DI water (8 mL). The reaction was then cooled to rt and further cooled to 0 °C to precipitate a light green solid. This solid was then recrystallised three more times with water and left to air-dry overnight. ***Caution:*** *potassium ferrioxalate trihydrate is sensitive to light and should be kept in the dark as much as possible.*

b. 1,10-Phenanthroline buffer

An aqueous solution in DI water of sodium acetate (4.92 g, 60.0 mmol), 1,10-phenanthroline (100 mg, 0.555 mmol) and concentrated sulfuric acid (1 mL) was prepared using a 100 mL volumetric flask.

c. Determination of photon flux

A 0.15 M aqueous solution of potassium ferrioxalate trihydrate (1.47 g, 3.00 mmol) in DI water (20 mL) was prepared using a 20 mL volumetric flask. 1.0 mL of the prepared solution was transferred to a 2 mL vial and irradiated for 20 seconds. The vial was then returned to darkness. 0.5 mL of the irradiated solution was transferred to a 25 mL volumetric flask, and 5 mL of the phenanthroline buffer was added and diluted to the mark with DI water. A stirrer bar was added and the solution was stirred for 20 minutes at room temperature. 250 µL was transferred to a quartz cuvette along with DI water (2.5 mL). A UV-Vis spectrum was then obtained from 650-200 nm using the “slow” scan rate. The absorbance at 510 nm was then used to determine the

amount of Fe(II) which formed during irradiation and thereby the photon flux of the photoreactor.

d. Calculating photon flux

Step 1: Concentration of the Fe(II) in the cuvette

The photolysis gives a ligated Fe^{2+} complex that displays a characteristic absorbance peak at 510 nm. (ε = 11110 $L^{-1}cm^{-1}mol^{-1}$). The concentration of Fe^{2+} in the cuvette can be calculated using the Beer-Lambert Law:

$$A = \varepsilon l C$$

Where ε is molar absorptivity, l is pass length, and C is concentration.

Step 2: Concentration of Fe(II) upon irradiation

From the cuvette concentration calculated above, the concentration of Fe(II) in the vial after photolysis can be found using the dilution equation (two times):

$$C_1V_1 = C_2V_2$$

Step 3: Photon flux

The moles of incident photons can be approximated using the absolute quantum yield of Fe(II), previously found to be $\Phi_{Fe(II),\ 457.9\ nm} = 0.85$.[4] Dividing the moles of photons by the time irradiated then gives the photon flux in the units photons per second. Two independent trials of irradiation times 5 s, 10 s and 20 s gave an average photon flux of 1.39 x 10^{-6} mol s^{-1} (std. dev. = 0.31 x 10^{-6}).

Table S3. Determination of photon flux.

Time (s)	Absorbance at 510 nm	Photon flux (x10^{-6} mol s^{-1})
5	0.145	1.69
10	0.260	1.51
20	0.329	0.96

Example photon flux calculation (for 10 s):

Step 1: Concentration of the Fe(II) in the cuvette

$$C_{cuvette} = \frac{A}{\varepsilon l} = \frac{0.260}{(11110\ L^{-1}cm^{-1}mol^{-1})\ \times\ (1 \times 10^{-2}\ cm)} = 2.34 \times 10^{-5}\ M$$

Step 2: Concentration of Fe(II) upon irradiation

$$C_{vol\ flask} = \frac{C_{cuvette}V_{cuvette}}{V_{from\ vol\ flask}} = \frac{(2.34 \times 10^{-5}M) \times (2.75 \times 10^{-3}L)}{0.25 \times 10^{-3}\ L} = 2.57 \times 10^{-4}\ M$$

$$C_{sample} = \frac{C_{vol\ flask}V_{vol\ flask}}{V_{sample}} = \frac{(2.57 \times 10^{-4}M) \times (25 \times 10^{-3}L)}{0.50 \times 10^{-3}\ L} = 1.29 \times 10^{-2}\ M$$

Step 3: Photon flux

$$mol\ photons = \frac{C_{sample}V_{reaction}}{\Phi_{Fe(II),457.9\ nm}} = \frac{(1.29 \times 10^{-2}M) \times (1.0 \times 10^{-3}L)}{0.85} = 1.51 \times 10^{-5}\ mol$$

$$photon\ flux = \frac{mol\ photons}{t_{irradiation}} = \frac{1.51 \times 10^{-5}\ mol}{10\ s} = 1.51 \times 10^{-6}\ mol\ s^{-1}$$

e. *Determining quantum yield*

The quantum yield (Φ) was determine both for Conditions A and C. The quantum yield of a reaction can be obtained by stopping the reaction at varying degrees of conversion using the following relationship:

$$\Phi = \frac{moles\ of\ product}{moles\ of\ incident\ photons} = \frac{moles\ of\ product}{photon\ flux \times reaction\ time}$$

Quantum yield for the Giese amidation under standard conditions:

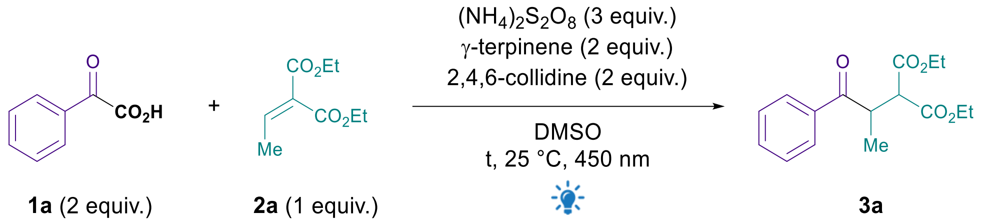

The above model reaction (0.16 mmol scale) was used to determine the quantum yield of the reaction under standard conditions. NMR yields were determined for reactions conducted over 1.0, 2.5, 3.5, and 5.0 hours, giving an average Φ of 6.97 x 10^{-3} (std. dev. = 1.48 x 10^{-3}).

Table S4. Determination of average quantum yield for the Giese aroylation under standard conditions.

t (h)	NMR yield of 9p (%)[a]	Quantum yield Φ (x10^{-3})
1.0	28	8.89
2.5	58	7.44
3.5	72	6.60
5.0	76	4.88

Reaction concentration is 0.4 M of **2a** in DMSO. [a]Yields were determine by ^{1}H NMR using CH_2Br_2 as internal standard.

8. Starting Material Synthesis

α-Keto acids (Figure S5) were either purchased or synthesised from their corresponding ethyl esters using literature procedure.[5]

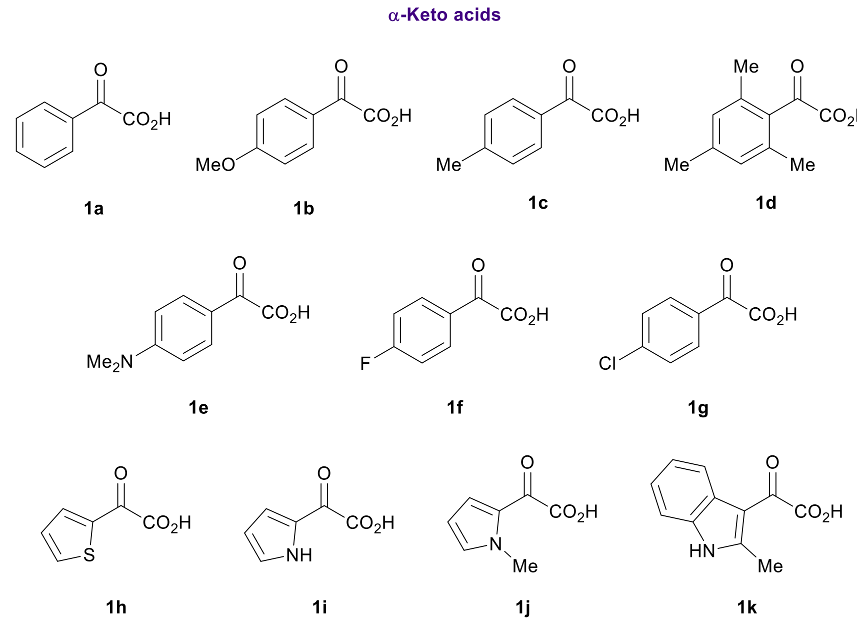

Figure S5. α-Keto acids used in this study.

Attempts to apply Conditions A (at 75 °C) and B with pyruvic acid **1l** as an aliphatic α-keto acid suffered from low yields of **3w** (up to 25%) and low consumption of **2a** (up to 50%) (see Scheme S1).

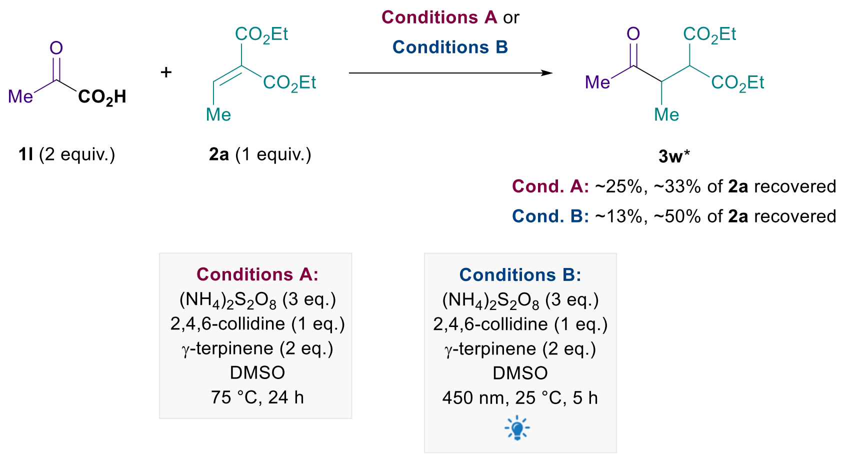

Scheme S1. Using pyruvic acid as **1**. *Yields were determined by ^{1}H NMR using CH_2Br_2 as internal standard.

All Michael acceptors (Figure S6) were purchased from Sigma Aldrich and Fluorochem and used as purchased, apart from **2l** which was synthesised according to literature procedure.[6]

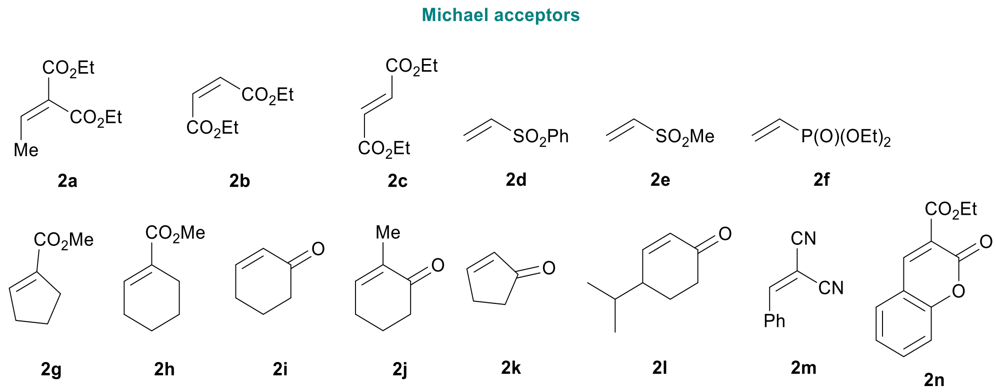

Figure S6. Michael acceptors used in this study.

9. Product Characterisation

General Procedures for Giese Amidation Reactions

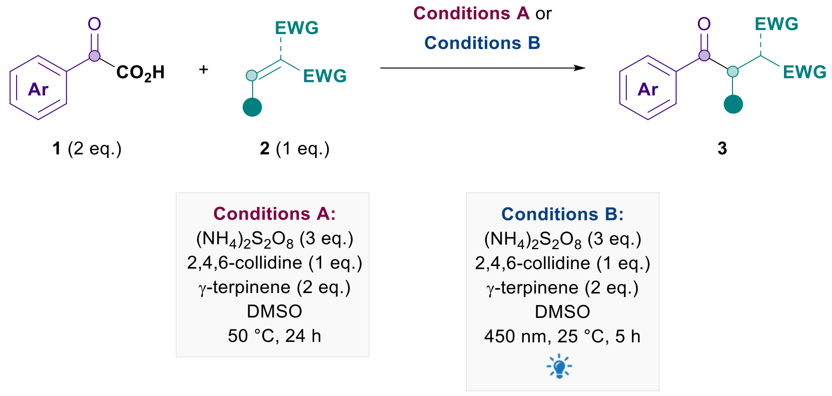

General Procedure for Conditions A: To a 4 mL vial equipped with a magnetic stirrer bar was added Michael acceptor **2** (1 eq.), oxamic acid **1** (2 eq.), $(NH_4)_2S_2O_8$ (3 eq.), γ-terpinene (2 eq.), and 2,4,6-collidine **7** (1 eq.). Separately, a Schlenk tube containing DMSO was sparged with argon (balloon) for 15-20 min. DMSO [0.4 M of **2** in DMSO] was then transferred to the 4 mL vial (choose vial size to have >50% headspace), and the resulting solution was sparged with argon for 5 min. The vial was quickly sealed tight and stirred at 50 °C for 24 h with continuous stirring. The reaction mixture was then diluted with CH_2Cl_2 (15 mL) and washed with sat. $NaHCO_3$ solution (50 mL) and the aqueous phase was then extracted with CH_2Cl_2 (3 x 10 mL). The combined organic layers were then washed with aq. 1 M HCl (60 mL), and the aq. layer was extracted with more CH_2Cl_2 (20 mL). The combined organic layers were then dried over $MgSO_4$ and concentrated *in vacuo* to give the crude product.

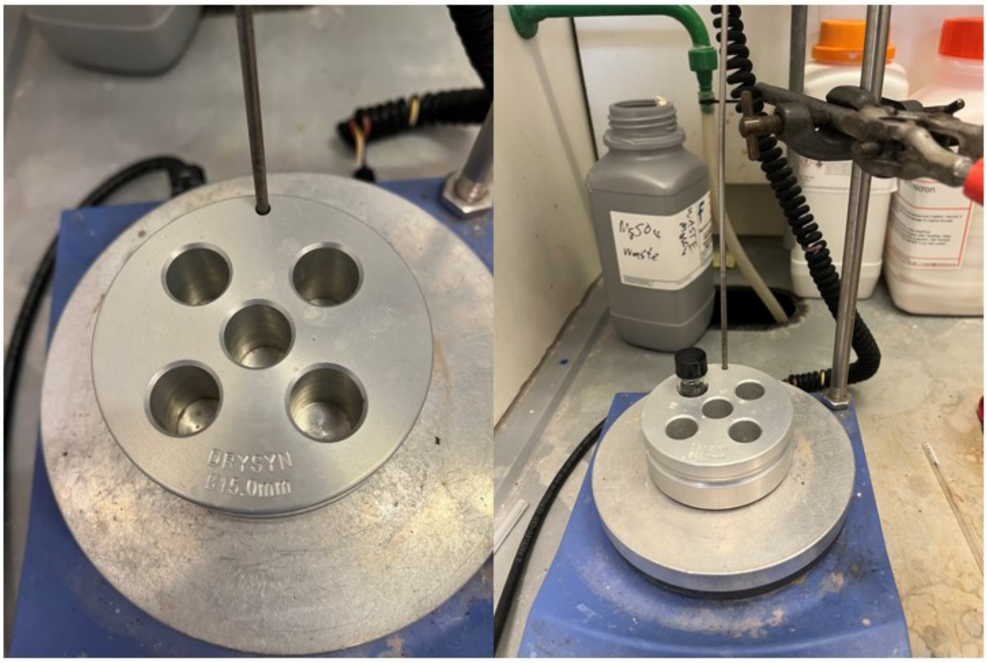

Figure S7. Typical reaction set-up for Conditions A. Left: DrySyn® block used for reactions. Right: DrySyn® block with 4 mL vial inserted.

General Procedure for Conditions B: To an oven-dried 4 mL vial equipped with a magnetic stirrer bar was added Michael acceptor **2** (1 eq.), oxamic acid **1** (2 eq.), $(NH_4)_2S_2O_8$ (3 eq.), γ-terpinene (2 eq.), and 2,4,6-collidine **7** (1 eq.). Separately, a Schlenk tube containing DMSO was sparged with argon (balloon) for 15-20 min. DMSO [0.4 M of **2** in DMSO] was then transferred to the 4 mL vial (choose vial size to have >50% headspace) and the resulting solution was sparged with argon for 5 min. The vial was quickly sealed tight and irradiated with 450 nm blue LEDs using the Penn PhD Photoreactor M2 for 5 h with continuous stirring. The reaction mixture was then diluted with CH_2Cl_2 (15 mL) and washed with sat. $NaHCO_3$ solution (50 mL) and the aqueous phase was then extracted with CH_2Cl_2 (3 x 10 mL). The combined organic layers were then washed with aq. 1 M HCl (60 mL), and the aq. layer was extracted with more CH_2Cl_2 (20 mL). The combined organic layers were then dried over $MgSO_4$ and concentrated *in vacuo* to give the crude product.

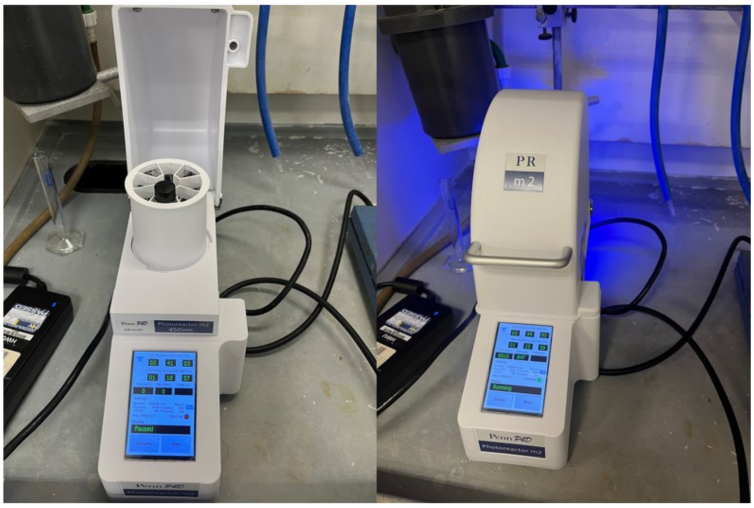

Figure S8. Typical reaction set-up for Conditions B. Left: Penn PhD Photoreactor M2 with 4 ml vial inserted. Right: Penn PhD Photoreactor M2 with running blue LEDs.

Diethyl 2-(1-oxo-1-phenylpropan-2-yl)malonate (3a)

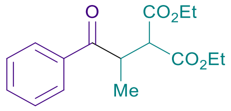

Using Conditions A: General Procedure for Conditions A was followed. Diethyl 2-ethylidenemalonate **2a** (29.6 mg, 0.16 mmol, 1.0 eq.), 2-oxo-2-phenylacetic acid **1a** (48.2 mg, 0.32 mmol, 2.0 eq.), $(NH_4)_2S_2O_8$ (109.6 mg, 0.48 mmol, 3.0 eq.), γ-terpinene (51.3 µL, 0.32 mmol, 2.0 eq.), and 2,4,6-collidine **7** (42.3 µL, 0.32 mmol, 2.0 eq.) in DMSO (0.4 mL) was heated to 50 °C for 24 h. The yield of diethyl 2-(1-oxo-1-phenylpropan-2-yl)malonate **3a** was determined by ^1H NMR analysis of the crude product using CH_2Br_2 as the internal standard. (57-74% over 7 runs).

Using Conditions B: General Procedure for Conditions B was followed. Diethyl 2-ethylidenemalonate **2a** (29.6 mg, 0.16 mmol, 1.0 eq.), 2-oxo-2-phenylacetic acid **1a** (48.1 mg, 0.32 mmol, 2.0 eq.), $(NH_4)_2S_2O_8$ (109.8 mg, 0.48 mmol, 3.0 eq.), γ-terpinene (51.3 µL, 0.32 mmol, 2.0 eq.), and 2,4,6-collidine **7** (42.3 µL, 0.32 mmol, 2.0 eq.) in DMSO (0.4 mL) was irradiated with 450 nm for 5 h. The crude was then purified *via* column flash chromatography eluting with 93:7 hexane:EtOAc, followed by a second purification *via* flash column chromatography eluting with 95:5 hexane:EtOAc. The solvent was then removed *in vacuo* to give diethyl 2-(1-oxo-1-phenylpropan-2-yl)malonate **3a** (32.2 mg, 0.11 mmol, 69%).

Characterisations:

Appearance: Colourless oil. **^{1}H NMR (400 MHz, Chloroform-*d*):** δ ppm 8.00 (d, J = 8.0 Hz, 2H, ArH), 7.57 (t, J = 7.0 Hz, 1H, ArH), 7.48 (t, J = 7.5 Hz, 2H, ArH), 4.31 – 4.03 (m, 5H, C<u>H</u>Me, 2 x OCH_2), 3.98 (d, J = 11.0 Hz, 1H, C<u>H</u>$(CO_2Et)_2$), 1.31 (t, J = 7.0 Hz, 3H, $CH_2C\underline{H}_3$), 1.23 – 1.12 (m, 6H, $CHC\underline{H}_3$, $CH_2C\underline{H}_3$). **^{13}C{^{1}H} NMR (101 MHz, Chloroform-*d*):** δ ppm 201.7 (C), 168.9 (C), 168.5 (C), 135.7 (C), 133.4 (CH), 128.8 (CH), 128.7 (CH), 61.80 (CH_2), 61.77 (CH_2), 55.1 (CH), 40.6 (CH), 16.0 (CH_3), 14.3 (CH_3), 14.0 (CH_3). **IR:** ν_{max}/cm^{-1} 2981, 2937, 2904, 2878, 1746, 1729, 1682, 1597, 1581, 1448, 1368, 1291, 1235, 1182. **TLC:** R_f = 0.22 (90:10 hexane:EtOAc). Data is consistent with the literature.[7]

Diethyl 2-(1-oxo-1-(*p*-tolyl)propan-2-yl)malonate (3b)

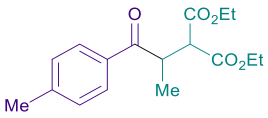

Using Conditions A: General Procedure for Conditions A was followed. General procedure for Conditions A was followed. Diethyl 2-ethylidenemalonate **2a** (29.7 mg, 0.16 mmol, 1.0 eq.), 2-oxo-2-(p-tolyl)acetic acid **1c** (52.8 mg, 0.32 mmol, 2.0 eq.), $(NH_4)_2S_2O_8$ (109.5 mg, 0.48 mmol, 3.0 eq.), γ-terpinene (51.3 µL, 0.32 mmol, 2.0 eq.), and 2,4,6-collidine **7** (42.3 µL, 0.32 mmol, 2.0 eq.) in DMSO (0.4 mL) was heated to 50 °C for 24 h. The crude was then purified *via* column flash chromatography eluting with 85:15 hexane:EtOAc. The solvent was then removed *in vacuo* to give diethyl 2-(1-oxo-1-(*p*-tolyl)propan-2-yl)malonate **3b** (37.6 mg, 0.12 mmol, 77%).

Using Conditions B: General procedure for Conditions B was followed. Diethyl 2-ethylidenemalonate **2a** (29.7 mg, 0.16 mmol, 1.0 eq.), 2-oxo-2-(p-tolyl)acetic acid **1c** (52.8 mg, 0.32 mmol, 2.0 eq.), $(NH_4)_2S_2O_8$ **7** (109.4 mg, 0.48 mmol, 3.0 eq.), γ-terpinene (51.3 µL, 0.32 mmol, 2.0 eq.), and 2,4,6-collidine (42.3 µL, 0.32 mmol, 2.0 eq.) in DMSO (0.4 mL) was irradiated with 450 nm for 5 h. The crude was then purified *via* column flash chromatography eluting with 93:7→90:10 hexane:EtOAc. The solvent was then removed *in vacuo* to give diethyl 2-(1-oxo-1-(*p*-tolyl)propan-2-yl)malonate **3b** (45.4 mg, 0.15 mmol, 93%).

Characterisations:

Appearance: Colourless oil. **^{1}H NMR (400 MHz, Chloroform-*d*):** δ ppm 7.90 (d, *J* = 8.0 Hz, 2H, ArH), 7.27 (d, *J* = 8.0 Hz, 2H, ArH), 4.30 – 4.05 (m, 5H, 2 x OCH_2, C<u>H</u>Me), 4.20 – 4.02 (m, 3H, C<u>H</u>Me, OCH_2), 3.97 (d, *J* = 11.0 Hz, 1H, C<u>H</u>($CO_2Et)_2$), 2.40 (s, 3H, CH_3), 1.31 (t, *J* = 7.0 Hz, 3H, $CH_2C\underline{H}_3$), 1.18 (d, *J* = 7.0 Hz, 3H, $CHC\underline{H}_3$), 1.15 (t, *J* = 7.0 Hz, 3H, $CH_2C\underline{H}_3$). **$^{13}C\{^1H\}$ NMR (101 MHz, Chloroform-*d*):** δ ppm 201.3 (C), 168.9 (C), 168.5 (C), 144.2 (C), 133.2 (C), 129.5 (CH), 128.8 (CH), 61.7 (CH_2 plus one overlapping CH_2), 55.1 (CH), 40.5 (CH), 21.7 (CH_3), 16.1 (CH_3), 14.2 (CH_3), 14.0 (CH_3). **IR:** ν_{max}/cm^{-1} 2981, 2938, 2361, 1747, 1729, 1678, 1607, 1573, 1462, 1408, 1368, 1312, 1291, 1237, 1179, 1153, 1139, 1118, 1095. **TLC:** R_f = 0.25 (92:8 hexanes:EtOAc). Data is consistent with the literature.[8]

Diethyl 2-(1-mesityl-1-oxopropan-2-yl)malonate (3c)

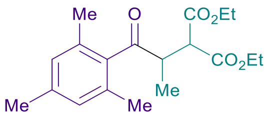

Using Conditions A: General procedure for Conditions A was followed. Diethyl 2-ethylidenemalonate **2a** (29.9 mg, 0.16 mmol, 1.0 eq.), 2-mesityl-2-oxoacetic acid **1d** (61.5 mg, 0.32 mmol, 2.0 eq.), $(NH_4)_2S_2O_8$ (109.5 mg, 0.48 mmol, 3.0 eq.), γ-terpinene (51.3 µL, 0.32 mmol, 2.0 eq.), and 2,4,6-collidine **7** (42.3 µL, 0.2 mmol, 2.0 eq.) in DMSO (0.4 mL) was heated to 50 °C for 24 h. The crude was then purified *via* column flash chromatography eluting with 95:5 hexane:EtOAc. The solvent was then removed *in vacuo* to give diethyl 2-(1-mesityl-1-oxopropan-2-yl)malonate **3c** (47.2 mg, 0.14 mmol, 88%).

Using Conditions B: General procedure for Conditions B was followed. Diethyl 2-ethylidenemalonate **2a** (29.7 mg, 0.16 mmol, 1.0 eq.), 2-mesityl-2-oxoacetic acid **1d** (62.0 mg, 0.32 mmol, 2.0 eq.), $(NH_4)_2S_2O_8$ (109.9 mg, 0.48 mmol, 3.0 eq.), γ-terpinene (51.3 µL, 0.32 mmol, 2.0 eq.), and 2,4,6-collidine **7** (42.3 µL, 0.2 mmol, 2.0 eq.) in DMSO (0.4 mL) was irradiated with 450 nm for 5 h. The crude was then purified *via* column flash chromatography eluting with 92:8 hexane:EtOAc. The solvent was then removed *in vacuo* to give diethyl 2-(1-mesityl-1-oxopropan-2-yl)malonate **3c** (47.2 mg, 0.14 mmol, 88%).

Characterisations:

Appearance: Colourless oil. **^{1}H NMR (400 MHz, Chloroform-*d*):** δ ppm 6.83 (s, 2H, ArH), 4.22 (q, *J* = 7.0 Hz, 4H, 2 x OCH_2), 3.82 (d, *J* = 9.0 Hz, 1H, C<u>H</u>(CO_2Et)$_2$), 3.78 (dq, *J* = 9.0, 7.5 Hz, 1H, C<u>H</u>Me), 2.27 (s, 9H, 3 x CH_3), 1.29 (t, *J* = 7.0 Hz, 3H, CH_2C<u>H</u>$_3$), 1.28 (t, *J* = 7.0 Hz, 3H, CH_2C<u>H</u>$_3$), 1.15 (d, *J* = 7.5 Hz, 3H, CHC<u>H</u>$_3$). **^{13}C{^{1}H} NMR (101 MHz, Chloroform-*d*):** δ ppm 209.5 (C), 168.72 (C), 168.66 (C), 138.9 (C), 136.6 (C), 134.2 (C), 129.0 (CH), 61.7 (CH_2 plus one overlapping CH_2), 53.7 (CH), 47.6 (CH), 21.1 (CH_3), 19.8 (CH_3), 14.21 (CH_3), 14.16 (CH_3), 13.8 (CH_3). **IR:** ν_{max}/cm^{-1} 2980, 2937, 2357, 2332, 1751, 1732, 1692, 1610, 1456, 1369, 1283, 1267, 1225, 1188, 1153. **TLC:** R_f = 0.11 (95:5 hexane:EtOAc). **HRMS (ESI-TOF):** *m*/*z* $[M + H]^+$ calcd for $C_{19}H_{27}O_5$, 335.1853; found, 335.1859.

Diethyl 2-(1-(4-methoxyphenyl)-1-oxopropan-2-yl)malonate (3d)

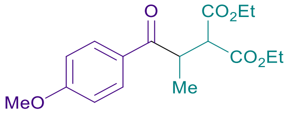

Using Conditions A: General procedure Conditions A was followed. Diethyl 2-ethylidenemalonate **2a** (29.8 mg, 0.16 mmol, 1.0 eq.), 2-(4-methoxyphenyl)-2-oxoacetic acid **1b** (57.9 mg, 0.32 mmol, 2.0 eq.), $(NH_4)_2S_2O_8$ (109.8 mg, 0.48 mmol, 3.0 eq.), γ-terpinene (51.3 μL, 0.32 mmol, 2.0 eq.), and 2,4,6-collidine **7** (42.3 μL, 0.32 mmol, 2.0 eq.) in DMSO (0.4 mL) was irradiated with 450 nm for 5 h. The crude was then purified *via* column flash chromatography eluting with 80:20 hexane:EtOAc. The solvent was then removed *in vacuo* to give diethyl 2-(1-(4-methoxyphenyl)-1-oxopropan-2-yl)malonate **3d** (48.5 mg, 0.14 mmol, 89%).

Using Conditions B: General procedure Conditions B was followed. Diethyl 2-ethylidenemalonate **2a** (29.8 mg, 0.16 mmol, 1.0 eq.), 2-(4-methoxyphenyl)-2-oxoacetic acid **1b** (57.9 mg, 0.32 mmol, 2.0 eq.), $(NH_4)_2S_2O_8$ (109.8 mg, 0.48 mmol, 3.0 eq.), γ-terpinene (51.3 μL, 0.32 mmol, 2.0 eq.), and 2,4,6-collidine **7** (42.3 μL, 0.32 mmol, 2.0 eq.) in DMSO (0.4 mL) was irradiated with 450 nm for 5 h. The crude was then purified *via* column flash chromatography eluting with 88:12 hexane:EtOAc. The solvent was then removed *in vacuo* to give diethyl 2-(1-(4-methoxyphenyl)-1-oxopropan-2-yl)malonate **3d** (50.2 mg, 0.16 mmol, 97%).

Using Conditions B on 1.2 mmol scale: A stock of DMSO was sparged with Ar (ballon) for 1 h. To a 20 ml-scintillation vial equipped with a magnetic stirrer bar was added diethyl 2-ethylidenemalonate **2a** (223.5 mg, 1.20 mmol, 1.0 eq.), 2-(4-methoxyphenyl)-2-oxoacetic acid **1b** (432.4 mg, 2.4 mmol, 2.0 eq.), $(NH_4)_2S_2O_8$ (821.7 mg, 3.6 mmol, 3.0 eq.), γ-terpinene (384.8 μL, 2.4 mmol, 2.0 eq.), and 2,4,6-collidine **7** (317.6 μL, 2.4 mmol, 2.0 eq.). After sparging, an aliquot of the DMSO stock (3 mL) was added to the vial and the resulting solution was sparged with more Ar (ballon) for 5 min. The vial was then quickly sealed tight and placed in a Penn PhD Photoreactor M2 (fan speed = 6800 rpm, light intensity = 100%, wavelength = 450 nm, temperature = 25 °C, stirring = 800 rpm) and the reaction mixture was irradiated with 450 nm blue LEDs for 5 h at rt with continuous stirring. After 5 h TLC (using 80:20 hexanes:EtOAc) showed complete consumption of starting material **2a**, thus the reaction mixture was diluted with CH_2Cl_2 (25 mL) and washed with sat. aq. $NaHCO_3$ (60 mL) and the

layers were separated. The aq. layer was extracted with more CH_2Cl_2 (3 x 20 mL), and the combined organic layers were washed with aq. 1 M HCl (60 mL) and the layers were separated. The acid aq. layer was extracted with more CH_2Cl_2 (20 mL), and the combined organic layers were dried over $MgSO_4$, filtered, and concentrated *in vacuo* to give the crude product. The crude was then purified *via* column flash chromatography eluting with 88:12→84:16 hexane:EtOAc. The solvent was then removed *in vacuo* to give diethyl 2-(1-(4-methoxyphenyl)-1-oxopropan-2-yl)malonate **3d** (332.4 mg, 1.0 mmol, 86%).

Using Conditions B on 3.0 mmol scale: A stock of DMSO was sparged with Ar (ballon) for 1 h. To a 20 ml-scintillation vial equipped with a magnetic stirrer bar was added diethyl 2-ethylidenemalonate **2a** (558.6 mg, 3.0 mmol, 1.0 eq.), 2-(4-methoxyphenyl)-2-oxoacetic acid **1b** (1.08 g, 6.0 mmol, 2.0 eq.), $(NH_4)_2S_2O_8$ (2.05 g, 3.60 mmol, 3.0 eq.), γ-terpinene (0.97 mL, 6.0 mmol, 2.0 eq.), and 2,4,6-collidine **7** (0.80 mL, 6.0 mmol, 2.0 eq.). After sparging, an aliquot of the DMSO stock (7.5 mL) was added to the vial and the resulting solution was sparged with more Ar (ballon) for 15 min. The vial was then quickly sealed tight and placed in a Penn PhD Photoreactor M2 (fan speed = 6800 rpm, light intensity = 100%, wavelength = 450 nm, temperature = 25 °C, stirring = 800 rpm) and the reaction mixture was irradiated with 450 nm blue LEDs for 5 h at rt with continuous stirring (see Figure S9 for reaction set-up). After 5 h TLC (using 80:20 hexanes:EtOAc) showed complete consumption of starting material **2a**, thus the reaction mixture was diluted with CH_2Cl_2 (35 mL) and washed with sat. aq. $NaHCO_3$ (70 mL) and the layers were separated. The aq. layer was extracted with more CH_2Cl_2 (3 x 20 mL), and the combined organic layers were washed with aq. 1 M HCl (60 mL) and the layers were separated. The acid aq. layer was extracted with more CH_2Cl_2 (20 mL), and the combined organic layers were dried over $MgSO_4$, filtered, and concentrated *in vacuo* to give the crude product. The crude was then purified *via* column flash chromatography eluting with 88:12→84:16 hexane:EtOAc. The solvent was then removed *in vacuo* to give diethyl 2-(1-(4-methoxyphenyl)-1-oxopropan-2-yl)malonate **3d** (809.1 mg, 2.5 mmol, 84%).

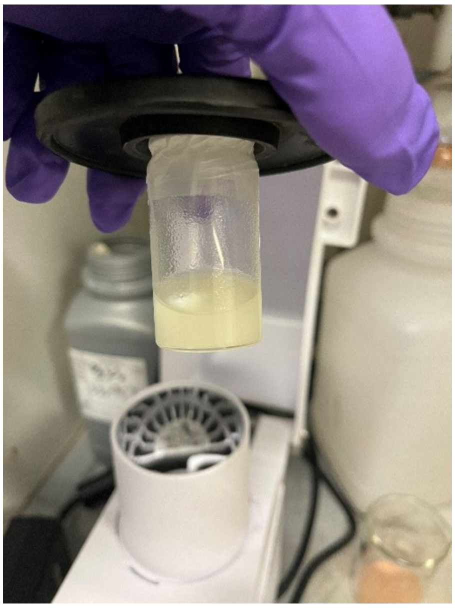

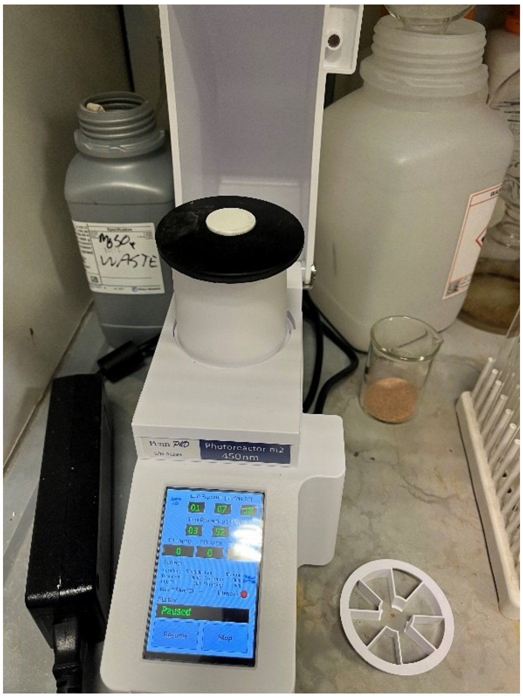

Figure S9. Reaction set-up for 1.2 mmol scale reaction. Similar set-up was applied for 3.0 mmol scale reaction.

Characterisations:

Appearance: Pale-green oil. **^{1}H NMR (400 MHz, Chloroform-*d*):** δ ppm 7.99 (d, *J* = 9.0 Hz, 2H, ArH), 6.95 (d, *J* = 9.0 Hz, 2H, ArH), 4.30 – 4.21 (m, 2H, OCH_2), 4.18 – 4.03 (m, 3H, $C\underline{H}Me$, OCH_2), 3.96 (d, *J* = 11.0 Hz, 1H, $C\underline{H}(CO_2Et)_2$), 3.86 (s, 3H, OCH_3), 1.31 (t, *J* = 7.0 Hz, 3H, $CH_2C\underline{H}_3$), 1.18 (d, *J* = 7.0 Hz, 3H, $CHC\underline{H}_3$), 1.15 (t, *J* = 7.0 Hz, 3H, $CH_2C\underline{H}_3$). **^{13}C{^{1}H} NMR (101 MHz, Chloroform-*d*):** δ ppm 200.2 (C), 169.0 (C), 168.5 (C), 163.8 (C), 131.0 (CH), 128.6 (C), 114.0 (CH), 61.7 (CH_2 plus one overlapping CH_2), 55.6 (CH_3), 55.1 (CH), 40.3 (CH), 16.2 (CH_3), 14.3 (CH_3), 14.0 (CH_3). **IR:** ν_{max}/cm^{-1} 2980, 2938, 2842, 1746, 1728, 1673, 1599, 1575, 1511, 1462, 1420, 1368, 1309, 1294, 1258, 1239, 1170. **TLC:** R_f = 0.21 (84:16 hexane:EtOAc). Data is consistent with the literature.[9]

Diethyl 2-(1-(4-(dimethylamino)phenyl)-1-oxopropan-2-yl)malonate (3e)

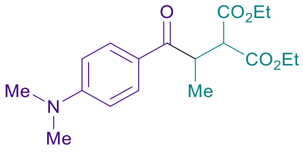

Using Conditions A: General procedure for Conditions A was followed. Diethyl 2-ethylidenemalonate **2a** (29.8 mg, 0.16 mmol, 1.0 eq.), 2-(4-(dimethylamino)phenyl)-2-oxoacetic acid **1e** (62.2 mg, 0.32 mmol, 2.0 eq.), $(NH_4)_2S_2O_8$ (109.5 mg, 0.48 mmol, 3.0 eq.), γ-terpinene (42.3 µL, 0.32 mmol, 2.0 eq.), and 2,4,6-collidine **7** (42.3 µL, 0.32 mmol, 2.0 eq.) in DMSO (0.4 mL) was heated to 50 °C for 24 h. The crude was then purified *via* column flash chromatography eluting with 70:30 hexane:EtOAc. The solvent was then removed *in vacuo* to give diethyl 2-(1-(4-(dimethylamino)phenyl)-1-oxopropan-2-yl)malonate **3e** (16.8 mg, 0.05 mmol, 31%).

Using Conditions B: General procedure for Conditions B was followed. Diethyl 2-ethylidenemalonate **2a** (22.3 mg, 0.12 mmol, 1.0 eq.), 2-(4-(dimethylamino)phenyl)-2-oxoacetic acid **1e** (46.4 mg, 0.24 mmol, 2.0 eq.), $(NH_4)_2S_2O_8$ (82.3 mg, 0.36 mmol, 3.0 eq.), γ-terpinene (38.5 µL, 0.24 mmol, 2.0 eq.), and 2,4,6-collidine **7** (31.8 µL, 0.24 mmol, 2.0 eq.) in DMSO (0.4 mL) was irradiated with 450 nm for 16 h. The crude was then purified *via* column flash chromatography eluting with 80:20 hexane:EtOAc. The solvent was then removed *in vacuo* to give diethyl 2-(1-(4-(dimethylamino)phenyl)-1-oxopropan-2-yl)malonate **3e** (21.9 mg, 0.09 mmol, 54%).

Characterisations:

Appearance: Pale yellow solid. **^{1}H NMR (400 MHz, Chloroform-*d*):** δ ppm 7.92 (d, J = 9.0 Hz, 2H, ArH), 6.66 (d, J = 9.0 Hz, 2H, ArH), 4.30 – 4.21 (m, 2H, OCH_2), 4.17 – 4.01 (m, 3H, C$\underline{H}$$CH_3$, OCH_2), 3.97 (d, J = 11.0 Hz, 1H, C$\underline{H}$$(CO_2Et)_2$), 3.05 (s, 6H, 2 x NCH_3), 1.31 (t, J = 7.0 Hz, 3H, $CH_2C\underline{H}_3$), 1.19 (d, J = 7.0 Hz, 3H, $CHC\underline{H}_3$), 1.15 (t, J = 7.0 Hz, 3H, $CH_2C\underline{H}_3$). **^{13}C{^{1}H} NMR (101 MHz, Chloroform-*d*):** δ ppm 199.6 (C), 169.5 (C), 168.8 (C), 153.9 (C), 131.1 (CH), 123.5 (C), 111.1 (CH), 61.8 (CH_2), 61.76 (CH_2), 55.3 (CH), 40.3 (CH_3), 40.1 (CH), 16.8 (CH_3), 14.5 (CH_3), 14.2 (CH_3). **IR:** ν_{max}/cm^{-1} 2976, 2828, 2358, 2323, 1745, 1725, 1647, 1607, 1548, 1459, 1447, 1385, 1370, 1293, 1273, 1234, 1173, 1144, 1133. **TLC:** R_f = 0.22

(80:20 hexanes:EtOAc). **HRMS (ESI-TOF):** *m/z* $[M + H]^+$ calcd for $C_{18}H_{26}NO_5$, 336.1806; found, 336.1811. **M.p.:** 86-89 °C**.**

Diethyl 2-(1-(4-fluorophenyl)-1-oxopropan-2-yl)malonate (3f)

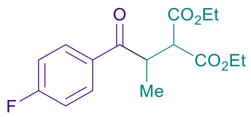

Using Conditions A: General procedure for Conditions A was followed. Diethyl 2-ethylidenemalonate **2a** (29.9 mg, 0.16 mmol, 1.0 eq.), 2-(4-fluorophenyl)-2-oxoacetic acid **1f** (53.8 mg, 0.32 mmol, 2.0 eq.), $(NH_4)_2S_2O_8$ (109.5 mg, 0.48 mmol, 3.0 eq.), γ-terpinene (42.3 µL, 0.32 mmol, 2.0 eq.), and 2,4,6-collidine **7** (42.3 µL, 0.32 mmol, 2.0 eq.) in DMSO (0.4 mL) was heated to 50 °C for 24 h. The crude was then purified *via* column flash chromatography eluting with 90:10 hexane:EtOAc. The solvent was then removed *in vacuo* to give diethyl 2 diethyl 2-(1-(4-fluorophenyl)-1-oxopropan-2-yl)malonate **3f** (23.0 mg, 0.07 mmol, 46%).

Using Conditions B: General procedure for Conditions B was followed. Diethyl 2-ethylidenemalonate **2a** (29.7 mg, 0.16 mmol, 1.0 eq.), 2-(4-fluorophenyl)-2-oxoacetic acid **1f** (53.8 mg, 0.32 mmol, 2.0 eq.), $(NH_4)_2S_2O_8$ (109.9 mg, 0.48 mmol, 3.0 eq.), γ-terpinene (51.3 µL, 0.32 mmol, 2.0 eq.), and 2,4,6-collidine (42.3 µL, 0.32 mmol, 2.0 eq.) in DMSO (0.4 mL) was irradiated with 450 nm for 5 h. The crude was then purified *via* column flash chromatography eluting with 91:9 hexane:EtOAc. The solvent was then removed *in vacuo* to give diethyl 2-(1-(4-fluorophenyl)-1-oxopropan-2-yl)malonate **3f** (45.5 mg, 0.15 mmol, 92%).

Characterisations:

Appearance: Colourless oil. **^{1}H NMR (400 MHz, Chloroform-*d*):** δ ppm 8.03 (dd, J = 9.0, 5.4 Hz, 2H, ArH), 7.14 (t, J = 9.0 Hz, 2H, ArH), 4.30 – 4.22 (m, 2H, OCH_2), 4.17 – 4.04 (m, 3H, OCH_2, C<u>H</u>Me), 3.97 (d, J = 11.0 Hz, 1H, C<u>H</u>($CO_2Et)_2$), 1.31 (t, J = 7.0 Hz, 3H, $CH_2C\underline{H}_3$), 1.18 (d, J = 7.0 Hz, 3H, $CHC\underline{H}_3$), 1.16 (t, J = 7.0 Hz, 3H, $CH_2C\underline{H}_3$). **^{13}C{^{1}H} NMR (101 MHz, Chloroform-*d*):** δ ppm 200.2 (C), 168.8 (C), 168.5 (C), 166.0 (C, d, J = 255.0 Hz), 132.1 (C, d, J = 3.0 Hz), 131.3 (d, J = 9.3 Hz, CH), 116.0 (d, J = 21.8 Hz, CH), 61.9 (CH_2), 61.8 (CH_2), 55.1 (CH), 40.5 (CH), 16.0 (CH_3), 14.3 (CH_3), 14.0 (CH_3). **^{19}F NMR (376 MHz, Chloroform-*d*):** δ ppm -105.06. **IR:** ν_{max}/cm^{-1} 3052, 2982, 2940, 1746, 1728, 1682, 1596, 1507, 1456, 1411, 1368, 1290, 1230, 1186, 1158, 1095. **TLC:** R_f = 0.25 (90:10 hexanes:EtOAc). **HRMS (ESI-**

TOF): *m/z* $[M + H]^+$ calcd for $C_{16}H_{20}FO_5$, 311.1289; found, 311.1286. Data is consistent with the literature.[10]

Diethyl 2-(1-(4-chlorophenyl)-1-oxopropan-2-yl)malonate (3g)

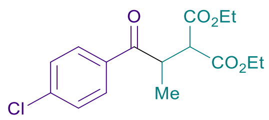

Using Conditions A: General procedure for Conditions A was followed. Diethyl 2-ethylidenemalonate **2a** (29.8 mg, 0.16 mmol, 1.0 eq.), 2-(4-chlorophenyl)-2-oxoacetic acid **1g** (88.6 mg, 0.48 mmol, 3.0 eq.), $(NH_4)_2S_2O_8$ (146.1 mg, 0.64 mmol, 4.0 eq.), γ-terpinene (51.3 µL, 0.32 mmol, 2.0 eq.), and 2,4,6-collidine **7** (63.5 µL, 0.48 mmol, 3.0 eq.) in DMSO (0.4 mL) was heated to 75 °C for 24 h. The crude was then purified *via* column flash chromatography eluting with 85:15 hexane:EtOAc. The solvent was then removed *in vacuo* to give diethyl 2-(1-(4-chlorophenyl)-1-oxopropan-2-yl)malonate **3g** (13.9 mg, 0.04 mmol, 27%).

Using Conditions B: General procedure for Conditions **B** was followed. Diethyl 2-ethylidenemalonate **2a** (22.3 mg, 0.12 mmol, 1.0 eq.), 2-(4-chlorophenyl)-2-oxoacetic acid **1g** (66.4 mg, 0.32 mmol, 3.0 eq.), $(NH_4)_2S_2O_8$ (109.9 mg, 0.48 mmol, 4.0 eq.), γ-terpinene (38.5 µL, 0.24 mmol, 2.0 eq.), and 2,4,6-collidine (48.0 µL, 0.36 mmol, 3.0 eq.) in DMSO (0.3 mL) was irradiated with 450 nm for 16 h. The crude was then purified *via* column flash chromatography eluting with 93:7 hexane:EtOAc. The solvent was then removed *in vacuo* to give diethyl 2-(1-(4-chlorophenyl)-1-oxopropan-2-yl)malonate **3g** (15.5 mg, 0.05 mmol, 40%).

Characterisations:

Appearance: Colourless oil. **^{1}H NMR (400 MHz, Chloroform-*d*):** δ ppm 7.88 (d, *J* = 8.5 Hz, 2H, ArH), 7.39 (d, *J* = 8.5 Hz, 2H, ArH), 4.26 – 4.15 (m, 2H, OCH_2), 4.10 – 3.98 (m, 3H, C<u>H</u>Me, OCH_2), 3.90 (d, *J* = 11.0 Hz, 1H, C<u>H</u>($CO_2Et)_2$), 1.25 (t, *J* = 7.0 Hz, 3H, $CH_2C\underline{H}_3$), 1.15 – 1.04 (m, 6H, $CHC\underline{H}_3$, $CH_2C\underline{H}_3$). **^{13}C{^{1}H} NMR (101 MHz, Chloroform-*d*):** δ ppm 200.6 (C), 168.8 (C), 168.5 (C), 139.9 (C), 134.1 (C), 130.1 (CH), 129.2 (CH), 60.90 (CH_2), 60.85 (CH_2), 55.1 (CH), 40.6 (CH), 15.9 (CH_3), 14.3 (CH_3), 14.0 (CH_3). **IR:** ν_{max}/cm^{-1} 2982, 2938, 2907, 2878, 1745, 1728, 1686, 1683, 1589, 1571, 1489, 1462, 1401, 1368, 1290, 1234, 1186, 1091. **TLC:** R_f = 0.28 (90:10 hexanes:EtOAc). **HRMS (ESI-TOF):** *m/z* $[M + H]^+$ calcd for $C_{16}H_{20}{}^{35}ClO_5$, 327.0993; found, 327.0992.

Diethyl 2-(1-oxo-1-(thiophen-2-yl)propan-2-yl)malonate (3h)

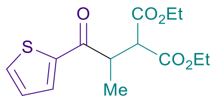

Using Conditions A: General procedure for Conditions A was followed. Diethyl 2-ethylidenemalonate **2a** (29.8 mg, 0.16 mmol, 1.0 eq.), 2-oxo-2-(thiophen-2-yl)acetic acid **1h** (49.9 mg, 0.32 mmol, 2.0 eq.), $(NH_4)_2S_2O_8$ (109.6 mg, 0.48 mmol, 3.0 eq.), γ-terpinene (51.3 μL, 0.32 mmol, 2.0 eq.), and 2,4,6-collidine **7** (42.3 μL, 0.32 mmol, 2.0 eq.) in DMSO (0.4 mL) was heated to 50 °C for 24 h. The crude was then purified *via* column flash chromatography eluting with 85:15 hexane:EtOAc. The solvent was then removed *in vacuo* to give diethyl 2-(1-oxo-1-(thiophen-2-yl)propan-2-yl)malonate **3h** (26.0 mg, 0.09 mmol, 54%).

Using Conditions B: General procedure for Condition B was followed. Diethyl 2-ethylidenemalonate **2a** (29.7 mg, 0.16 mmol, 1.0 eq.), 2-oxo-2-(thiophen-2-yl)acetic acid **1h** (50.0 mg, 0.32 mmol, 2.0 eq.), $(NH_4)_2S_2O_8$ (109.6 mg, 0.48 mmol, 3.0 eq.), γ-terpinene (51.3 μL, 0.32 mmol, 2.0 eq.), and 2,4,6-collidine (42.3 μL, 0.32 mmol, 2.0 eq.) in DMSO (0.4 mL) was irradiated with 450 nm for 5 h. The crude was then purified *via* column flash chromatography eluting with 78:12 hexane:EtOAc. The solvent was then removed *in vacuo* to give diethyl 2-(1-oxo-1-(thiophen-2-yl)propan-2-yl)malonate **3h** (26.3 mg, 0.09 mmol, 55%).

Characterisations:

Appearance: Pale yellow oil. **^{1}H NMR (400 MHz, Chloroform-*d*):** δ ppm 7.82 (dd, *J* = 4.0, 1.0 Hz, 1H, ArH), 7.66 (dd, *J* = 5.0, 1.0 Hz, 1H, ArH), 7.15 (dd, *J* = 5.0, 4.0 Hz, 1H, ArH), 4.30 – 4.21 (m, 2H, OCH_2), 4.18 – 4.04 (m, 2H, OCH_2), 4.02 – 3.92 (m, 2H, C$\underline{H}$(CO_2Et)$_2$, C$\underline{H}$Me), 1.31 (t, *J* = 7.0 Hz, 3H, $OCH_2C\underline{H}_3$), 1.26 (d, *J* = 6.5 Hz, 3H, $C\underline{H}CH_3$), 1.15 (t, *J* = 7.0 Hz, 3H, $OCH_2C\underline{H}_3$). **^{13}C{^{1}H} NMR (101 MHz, Chloroform-*d*):** δ ppm 194.4 (C), 168.7 (C), 168.3 (C), 142.8 (C), 134.2 (CH), 132.5 (CH), 128.4 (CH), 61.85 (CH_2), 61.82 (CH_2), 54.8 (CH), 42.2 (CH), 16.6 (CH_3), 14.3 (CH_3), 14.0 (CH_3). **IR:** ν_{max}/cm^{-1} 3102, 2981, 2939, 2363, 1746, 1728, 1659, 1519, 1462, 1414, 1368, 1294, 1240, 1214, 1184, 1155. **TLC:** R_f = 0.34 (75:15 hexanes:EtOAc). **HRMS (ESI-TOF):** *m/z* [M + H]$^+$ calcd for $C_{14}H_{19}O_5S$, 299.0948; found, 299.0959.

Diethyl 2-(1-oxo-1-(1H-pyrrol-2-yl)propan-2-yl)malonate (3i)

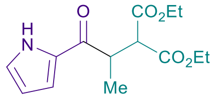

Using Conditions A: General procedure for Conditions A was followed. Diethyl 2-ethylidenemalonate **2a** (29.7 mg, 0.16 mmol, 1.0 eq.), 2-oxo-2-(1H-pyrrol-2-yl)acetic acid **1i** (44.8 mg, 0.32 mmol, 2.0 eq.), $(NH_4)_2S_2O_8$ (109.9 mg, 0.48 mmol, 3.0 eq.), γ-terpinene (51.3 μL, 0.32 mmol, 2.0 eq.), and 2,4,6-collidine **7** (42.3 μL, 0.32 mmol, 2.0 eq.) in DMSO (0.4 mL) was heated to 50 °C for 24 h. The crude was then purified *via* column flash chromatography eluting with 80:20 hexane:EtOAc. The solvent was then removed *in vacuo* to give diethyl 2-(1-oxo-1-(1H-pyrrol-2-yl)propan-2-yl)malonate **3i** (29.4 mg, 0.11 mmol, 66%).

Using Conditions B: General procedure for Conditions B was followed. Diethyl 2-ethylidenemalonate **2a** (22.3 mg, 0.12 mmol, 1.0 eq.), 2-oxo-2-(1H-pyrrol-2-yl)acetic acid **1i** (33.5 mg, 0.24 mmol, 2.0 eq.), $(NH_4)_2S_2O_8$ (82.2 mg, 0.36 mmol, 3.0 eq.), γ-terpinene (38.5 μL, 0.24 mmol, 2.0 eq.), and 2,4,6-collidine (31.8 μL, 0.24 mmol, 2.0 eq.) in DMSO (0.3 mL) was irradiated with 450 nm for 16 h. The crude was then purified *via* column flash chromatography eluting with 82:18 hexane:EtOAc. The solvent was then removed *in vacuo* to give diethyl 2-(1-oxo-1-(1H-pyrrol-2-yl)propan-2-yl)malonate **3i** (19.9 mg, 0.07 mmol, 57%).

Characterisations:

Appearance: Pale green oil. **^{1}H NMR (400 MHz, Chloroform-*d*):** δ ppm 9.65 (br s, 1H, NH), 7.09 – 7.00 (m, 2H, ArH), 6.32 – 6.27 (m, 1H, ArH), 4.30 – 2.30 (m, 2H, OCH_2), 4.18 – 3.99 (m, 2H, OCH_2), 3.96 – 3.82 (m, 2H, C<u>H</u>Me, C<u>H</u>$(CO_2Et)_2$), 1.30 (t, J = 7.0 Hz, 3H, $CH_2C\underline{H}_3$), 1.25 (d, J = 6.5 Hz, 2H, $CHC\underline{H}_3$), 1.13 (t, J = 7.0 Hz, 3H, $CH_2C\underline{H}_3$). **^{13}C{^{1}H} NMR (101 MHz, Chloroform-*d*):** δ ppm 191.4 (C), 168.9 (C), 168.3 (C), 130.6 (C), 125.3 (CH), 116.9 (CH), 111.0 (CH), 61.7 (CH_2), 61.7 (CH_2), 54.7 (CH), 41.0 (CH), 17.2 (CH_3), 14.3 (CH_3), 14.0 (CH_3). **IR:** ν_{max}/cm^{-1} 3290, 2980, 2939, 2361, 2343, 1728, 1634, 1546, 1462, 1408, 1369, 1308, 1274, 1196, 1141, 1097. **TLC:** R_f = 0.29 (80:20 hexane:EtOAc). **HRMS (ESI-TOF):** *m/z* [M + H]$^+$ calcd for $C_{14}H_{20}NO_5$, 282.1336; found, 282.1343.

Diethyl 2-(1-(1-methyl-1*H*-pyrrol-2-yl)-1-oxopropan-2-yl)malonate (3j)

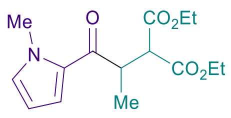

Using Conditions A: General procedure for Conditions A was followed. Diethyl 2-ethylidenemalonate **2a** (18.5 mg, 0.10 mmol, 1.0 eq.), 2-(1-methyl-1*H*-pyrrol-2-yl)-2-oxoacetic acid **1j** (30.4 mg, 0.20 mmol, 2.0 eq.), $(NH_4)_2S_2O_8$ (68.4 mg, 0.30 mmol, 3.0 eq.), γ-terpinene (32.1 µL, 0.20 mmol, 2.0 eq.), and 2,4,6-collidine **7** (26.5 µL, 0.20 mmol, 2.0 eq.) in DMSO (0.25 mL) was heated to 50 °C for 24 h. The crude was then purified *via* column flash chromatography eluting with 80:20 hexane:EtOAc. The solvent was then removed *in vacuo* to give diethyl 2-(1-(1-methyl-1*H*-pyrrol-2-yl)-1-oxopropan-2-yl)malonate **3j** (23.5 mg, 0.08 mmol, 79%).

Using Conditions B: General procedure for Conditions B was followed. Diethyl 2-ethylidenemalonate **2a** (22.4 mg, 0.12 mmol, 1.0 eq.), 2-(1-methyl-1*H*-pyrrol-2-yl)-2-oxoacetic acid **1j** (36.7 mg, 0.24 mmol, 2.0 eq.), $(NH_4)_2S_2O_8$ (82.4 mg, 0.36 mmol, 3.0 eq.), γ-terpinene (38.5 µL, 0.24 mmol, 2.0 eq.), and 2,4,6-collidine (31.8 µL, 0.24 mmol, 2.0 eq.) in DMSO (0.3 mL) was irradiated with 450 nm for 16 h. During work-up, brine (60 mL) was used instead of aq. 1M HCl. The crude was then purified *via* column flash chromatography eluting with 88:12 hexane:EtOAc. The solvent was then removed *in vacuo* to give diethyl 2-(1-(1-methyl-1*H*-pyrrol-2-yl)-1-oxopropan-2-yl)malonate **3j** (28.0 mg, 0.10 mmol, 79%).

Characterisations:

Appearance: Pale brown oil. **^{1}H NMR (400 MHz, Chloroform-*d*):** δ ppm 7.07 (dd, *J* = 4.0, 1.5 Hz, 1H, ArH), 6.81 (t, *J* = 2.0 Hz, 1H, ArH), 6.14 (dd, *J* = 4.0, 2.5 Hz, 1H, ArH), 4.31 – 4.18 (m, 2H, OCH_2), 4.18 – 4.02 (m, 2H, OCH_2), 3.95 – 3.84 (m, 5H, C<u>H</u>$(CO_2Et)_2$, C<u>H</u>Me, NCH_3), 1.30 (t, *J* = 7.0 Hz, 3H, $CH_2C\underline{H}_3$), 1.25 – 1.21 (m, 3H, $CHC\underline{H}_3$), 1.16 (t, *J* = 7.0 Hz, 3H, $CH_2C\underline{H}_3$). **^{13}C{^{1}H} NMR (101 MHz, Chloroform-*d*):** δ ppm 191.9 (C), 169.1 (C), 168.5 (C), 131.6 (CH), 129.5 (C), 119.7 (CH), 108.4 (CH), 61.64 (CH_2), 61.57 (CH_2), 54.8 (CH), 41.8 (CH), 37.8 (CH_3), 17.3 (CH_3), 14.3 (CH_3), 14.0 (CH_3). **IR:** ν_{max}/cm^{-1} 3113, 2980, 2939, 2363, 1748, 1729, 1645, 1528, 1464, 1407, 1386, 1368, 1324, 1275, 1240, 1185, 1140, 1094.

TLC: R_f = 0.39 (80:20 hexanes:EtOAc). **HRMS (ESI-TOF):** *m*/*z* $[M + H]^+$ calcd for $C_{15}H_{22}NO_5$, 296.1493; found, 296.1500.

Diethyl 2-(1-(2-methyl-1H-indol-3-yl)-1-oxopropan-2-yl)malonate (3k)

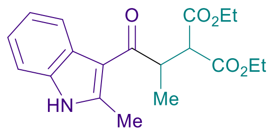

Using Conditions A: General procedure for Conditions A was followed. Diethyl 2-ethylidenemalonate **2a** (18.6 mg, 0.10 mmol, 1.0 eq.), 2-(2-methyl-1H-indol-3-yl)-2-oxoacetic acid **1k** (40.6 mg, 0.20 mmol, 2.0 eq.), $(NH_4)_2S_2O_8$ (68.4 mg, 0.30 mmol, 3.0 eq.), γ-terpinene (32.1 μL, 0.20 mmol, 2.0 eq.), and 2,4,6-collidine **7** (26.5 μL, 0.20 mmol, 2.0 eq.) in DMSO (0.25 mL) was heated to 50 °C for 24 h. The crude was then purified *via* column flash chromatography eluting with 70:30 hexane:EtOAc. The solvent was then removed *in vacuo* to give diethyl 2-(1-(2-methyl-1H-indol-3-yl)-1-oxopropan-2-yl)malonate **3k** (20.5 mg, 0.06 mmol, 59%).

Using Conditions B: General procedure for Conditions B was followed. Diethyl 2-ethylidenemalonate **2a** (22.4 mg, 0.12 mmol, 1.0 eq.), 2-(2-methyl-1H-indol-3-yl)-2-oxoacetic acid **1k** (48.9 mg, 0.24 mmol, 2.0 eq.), $(NH_4)_2S_2O_8$ (82.1 mg, 0.36 mmol, 3.0 eq.), γ-terpinene (38.5 μL, 0.24 mmol, 2.0 eq.), and 2,4,6-collidine (31.8 μL, 0.24 mmol, 2.0 eq.) in DMSO (0.3 mL) was irradiated with 450 nm for 5 h. The crude was then purified *via* column flash chromatography eluting with 69:31 hexane:EtOAc. The solvent was then removed *in vacuo* to give diethyl 2-(1-(2-methyl-1H-indol-3-yl)-1-oxopropan-2-yl)malonate **3k** (29.7 mg, 0.09 mmol, 71%).

Characterisations:

Appearance: Brown solid. **^{1}H NMR (400 MHz, Chloroform-*d*):** δ ppm 8.97 (br s, 1H, NH), 7.97 (d, *J* = 8.0 Hz, 1H, ArH), 7.30 – 7.25 (m, 1H, ArH), 7.24 – 7.12 (m, 2H, ArH), 4.34 – 4.25 (m, 2H, OCH_2), 4.22 – 4.05 (m, 4H, OCH_2, C<u>H</u>Me, C<u>H</u>($CO_2Et)_2$), 2.55 (s, 3H, CH_3), 1.34 (t, *J* = 7.0 Hz, 3H, $CH_2C\underline{H}_3$), 1.29 (d, *J* = 6.5 Hz, 3H, $CHC\underline{H}_3$), 1.18 (t, *J* = 7.0 Hz, 3H, $CH_2C\underline{H}_3$). **$^{13}C\{^1H\}$ NMR (101 MHz, Chloroform-*d*):** δ ppm 197.8 (C), 169.5 (C), 169.2 (C), 145.1 (C), 134.9 (C), 126.5 (C), 122.5 (CH), 122.3 (CH), 120.8 (CH), 112.5 (C), 111.2 (CH), 61.7 (CH_2 plus one overlapping CH_2), 54.9 (CH), 43.9 (CH), 15.8 (CH_3), 15.5 (CH_3), 14.3 (CH_3), 14.0 (CH_3). **IR:** ν_{max}/cm^{-1} 3312, 2980, 2937, 2361, 1729, 1634, 1616, 1527, 1485, 1456, 1429, 1389, 1368, 1302, 1277, 1247, 1201, 1168, 1155, 1112. **TLC:** R_f = 0.17 (80:20 hexanes:EtOAc).

HRMS (ESI-TOF): m/z $[M + H]^+$ calcd for $C_{19}H_{24}NO_5$, 346.1649; found, 346.1646. **M.p.:** 81-84 °C.

Diethyl 2-(4-methoxybenzoyl)succinate (3m)

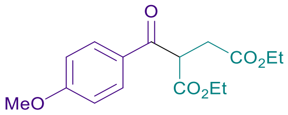

*Using diethyl maleate **2b**:*

Using Conditions B: General procedure for Conditions B was followed. Diethyl maleate **2b** (27.5 mg, 0.16 mmol, 1.0 eq.), 2-(4-methoxyphenyl)-2-oxoacetic acid **1b** (57.7 mg, 0.48 mmol, 2.0 eq.), $(NH_4)_2S_2O_8$ (109.5 mg, 0.48 mmol, 3.0 eq.), γ-terpinene (51.3 µL, 0.36 mmol, 2.0 eq.), and 2,4,6-collidine (42.3 µL, 0.24 mmol, 2.0 eq.) in DMSO (0.4 mL) was irradiated with 450 nm for 5 h. The crude was then purified *via* column flash chromatography eluting with 84:16 hexane:EtOAc. The solvent was then removed *in vacuo* to give diethyl 2-(4-methoxybenzoyl)succinate **3m** (36.6 mg, 0.12 mmol, 74%).

*Using diethyl maleate **2c**:*

Using Conditions B: General procedure for Conditions B was followed. Diethyl fumarate **2c** (27.7 mg, 0.16 mmol, 1.0 eq.), 2-(4-methoxyphenyl)-2-oxoacetic acid **1b** (57.7 mg, 0.48 mmol, 2.0 eq.), $(NH_4)_2S_2O_8$ (109.8 mg, 0.48 mmol, 3.0 eq.), γ-terpinene (51.3 µL, 0.36 mmol, 2.0 eq.), and 2,4,6-collidine (42.3 µL, 0.24 mmol, 2.0 eq.) in DMSO (0.4 mL) was irradiated with 450 nm for 5 h. The crude was then purified *via* column flash chromatography eluting with 84:16 hexane:EtOAc. The solvent was then removed *in vacuo* to give diethyl 2-(4-methoxybenzoyl)succinate **3m** (32.5 mg, 0.11 mmol, 66%).

Characterisations:

Appearance: Colourless oil. **^{1}H NMR (400 MHz, Chloroform-*d*):** δ ppm 8.02 (d, J = 9.0 Hz, 1H, ArH), 6.94 (d, J = 9.0 Hz, 1H, ArH), 4.80 (t, J = 7.2 Hz, 1H, C<u>H</u>CO_2Et), 4.17 – 4.08 (m, 4H, 2 x OCH_2), 3.87 (s, 3H, OCH_3), 3.05 (dd, J = 17.5 Hz, 7.4 Hz, 1H, C<u>H</u>HCO_2Et), 3.00 (dd, J = 17.5 Hz, 7.0 Hz, 1H, CH<u>H</u>CO_2Et), 1.21 (t, J = 7.0 Hz, 3H, CH_2C<u>H</u>$_3$), 1.16 (t, J = 7.0 Hz, 3H, CH_2C<u>H</u>$_3$). **^{13}C{^{1}H} NMR (101 MHz, Chloroform-5*d*):** δ ppm 192.6 (C), 171.5 (C), 169.0 (C), 164.1 (C), 131.4 (CH), 129.0 (C), 114.0 (CH), 61.8 (CH_2), 61.1 (CH_2), 55.6 (CH), 49.5 (CH_3), 33.4 (CH_2), 14.2 (CH_3), 14.0 (CH_3). **IR:** ν_{max}/cm^{-1} 2981, 2939, 2842, 1729, 1674, 1598,

1575, 1512, 1464, 1421, 1393, 1250, 1166. **TLC:** R_f = 0.20 (80:20 hexanes:EtOAc). Data is consistent with the literature.[11]

1-(4-Methoxyphenyl)-3-(phenylsulfonyl)propan-1-one (3n)

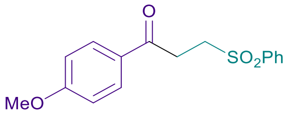

Using Conditions A: General procedure for Conditions A was followed. Phenyl vinyl sulfone **2d** (27.1 mg, 0.16 mmol, 1.0 eq.), 2-(4-methoxyphenyl)-2-oxoacetic acid **1b** (58.1 mg, 0.32 mmol, 2.0 eq.), $(NH_4)_2S_2O_8$ (109.7 mg, 0.48 mmol, 3.0 eq.), γ-terpinene (51.3 μL, 0.32 mmol, 2.0 eq.), and 2,4,6-collidine **7** (42.3 μL, 0.32 mmol, 2.0 eq.) in DMSO (0.4 mL) was heated to 50 °C for 24 h. The crude was then purified *via* column flash chromatography eluting with 75:25 hexane:EtOAc. The solvent was then removed *in vacuo* to give 1-(4-methoxyphenyl)-3-(phenylsulfonyl)propan-1-one **3n** (25.1 mg, 0.08 mmol, 51%).

Using Conditions B: General procedure for Conditions B was followed. Phenyl vinyl sulfone **2d** (26.9 mg, 0.16 mmol, 1.0 eq.), 2-(4-methoxyphenyl)-2-oxoacetic acid **1b** (57.9 mg, 0.48 mmol, 2.0 eq.), $(NH_4)_2S_2O_8$ (109.8 mg, 0.48 mmol, 3.0 eq.), γ-terpinene (51.3 μL, 0.36 mmol, 2.0 eq.), and 2,4,6-collidine (42.3 μL, 0.24 mmol, 2.0 eq.) in DMSO (0.4 mL) was irradiated with 450 nm for 5 h. The crude was then purified *via* column flash chromatography eluting with 75:25 hexane:EtOAc, followed by a second purification *via* flash column chromatography eluting with 78:22 hexane:EtOAc. The solvent was then removed *in vacuo* to give 1-(4-methoxyphenyl)-3-(phenylsulfonyl)propan-1-one **3n** (27.4 mg, 0.09 mmol, 56%).

Characterisations:

Appearance: White solid. **^{1}H NMR (400 MHz, Chloroform-*d*):** δ ppm 7.98 – 7.87 (m, 4H, ArH), 7.67 (t, *J* = 7.5 Hz, 1H, ArH), 7.57 (t, *J* = 7.5 Hz, 2H, ArH), 6.93 (d, *J* = 9.0 Hz, 2H, ArH), 3.87 (s, 3H, OCH_3), 3.59 – 3.51 (m, 2H, CH_2), 3.48 – 3.39 (m, 2H, CH_2). **^{13}C{^{1}H} NMR (101 MHz, Chloroform-*d*):** δ ppm 194.0 (C), 164.1 (C), 139.3 (C), 134.0 (CH), 130.5 (CH), 129.5 (CH), 129.0 (C), 128.1 (CH), 114.1 (CH), 55.7 (CH_3), 51.3 (CH_2), 31.1 (CH_2). **IR:** ν_{max}/cm^{-1} 3063, 3017, 2991, 2915, 2846, 2161, 1686, 1678, 1602, 1575, 1511, 1454, 1447, 1419, 1348, 1318, 1272, 1252, 1182, 1149. **TLC:** R_f = 0.18 (70:30 hexanes:EtOAc). **M.p.:** 101-105 °C. Data is consistent with the literature.[9]

1-(4-Methoxyphenyl)-3-(methylsulfonyl)propan-1-one (3o)

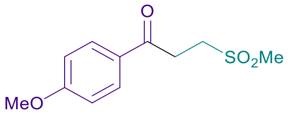

Using Conditions B: General procedure for Conditions B was followed. Methyl vinyl sulfone **2e** (17.1 mg, 0.16 mmol, 1.0 eq.), 2-(4-methoxyphenyl)-2-oxoacetic acid **1b** (57.6 mg, 0.32 mmol, 2.0 eq.), $(NH_4)_2S_2O_8$ (109.6 mg, 0.48 mmol, 3.0 eq.), γ-terpinene (51.3 µL, 0.32 mmol, 2.0 eq.), and 2,4,6-collidine (42.3 µL, 0.32 mmol, 2.0 eq.) in DMSO (0.4 mL) was irradiated with 450 nm for 5 h. The crude was then purified *via* column flash chromatography eluting with 53:47 hexane:EtOAc. The solvent was then removed *in vacuo* to give 1-(4-methoxyphenyl)-3-(methylsulfonyl)propan-1-one **3o** (26.4 mg, 0.11 mmol, 68%).

Characterisations:

Appearance: Off-white solid. **^{1}H NMR (400 MHz, Chloroform-*d*):** δ ppm 7.96 (d, *J* = 9.0 Hz, 2H, ArH), 6.95 (d, *J* = 9.0 Hz, 2H, ArH), 3.88 (s, 3H, OCH_3), 3.56 – 3.45 (m, 4H, 2 x CH_2), 2.98 (s, 3H, SO_2CH_3). **^{13}C{^{1}H} NMR (101 MHz, Chloroform-*d*):** δ ppm 194.1 (C), 164.3 (C), 130.6 (CH), 128.9 (C), 114.1 (CH), 55.7 (CH_3), 49.6 (CH_2), 41.8 (CH_3), 30.9 (CH_2). **IR:** ν_{max}/cm^{-1} 3008, 2966, 2924, 2942, 2842, 2160, 2050, 1678, 1601, 1516, 1463, 1446, 1421, 1364, 1305, 1255, 1206, 1185, 1170, 1127. **TLC:** R_f = 0.18 (55:45 hexanes:EtOAc). **M.p.:** 136-139 °C. Data is consistent with the literature.[12]

Diethyl (3-(4-methoxyphenyl)-3-oxopropyl)phosphonate (3p)

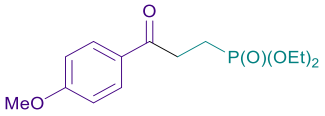

Using Conditions B: General procedure for Conditions B was followed. Diethyl vinyl phosphonate **2f** (26.3 mg, 0.16 mmol, 1.0 eq.), 2-(4-methoxyphenyl)-2-oxoacetic acid **1b** (57.7 mg, 0.48 mmol, 2.0 eq.), $(NH_4)_2S_2O_8$ (109.9 mg, 0.48 mmol, 3.0 eq.), γ-terpinene (51.3 μL, 0.36 mmol, 2.0 eq.), and 2,4,6-collidine (42.3 μL, 0.24 mmol, 2.0 eq.) in DMSO (0.4 mL) was irradiated with 450 nm for 5 h. The crude was then purified *via* column flash chromatography eluting with 90:10 hexane:EtOAc. The solvent was then removed *in vacuo* to give Diethyl (3-(4-methoxyphenyl)-3-oxopropyl)phosphonate **3p** (30.5 mg, 0.10 mmol, 63%).

Characterisations:

Appearance: Colourless oil. **^{1}H NMR (400 MHz, Chloroform-*d*):** δ ppm 7.94 (d, J = 8.9 Hz, 2H, ArH), 6.92 (d, J = 8.9 Hz, 2H, ArH), 4.19 – 4.03 (m, 4H, 2 x OCH_2), 3.85 (s, 3H, OCH_3), 3.28 – 3.18 (m, 2H, $COCH_2$), 2.24 – 2.11 (m, 2H, PCH_2), 1.31 (t, J = 7.1 Hz, 6H, 2 x CH_2CH_3). **^{13}C{^{1}H} NMR (101 MHz, Chloroform-*d*):** δ ppm 196.1 (d, J = 16.0 Hz, C), 163.8 (C), 130.4 (CH), 129.5 (C), 113.9 (CH), 61.8 (d, J = 6.5 Hz, CH_2), 55.6 (CH_3), 31.4 (d, J = 3.0 Hz, CH_2), 19.9 (d, J = 144.5 Hz, CH_2), 16.5 (d, J = 6.0 Hz, CH_3). ^{31}P NMR (162 MHz, Chloroform-*d*): δ ppm 32.06. IR: ν_{max}/cm^{-1} 3464, 3051, 2982, 2933, 2842, 1674, 1652, 1598, 1575, 1511, 1444, 1419, 1357, 1311, 1248, 1170. TLC: **R*f*** = 0.23 (90:10 hexanes:EtOAc). Data is consistent with the literature.[9]

Methyl 2-(4-methoxybenzoyl)cyclopentane-1-carboxylate (3q)

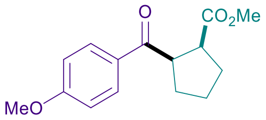

major diastereomer shown

Using Conditions B: General procedure for Conditions B was followed. Methyl cyclopent-1-ene-1-carboxylate **2g** (20.1 mg, 0.16 mmol, 1.0 eq.), 2-(4-methoxyphenyl)-2-oxoacetic acid **1b** (57.7 mg, 0.32 mmol, 2.0 eq.), $(NH_4)_2S_2O_8$ (109.5 mg, 0.48 mmol, 3.0 eq.), γ-terpinene (51.3 μL, 0.32 mmol, 2.0 eq.), and 2,4,6-collidine (42.3 μL, 0.32 mmol, 2.0 eq.) in DMSO (0.4 mL) was irradiated with 450 nm for 16.5 h. The crude was then purified *via* column flash chromatography eluting with 91:9→78:22 hexane:EtOAc. The solvent was then removed *in vacuo* to give methyl 3-(4-methoxybenzoyl)cyclopentane-1-carboxylate **3q** as a 4.7:1 mixture of diastereomers that were separable *via* column chromatography. The major diastereomer was isolated (22.7 mg, 0.09 mmol, 54%) and the minor diastereomer was isolated (7.5 mg, 0.03 mmol, 18%). Combined yield of both diastereomers (30.2 mg, 0.12 mmol, 72%).

Characterisations:

Major diastereomer:

Appearance: Off-white solid. **^{1}H NMR (400 MHz, Chloroform-*d*):** δ ppm 7.91 (d, *J* = 9.0 Hz, 2H, ArH), 6.92 (d, *J* = 9.0 Hz, 2H, ArH), 4.08 (td, *J* = 8.5, 5.5 Hz, 1H, C<u>H</u>COAr), 3.86 (s, 3H, OCH_3), 3.52 (s, 3H, OCH_3), 3.03 (q, *J* = 8.5 Hz, 1H, C<u>H</u>CO_2Me), 2.26 – 2.15 (m, 1H, C<u>H</u>H), 2.14 – 1.90 (m, 3H, CH<u>H</u>, CH_2), 1.90 – 1.80 (m, 1H, C<u>H</u>H), 1.75 – 1.61 (m, 1H, CH<u>H</u>). **^{13}C{^{1}H} NMR (101 MHz, Chloroform-*d*):** δ ppm 199.5 (C), 174.5 (C), 163.5 (C), 130.8 (CH), 129.6 (C), 113.8 (CH), 55.6 (CH), 51.5 (CH), 48.7 (CH_3), 47.8 (CH_3), 30.4 (CH_2), 28.3 (CH_2), 24.3 (CH_2). **IR:** ν_{max}/cm^{-1} 3078, 2958, 2874, 2846, 2361, 1740, 1662, 1601, 1576, 1510, 1459, 1423, 1361, 1319, 1283, 1255, 1226, 1201, 1169, 1143. **TLC:** R_f = 0.20 (80:20 hexanes:EtOAc). **HRMS (ESI-TOF):** *m/z* $[M + H]^+$ calcd for $C_{15}H_{19}O_4$, 263.1278; found, 263.1285. **M.p.:** 56-60 °C. Characterisation of the *syn* diastereomer is supported by literature precedent of analogous compound.[13, 14]

Minor diastereomer:

Appearance: Colourless oil. **^{1}H NMR (400 MHz, Chloroform-*d*):** δ ppm 7.98 (d, *J* = 8.0 Hz, 2H, ArH), 6.94 (d, *J* = 8.0 Hz, 2H, ArH), 4.06 (q, *J* = 7.0 Hz, 1H, C$\underline{H}$COAr), 3.87 (s, 1H, OCH_3) 3.64 (s, 3H, OCH_3), 3.44 (q, *J* = 8.0 Hz, 1H, C$\underline{H}$$CO_2$Me), 2.19 – 2.08 (m, 2H, C$\underline{H}$H, C$\underline{H}$H), 1.96 – 1.86 (m, 1H, CH$\underline{H}$), 1.83 – 1.71 (m, 3H, CH$\underline{H}$, CH_2). **^{13}C{^{1}H} NMR (101 MHz, Chloroform-*d*):** δ ppm 199.9 (C), 176.4 (C), 163.9 (C), 131.3 (CH), 129.8 (C), 114.1 (CH), 55.8 (CH), 52.2 (CH), 49.6 (CH), 46.4 (CH), 32.2 (CH_2), 31.0 (CH_2), 26.2 (CH_2). **IR:** ν_{max}/cm^{-1} 2975, 2928, 2856, 2361, 1736, 1676, 1600, 1577, 1511, 1443, 1420, 1381, 1350, 1296, 1260, 1228, 1168, 1117. **TLC:** R_f = 0.30 (80:20 hexanes:EtOAc). **HRMS (ESI-TOF):** *m*/*z* [M + H]$^+$ calcd for $C_{15}H_{19}O_4$, 263.1278; found, 263.1279. Characterisation of the *anti* diastereomer is supported by literature precedent of analogous compound.[13, 14]

Methyl 2-(4-methoxybenzoyl)cyclohexane-1-carboxylate (3r)

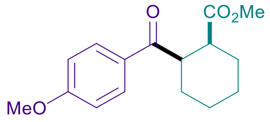

major diastereomer shown

Using Conditions B: General procedure for Conditions B was followed. Methyl cyclohex-1-ene-1-carboxylate **2h** (22.3 mg, 0.16 mmol, 1.0 eq.), 2-(4-methoxyphenyl)-2-oxoacetic acid **1b** (58.0 mg, 0.32 mmol, 2.0 eq.), $(NH_4)_2S_2O_8$ (109.6 mg, 0.48 mmol, 3.0 eq.), γ-terpinene (51.3 μL, 0.32 mmol, 2.0 eq.), and 2,4,6-collidine (42.3 μL, 0.32 mmol, 2.0 eq.) in DMSO (0.4 mL) was irradiated with 450 nm for 16.5 h. The crude was then purified *via* column flash chromatography eluting with 98:12 hexane:EtOAc. The solvent was then removed *in vacuo* to give methyl 3-(4-methoxybenzoyl)cyclohexane-1-carboxylate **3r** as a 5.1:1 mixture of diastereomers that were separable *via* column chromatography. The major diastereomer was isolated (24.3 mg, 0.09 mmol, 55%) and the minor diastereomer was isolated (4.5 mg, 0.02 mmol, 10%). Combined yield of both diastereomers (28.8 mg, 0.10 mmol, 66%).

Characterisations:

Major diastereomer:

Appearance: Colourless oil. **^{1}H NMR (400 MHz, Chloroform-*d*):** δ ppm 7.87 (d, *J* = 9.0 Hz, 2H, ArH), 6.92 (d, *J* = 9.0 Hz, 2H, ArH), 3.88 – 3.83 (m, 1H, C<u>H</u>COAr) 3.85 (s, 3H, OCH_3), 3.61 (s, 3H, OCH_3), 2.69 (dt, *J* = 9.5, 4.5 Hz, 1H, C<u>H</u>CO_2Me), 2.28 – 2.16 (m, 1H, C<u>H</u>H), 2.16 – 2.04 (m, 1H, C<u>H</u>H), 2.00 – 1.88 (m, 1H, CH<u>H</u>), 1.85 – 1.72 (m, 2H, C<u>H</u>H, CH<u>H</u>), 1.52 – 1.28 (m, 3H, CH<u>H</u>, CH_2). **^{13}C{^{1}H} NMR (101 MHz, Chloroform-*d*):** δ ppm 201.0 (C), 174.8 (C), 163.2 (C), 130.6 (CH), 129.6 (C), 113.8 (CH), 55.6 (CH_3), 51.7 (CH_3), 44.1 (CH), 43.0 (CH), 27.9 (CH_2), 25.7 (CH_2), 24.4 (CH_2), 22.8 (CH_2). **IR:** ν_{max}/cm^{-1} 2935, 2855, 2358, 1728, 1671, 1597, 1575, 1510, 1434, 1447, 1419, 1382, 1347, 1305, 1240, 1216, 1165. **TLC:** R_f = 0.28 (80:20 hexanes:EtOAc). **HRMS (ESI-TOF):** *m/z* $[M + H]^+$ calcd for $C_{16}H_{21}O_4$, 277.1434; found, 277.1434. Characterisation of the *syn* diastereomer is supported by literature precedent of analogous compound.[15]

Minor diastereomer:

Appearance: Colourless oil. **^{1}H NMR (400 MHz, Chloroform-*d*):** δ ppm 7.97 (d, *J* = 9.0 Hz, 2H, ArH), 6.94 (d, *J* = 9.0 Hz, 2H, ArH), 3.87 (s, 3H, OCH_3), 3.57 (s, 3H, OCH_3), 3.55 (ddd, *J* = 12.0, 11.0, 3.0 Hz, 1H, C<u>H</u>COAr), 2.92 (ddd, *J* = 12.0, 11.0, 3.5 Hz, 1H, C<u>H</u>CO_2Me), 2.21 – 2.13 (m, 1H, C<u>H</u>H), 2.04 – 1.96 (m, 1H, C<u>H</u>H), 1.90 – 1.80 (m, 2H, 2 x C<u>H</u>H), 1.50 – 1.36 (m, 3H, 3 x CH<u>H</u>), 1.32 – 1.24 (m, 1H, CH<u>H</u>). **^{13}C{^{1}H} NMR (101 MHz, Chloroform-*d*):** δ ppm 201.9 (C), 176.2 (C), 163.8 (C), 131.0 (CH), 129.5 (C), 114.1 (CH), 55.8 (CH_3), 52.0 (CH_3), 46.9 (CH), 45.0 (CH), 30.4 (CH_2), 29.6 (CH_2), 26.0 (CH_2), 25.9 (CH_2). **IR:** ν_{max}/cm^{-1} 2935, 2855, 2358, 1728, 1671, 1597, 1575, 1510, 1447, 1434, 1419, 1382, 1347, 1305, 1240, 1216, 1165, 1125. **TLC:** R_f = 0.30 (hexanes:EtOAc). **HRMS (ESI-TOF):** *m*/*z* $[M + H]^+$ calcd for $C_{16}H_{21}O_4$, 277.1434; found, 277.1443.

The minor diastereomer is assigned as *anti*, based on the multiplicities and *J* values of the CHCO peak. The C<u>H</u>CO_2Me peak at δ 2.92 ppm (H_b) is a ddd with *J* = 12.0, 11.0, 3.5 Hz. The large 12.0 and 11.0 Hz are consistent with $^3J_{axial\text{-}axial}$ and small 3.5 Hz is consistent $^3J_{equatorial\text{-}axial,}$ indicating the *anti* diastereomer (minor conformer with both substituents equatorial).

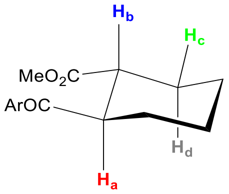

Ar = *p*-OMe-C_6H_4

3-(4-Methoxybenzoyl)cyclohexan-1-one (3s)

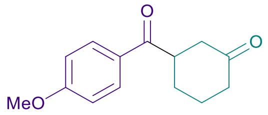

Using Conditions A: General procedure for Conditions A was followed. Cyclohex-2-en-1-one **2i** (10.2 mg, 0.10 mmol, 1.0 eq.), 2-(4-methoxyphenyl)-2-oxoacetic acid **1b** (36.7 mg, 0.20 mmol, 2.0 eq.), $(NH_4)_2S_2O_8$ (68.9 mg, 0.30 mmol, 3.0 eq.), γ-terpinene (32.1 μL, 0.20 mmol, 2.0 eq.), and 2,4,6-collidine **7** (26.5 μL, 0.20 mmol, 2.0 eq.) in DMSO (0.25 mL) was heated to 50 °C for 24 h. The crude was then purified *via* column flash chromatography eluting with 70:30 hexane:EtOAc. The solvent was then removed *in vacuo* to give 13-(4-methoxybenzoyl)cyclohexan-1-one **3s** (6.0 mg, 0.03 mmol, 24%).

Using Conditions B: General procedure for Conditions B was followed. Cyclohex-2-en-1-one **2i** (15.5 mg, 0.16 mmol, 1.0 eq.), 2-(4-methoxyphenyl)-2-oxoacetic acid **1b** (57.8 mg, 0.48 mmol, 2.0 eq.), $(NH_4)_2S_2O_8$ (109.7 mg, 0.48 mmol, 3.0 eq.), γ-terpinene (51.3 μL, 0.36 mmol, 2.0 eq.), and 2,4,6-collidine (42.3 μL, 0.24 mmol, 2.0 eq.) in DMSO (0.8 mL) was irradiated with 450 nm for 24 h. The crude was then purified *via* column flash chromatography eluting with 75:25→65:35 hexane:EtOAc. The solvent was then removed *in vacuo* to give 3-(4-methoxybenzoyl)cyclohexan-1-one **3s** (27.0 mg, 0.12 mmol, 72%).

Characterisations:

Appearance: Colourless oil. **^{1}H NMR (400 MHz, Chloroform-*d*):** δ ppm 7.93 (d, *J* = 9.0 Hz, 2H, ArH), 6.95 (d, *J* = 9.0 Hz, 2H, ArH), 3.87 (s, 3H, OCH_3), 3.81 – 3.72 (m, 1H, C<u>H</u>COAr), 2.71 (dd, *J* = 14.5, 11.0 Hz, 1H, COC<u>H</u>H(CHCOAr)), 2.49 – 2.37 (m, 3H, COCH<u>H</u>(CHCOAr), CH_2), 2.15 – 2.05 (m, 2H, 2 x C<u>H</u>H), 1.92 – 1.75 (m, 2H, 2 x CH<u>H</u>). **^{13}C{^{1}H} NMR (101 MHz, Chloroform-*d*):** δ ppm 210.6 (C), 199.0 (C), 164.0 (C), 130.8 (CH), 128.4 (C), 114.2 (CH), 55.7 (CH_3), 45.0 (CH), 43.5 (CH_2), 41.2 (CH_2), 28.7 (CH_2), 25.0 (CH_2). **IR:** ν_{max}/cm^{-1} 2933, 1699, 1662, 1652, 1600, 1511, 1456, 1419, 1378, 1313, 1258, 1241, 1185, 1173. **TLC:** R_f = 0.28 (64:36 hexanes:EtOAc). **M.p.:** 101-105 °C. All data consistent with the literature.[16]

3-(4-Methoxybenzoyl)-2-methylcyclohexan-1-one (3t)

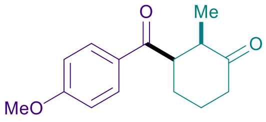

major diastereomer shown

Using Conditions B: General procedure for Conditions B was followed. 2-Methylcyclohex-2-en-1-one **2j** (17.5 mg, 0.16 mmol, 1.0 eq.), 2-(4-methoxyphenyl)-2-oxoacetic acid **1b** (57.8 mg, 0.32 mmol, 2.0 eq.), $(NH_4)_2S_2O_8$ (109.5 mg, 0.48 mmol, 3.0 eq.), γ-terpinene (51.3 µL, 0.32 mmol, 2.0 eq.), and 2,4,6-collidine (42.3 µL, 0.32 mmol, 2.0 eq.) in DMSO (0.4 mL) was irradiated with 450 nm for 16.5 h. The crude was then purified *via* column flash chromatography eluting with 80:20→65:35 hexane:EtOAc. The solvent was then removed *in vacuo* to give 3-(4-methoxybenzoyl)-2-methylcyclohexan-1-one **3t** as a 3.8:1 mixture of diastereomers that were separable *via* column chromatography. The major diastereomer was isolated (22.3 mg, 0.09 mmol, 57%) and the minor diastereomer was isolated (4.1 mg, 0.02 mmol, 10%). Combined yield of both diastereomers (26.4 mg, 0.11 mmol, 67%).

Characterisations:

Major diastereomer:

Appearance: Colourless oil. **^{1}H NMR (400 MHz, Chloroform-*d*):** δ ppm 7.93 (d, J = 9.0 Hz, 2H, ArH), 6.94 (d, J = 9.0 Hz, 2H, ArH), 4.08 (q, J = 4.5 Hz, 1H, CHCOAr), 3.87 (s, 3H, OCH_3), 2.60 – 2.48 (m, 2H, C$\underline{H}CH_3$, C$\underline{H}$H), 2.33 – 2.20 (m, 1H, CH$\underline{H}$), 2.13 – 2.02 (m, 2H, CH_2), 1.91 – 1.76 (m, 2H, CH_2), 1.03 (d, J = 7.0 Hz, 3H, CHC$\underline{H}_3$). **^{13}C{^{1}H} NMR (101 MHz, Chloroform-*d*):** δ ppm) 210.6 (C), 199.1 (C), 163.9 (C), 130.8 (CH), 129.2 (C), 114.1 (CH), 55.7 (CH), 49.6 (CH), 46.4 (CH), 39.7 (CH_2), 27.1 (CH_2), 22.6 (CH_2), 12.8 (CH_3). **IR:** ν_{max}/cm^{-1} 2988, 2933, 2858, 1702, 1652, 1602, 1573, 1511, 1458, 1440, 1422, 1377, 1322, 1264, 1252, 1221, 1200, 1174, 1140. **TLC:** R_f = 0.15 (70:30 hexanes:EtOAc). **HRMS (ESI-TOF):** *m*/*z* [M + H]$^+$ calcd for $C_{15}H_{19}O_3$, 247.1329; found, 247.1330.

Minor diastereomer:

Appearance: Colourless oil. **^{1}H NMR (400 MHz, Chloroform-*d*):** δ ppm 7.95 (d, *J* = 9.0 Hz, 2H, ArH), 6.96 (d, *J* = 9.0 Hz, 2H, ArH), 3.88 (s, 3H, OCH_3), 3.46 (td, *J* = 11.0, 3.5 Hz, 1H, C<u>H</u>COAr), 3.03 – 2.94 (m, 1H, C<u>H</u>Me), 2.53 – 2.38 (m, 2H, CH_2CO), 2.20 – 2.13 (m, 1H, C<u>H</u>H), 2.06 – 1.99 (m, 1H, C<u>H</u>H), 1.92 – 1.77 (m, 2H, 2 x CH<u>H</u>), 0.95 (d, *J* = 6.4 Hz, 3H, CHC<u>H</u>$_3$). **^{13}C{^{1}H} NMR (101 MHz, Chloroform-*d*):** δ ppm 213.0 (C), 199.7 (C), 164.3 (C), 131.0 (CH), 129.8 (C), 114.4 (CH), 55.9 (CH_3), 52.5 (CH), 46.7 (CH), 41.7 (CH_2), 30.4 (CH_2), 26.7 (CH_2), 13.0 (CH_3). **IR:** ν_{max}/cm^{-1} 2933, 2867, 2357, 1708, 1667, 1596, 1575, 1510, 1458, 1420, 1365, 1346, 1308, 1258, 1237, 1203, 1237, 1203, 1169, 1117, 1078. **TLC:** R_f = 0.30 (70:30 hexane:EtOAc). **HRMS (ESI-TOF):** *m/z* $[M + H]^+$ calcd for $C_{15}H_{19}O_3$, 247.1329; found, 247.1320.

Assignment of diastereomers by coupling constants:

The major diastereomer is assigned as *syn*, based on the multiplicities and *J* values of the CH peaks. For example, the CH peak at δ 4.08 ppm is a q with *J* = 4.5 Hz, which is consistent with $^3J_{axial\text{-}equatorial}$ or $^3J_{equatorial\text{-}equatorial}$, indicating the *syn* isomer.

The minor diastereomer is assigned as *anti*, based on the multiplicities and *J* values of the CHH peaks. For example, the C<u>H</u>COAr peak at δ 3.46 ppm (H_b) is a td with *J* = 11.0, 3.5 Hz. The large 11.0 Hz and small 3.5 Hz are consistent with $^3J_{axial\text{-}axial}$ and $^3J_{equatorial\text{-}axial,}$ indicating the *anti* diastereomer (major conformer of *anti* diastereomer with both substituents equatorial).

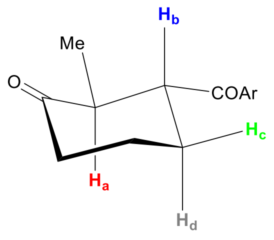

Ar = *p*-OMe-C_6H_4

3-(4-Methoxybenzoyl)cyclopentan-1-one (3u)

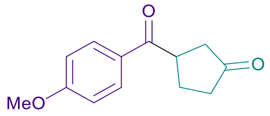

Using Conditions A: General procedure for Conditions A was followed. Cyclopent-2-en-1-one **2k** (13.1 mg, 0.16 mmol, 1.0 eq.), 2-(4-methoxyphenyl)-2-oxoacetic acid **1b** (57.8 mg, 0.32 mmol, 2.0 eq.), $(NH_4)_2S_2O_8$ (109.5 mg, 0.48 mmol, 3.0 eq.), γ-terpinene (51.3 µL, 0.32 mmol, 2.0 eq.), and 2,4,6-collidine **7** (42.3 µL, 0.32 mmol, 2.0 eq.) in DMSO (0.4 mL) was heated to 50 °C for 24 h. The crude was then purified *via* column flash chromatography eluting with 70:30 hexane:EtOAc. The solvent was then removed *in vacuo* to give 3-(4-methoxybenzoyl)cyclopentan-1-one **3u** (10.2 mg, 0.08 mmol, 48%).

Using Conditions B: General procedure for Conditions B was followed. Cyclopent-2-en-1-one **2k** (13.3 mg, 0.16 mmol, 1.0 eq.), 2-(4-methoxyphenyl)-2-oxoacetic acid **1b** (57.8 mg, 0.48 mmol, 2.0 eq.), $(NH_4)_2S_2O_8$ (109.8 mg, 0.48 mmol, 3.0 eq.), γ-terpinene (51.3 µL, 0.36 mmol, 2.0 eq.), and 2,4,6-collidine (42.3 µL, 0.24 mmol, 2.0 eq.) in DMSO (0.8 mL) was irradiated with 450 nm for 24 h. The crude was then purified *via* column flash chromatography eluting with 75:25→70:30 hexane:EtOAc. The solvent was then removed *in vacuo* to give 3-(4-methoxybenzoyl)cyclopentan-1-one **3u** (16.8 mg, 0.08 mmol, 48%).

Characterisations:

Appearance: Colourless oil. **^{1}H NMR (400 MHz, Chloroform-*d*):** δ ppm 7.98 (d, *J* = 9.0 Hz, 2H, ArH), 6.97 (d, *J* = 9.0 Hz, 2H, ArH), 4.11 – 4.04 (m, 1H, C<u>H</u>COAr), 3.89 (s, 3H, OCH_3), 2.75 – 2.65 (m, 1H, C<u>H</u>H(CHCOAr)), 2.47 – 2.11 (m, 5H, CH<u>H</u>(CHCOAr), 2 x C<u>H</u>H, 2 x CH<u>H</u>). **^{13}C{^{1}H} NMR (101 MHz, Chloroform-*d*):** δ ppm 217.2 (C), 198.8 (C), 164.0 (C), 130.9 (CH), 128.8 (C), 114.2 (CH), 55.7 (CH_3), 42.8 (CH), 41.3 (CH_2), 37.5 (CH_2), 27.3 (CH_2). **IR:** ν_{max}/cm^{-1} 3063, 3053, 2966, 2840, 1738, 1665, 1596, 1575, 1510, 1458, 1421, 1365, 1311, 1256, 1225, 1172, 1160. **TLC:** R_f = 0.28 (66:34 hexane;EtOAc). All data consistent the literature.[17]

4-Isopropyl-3-(4-methoxybenzoyl)cyclohexan-1-one (3v)

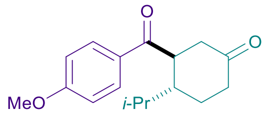

Using Conditions B: General procedure for Conditions B was followed. (±)-Cryptone **2l** (22.1 mg, 0.16 mmol, 1.0 eq.), 2-(4-methoxyphenyl)-2-oxoacetic acid **1b** (57.8 mg, 0.32 mmol, 2.0 eq.), $(NH_4)_2S_2O_8$ (109.6 mg, 0.48 mmol, 3.0 eq.), γ-terpinene (51.3 µL, 0.32 mmol, 2.0 eq.), and 2,4,6-collidine (42.3 µL, 0.32 mmol, 2.0 eq.) in DMSO (0.8 mL) was irradiated with 450 nm for 24 h. The crude was then purified *via* column flash chromatography eluting with 80:20→60:30 hexane:EtOAc. The solvent was then removed *in vacuo* to give 4-isopropyl-3-(4-methoxybenzoyl)cyclohexan-1-one **3v** as the *anti* diastereomer (15.9 mg, 0.06 mmol, 36%). The *syn* diastereomer was not observed by ^{1}H NMR analysis of the crude.

Characterisations:

Appearance: Yellow solid. **^{1}H NMR (400 MHz, Chloroform-*d*):** δ ppm 7.95 (d, *J* = 9.0 Hz, 2H, ArH), 6.95 (d, *J* = 9.0 Hz, 2H, ArH), 3.87 (s, 3H, OCH_3), 3.83 (ddd, *J* = 11.0, 9.0, 4.5 Hz, 1H, CHCOAr), 2.57 (dd, *J* = 14.5, 11.0 Hz, 1H, COCHH(CHCOAr)), 2.48 – 2.38 (m, 3H, COCHH(CHCOAr), CH_2), 2.24 – 2.15 (m, 1H, CH($CHMe_2$)), 2.06 – 1.97 (m, 1H, CHH), 1.76 (pd, *J* = 7.0, 4.0 Hz, 1H, $CHMe_2$), 1.69 – 1.55 (m, 1H, CHH), 0.99 (d, *J* = 7.0 Hz, 3H, $CHCH_3CH_3$), 0.78 (d, *J* = 7.0 Hz, 3H, $CHCH_3CH_3$). **^{13}C{^{1}H} NMR (101 MHz, Chloroform-*d*):** δ ppm 210.5 (C), 200.4 (C), 164.1 (C), 130.8 (CH), 129.4 (C), 114.2 (CH), 55.7 (CH_3), 46.8 (CH), 43.7 (CH), 43.6 (CH_2), 40.2 (CH_2), 28.3 (CH), 24.3 (CH_2), 21.7 (CH_3), 17.3 (CH_3). **IR:** ν_{max}/cm^{-1} 2950, 2894, 2873, 2838, 2358, 1717, 1669, 1604, 1573, 1510, 1463, 1429, 1419, 1390, 1373, 1366, 1282, 1274, 1260, 1173. **TLC:** R_f = 0.47 (70:30 hexanes: EtOAc). **HRMS (ESI-TOF):** *m/z* $[M + H]^+$ calcd for $C_{17}H_{23}O_3$, 275.1642; found, 275.1645. **M.p.:** 70-74 °C.

Assignment of diastereomer by coupling constants:

The major diastereomer is assigned as *anti*, based on the multiplicities and *J* values of the CHH peaks. For example, the CHH peak at δ 2.57 ppm is a dd with *J* = 14.5, 11.0 Hz. The large *J* values are consistent with $^3J_{axial\text{-}axial}$ and ^2J (H_a), indicating the *anti* diastereomer (major conformer with both substituents equatorial).

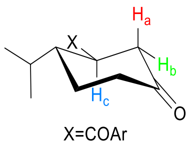

2-(2-(4-Methoxyphenyl)-2-oxo-1-phenylethylidene)malononitrile (8)

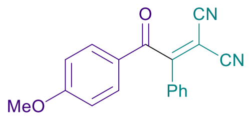

Using Conditions B: General procedure for Conditions B was followed. 2-benzylidenemalononitrile **2m** (24.8 mg, 0.16 mmol, 1.0 eq.), 2-(4-methoxyphenyl)-2-oxoacetic acid **1b** (57.7 mg, 0.32 mmol, 2.0 eq.), $(NH_4)_2S_2O_8$ (109.8 mg, 0.48 mmol, 3.0 eq.), γ-terpinene (51.3 μL, 0.32 mmol, 2.0 eq.), and 2,4,6-collidine (42.3 μL, 0.32 mmol, 2.0 eq.) in DMSO (0.4 mL) was irradiated with 450 nm for 5 h. The crude was then purified *via* column flash chromatography eluting with 90:10→70:30 hexane:EtOAc. The solvent was then removed *in vacuo* to give 2-(2-(4-methoxyphenyl)-2-oxo-1-phenylethylidene)malononitrile **8** (37.3 mg, 0.13 mmol, 80%).

Characterisations:

Appearance: Yellow solid. **^{1}H NMR (400 MHz, Chloroform-*d*):** δ ppm 7.86 (d, J = 9.0 Hz, 2H, ArH), 7.78 – 7.73 (m, 2H, ArH), 7.59 (t, J = 7.5 Hz, 1H, ArH), 7.51 (t, J = 7.5 Hz, 2H, ArH), 6.98 (d, J = 9.0 Hz, 2H, ArH), 3.88 (s, 3H, OCH_3). **^{13}C{^{1}H} NMR (101 MHz, Chloroform-*d*):** δ ppm 189.2 (C), 171.9 (C), 165.8 (C), 133.9 (CH), 132.7 (CH), 130.8 (C), 129.8 (CH), 128.7 (CH), 126.4 (C), 115.0 (CH), 112.2 (C), 111.6 (C), 83.6 (C), 55.9 (CH_3). **IR:** ν_{max}/cm^{-1} 3104, 2973, 2845, 2358, 2225, 1659. 1652, 1594, 1589, 1471, 1460, 1456, 1443, 1426, 1270, 1241, 1175, 1162, 1122, 1015. **TLC:** R_f = 0.28 (80:20 hexanes: EtOAc). **HRMS (ESI-TOF):** *m*/*z* $[M + H]^+$ calcd for $C_{18}H_{13}N_2O_2$, 289.0972; found, 289.0975. **M.p.:** 91-94 °C.

Compound **8** was also isolated in 78% yield using Conditions B in the absence of γ-terpinene.

Ethyl 4-(4-methoxybenzoyl)-2-oxo-2H-chromene-3-carboxylate (9)

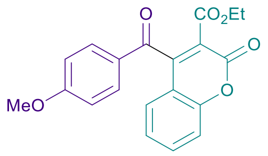

Using Conditions B: General procedure for Conditions B was followed. Ethyl 2-oxo-2H-chromene-3-carboxylate **2n** (26.2 mg, 0.12 mmol, 1.0 eq.), 2-(4-methoxyphenyl)-2-oxoacetic acid **1b** (43.4 mg, 0.24 mmol, 2.0 eq.), $(NH_4)_2S_2O_8$ (82.0 mg, 0.36 mmol, 3.0 eq.) and 2,4,6-collidine (31.8 µL, 0.24 mmol, 2.0 eq.) in DMSO (0.3 mL) was irradiated with 450 nm for 5 h. The crude was then purified *via* column flash chromatography eluting with 78:22→75:25 hexane:EtOAc. The solvent was then removed *in vacuo* to give ethyl 4-(4-methoxybenzoyl)-2-oxo-2H-chromene-3-carboxylate **9** (31.4 mg, 0.09 mmol, 74%).

Characterisations:

Appearance: Colourless oil. **^{1}H NMR (400 MHz, Chloroform-*d*):** δ ppm 7.87 (d, *J* = 9.0 Hz, 2 H, ArH), 7.61 (dd, *J* = 7.5, 1.5 Hz, 1H, ArH), 7.43 – 7.38 (m, 1H, ArH), 7.30 – 7.25 (m, 1H, ArH), 7.23 – 17 (m, 1H, ArH), 6.96 (d, *J* = 9.0 Hz, 2H, ArH), 4.11 (q, *J* = 7.0 Hz, 2H, OCH_2), 3.88 (s, 3H, OCH_3), 1.06 (t, *J* = 7.0 Hz, 3H, $OCH_2C\underline{H}_3$). **^{13}C{^{1}H} NMR (101 MHz, Chloroform-*d*):** δ ppm 190.5 (C), 165.0 (C), 162.5 (C), 156.8 (C), 155.2 (C), 154.4 (C), 134.4 (CH plus one overlapping C), 131.8 (CH), 128.4 (C), 127.9 (CH), 125.2 (CH), 117.4 (CH), 116.7 (C), 114.6 (CH), 62.4 (CH_2), 55.8 (CH_3), 13.7 (CH_3). **IR:** ν_{max}/cm^{-1} 3075, 2982, 2842, 2358, 1750, 1721, 1664, 1594, 1573, 1511, 1453, 1423, 1369, 1302, 1259, 1246, 1165. **TLC:** R_f = 0.26 (72:28 hexanes: EtOAc). **HRMS (ESI-TOF):** *m*/*z* $[M + H]^+$ calcd for $C_{20}H_{17}O_6$, 353.1020; found, 353.1022.

4-(4-Methoxyphenyl)-3-methyl-4-oxobutanoic acid (10)

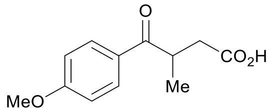

Procedure: Adapting from the literature,[18] to a 4 mL vial equipped with a stirrer bar was added diethyl 2-(1-(4-methoxyphenyl)-1-oxopropan-2-yl)malonate **3d** (58.1 mg, 0.18 mmol, 1 equiv.) followed 6 M HCl (0.6 mL). The vial was then sealed tight and heated to 115 °C with constant stirring for 16 h. The reaction mixture was then cooled to rt and diluted with EtOAc (20 mL), and the resulting solution was washed with H_2O (20 ml). The layers were separated and the aq. layer with extracted with more EtOAc (2 x 20 mL) and the combined organic layers were washed with brine (20 mL), dried over $MgSO_4$, and concentrated *in vacuo* to give crude as a brown oil. The crude was then purified *via* column flash chromatography eluting with 75:23:2 hexane:EtOAc:AcOH. The solvent was then removed *in vacuo* to give 4-(4-methoxyphenyl)-3-methyl-4-oxobutanoic acid **10** (36.1 mg, 0.16 mmol, 90%).

Characterisations:

Appearance: Colourless oil. **^{1}H NMR (400 MHz, Chloroform-*d*):** δ ppm 7.95 (d, *J* = 9.0 Hz, 2H, ArH), 6.93 (d, *J* = 9.0 Hz, 2H, ArH), 3.93 – 3.78 (m, 1H, CHMe), 3.86 (s, 3H, OCH_3), 2.96 (dd, *J* = 17.0, 8.0 Hz, 1H, C<u>H</u>H), 2.46 (dd, *J* = 17.0, 5.5 Hz, 1H, CH<u>H</u>), 1.21 (d, *J* = 7.0 Hz, 2H, CHC<u>H</u>$_3$). **^{13}C{^{1}H} NMR (101 MHz, Chloroform-*d*):** δ ppm 201.2 (C), 178.2 (C), 163.8 (C), 130.9 (CH), 128.7 (C), 114.0 (CH), 55.6 (CH_3), 37.3 (CH_2), 36.8 (CH), 18.2 (CH_3). **IR:** ν_{max}/cm^{-1} 2970 (broad), 2937, 2840, 1706, 1671, 1596, 1574, 1510, 1459, 1419, 1310, 1243, 1168, 1026. **TLC:** R_f = 0.20 (75:23:2 hexane:EtOAc:AcOH. All data consistent with the literature.[18]

6-(4-Methoxyphenyl)-5-methyl-2-phenyl-4,5-dihydropyridazin-3(2H)-one (11)

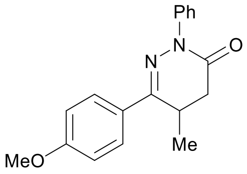

Procedure: Adapting from the literature,[19] to a to a 4 mL vial equipped with a stirrer bar was added diethyl 2-(1-(4-methoxyphenyl)-1-oxopropan-2-yl)malonate **3d** (58.3 mg, 0.18 mmol, 1 equiv.) followed 6 M HCl (0.6 mL). The vial was then sealed tight and heated 115 °C with constant stirring for 20.5 h. The reaction mixture was then cooled to rt and diluted with EtOAc (20 mL), and the resulting solution was washed with H_2O (20 ml). The layers were separated, the aq. layer extracted with more EtOAc (2 x 10 mL) and the combined organic layers washed with brine (20 mL). The organic layer was dried over $MgSO_4$ and concentrated *in vacuo* to give a brown oil. The oil was then transferred to a 4 mL vial and dissolved in *i*-PrOH (0.6 mL). The solution was then charged with phenylhydrazine (53.2 µL, 0.54 mmol, 3 equiv.). The vial was sealed tight and heated to 100 °C with constant stirring for 17 h. The mixture was then diluted with EtOAc (20 mL) and the solution was washed with brine (20 mL) and the layers separated. The aq. layer was extracted with more EtOAc (2 x 10 mL) and the combine organic layers were dried over $MgSO_4$ and concentrated *in vacuo* to give an orange oil. The crude oil was purified *via* column flash chromatography eluting with 70:30 petroleum ether (40-60 °C):EtOAc. Any impure fractions were dissolved in CH_2Cl_2 (20 mL) and the solution was washed with sat. aq. $NaHCO_3$ (30 mL) and the layers separated. The aq. layer was washed with more CH_2Cl_2 (2 x 10 mL) and the combined organic layers were dried over $MgSO_4$ and concentrated *in vacuo* to give a red oil. The oil was re-purified *via* column flash chromatography eluting with 70:30 petroleum ether (40-60 °C):EtOAc to give 6-(4-methoxyphenyl)-5-methyl-2-phenyl-4,5-dihydropyridazin-3(2H)-one **9** (30.6 mg, 0.10 mmol, 57%).

Characterisations:

Appearance: Brown oil. **^{1}H NMR (400 MHz, Chloroform-*d*):** δ ppm 7.79 (d, *J* = 8.9 Hz, 2H, ArH), 7.65 – 7.57 (m, 2H, ArH), 7.41 (t, *J* = 7.9 Hz, 2H, ArH), 7.30 – 7.22 (m, 1H, ArH), 6.93 (d, *J* = 8.9 Hz, 2H, ArH), 3.84 (s, 3H, OCH_3), 3.43 – 3.34 (m, 1H, C$\underline{H}$$CH_3$), 2.87 (dd, *J* = 16.5, 6.5 Hz, 1H, C$\underline{H}$HCO), 2.63 (dd, *J* = 16.5, 1.6 Hz, 1H, CH$\underline{H}$CO), 1.31 (d, *J* = 7.3 Hz, 3H, CHC$\underline{H}_3$). **^{13}C{^{1}H} NMR (101 MHz, Chloroform-*d*):** δ ppm 164.8 (C), 161.3 (C), 155.1 (C),

141.3 (C), 128.6 (CH), 127.8 (CH), 127.0 (C), 126.6 (CH), 124.9 (CH), 114.2 (CH), 55.5 (CH_3), 35.6 (CH_2), 28.4 (CH), 16.5 (CH_3). **IR:** ν_{max}/cm^{-1} 3065, 2965, 2930, 2837, 1678, 1675, 1595, 1568, 1514, 1494, 1456, 1331, 1247, 1172, 1134. **TLC:** R_f = 0.29 (64:36 hexanes:EtOAc). **HRMS (ESI-TOF):** *m*/*z* $[M + H]^+$ calcd for $C_{18}H_{19}N_2O_2$, 295.1441; found, 295.1435.

10. References

(1) Gottlieb, H. E.; Kotlyar, V.; Nudelman, A. NMR Chemical Shifts of Common Laboratory Solvents as Trace Impurities. *J. Org. Chem.* **1997**, *62* (21), 7512-7515.
(2) MacCarthy, P. Simplified experimental route for obtaining Job's curves. *Anal. Chem.* **1978**, *50* (14), 2165-2165.
(3) Pistritto, V. A.; Liu, S.; Nicewicz, D. A. Mechanistic Investigations into Amination of Unactivated Arenes via Cation Radical Accelerated Nucleophilic Aromatic Substitution. *J. Am. Chem. Soc.* **2022**, *144* (33), 15118-15131.
(4) Demas, J. N.; Bowman, W. D.; Zalewski, E. F.; Velapoldi, R. A. Determination of the quantum yield of the ferrioxalate actinometer with electrically calibrated radiometers. *J. Phys. Chem.* **1981**, *85* (19), 2766-2771.
(5) Westwood, M. T.; Lamb, C. J. C.; Sutherland, D. R.; Lee, A.-L. Metal-, Photocatalyst-, and Light-Free Direct C–H Acylation and Carbamoylation of Heterocycles. *Org. Lett.* **2019**, *21* (17), 7119-7123.
(6) Chen, K.; Ishihara, Y.; Galán, M. M.; Baran, P. S. Total synthesis of eudesmane terpenes: cyclase phase. *Tetrahedron* **2010**, *66* (26), 4738-4744.
(7) Wang, G.-Z.; Shang, R.; Cheng, W.-M.; Fu, Y. Decarboxylative 1,4-Addition of α-Oxocarboxylic Acids with Michael Acceptors Enabled by Photoredox Catalysis. *Org. Lett.* **2015**, *17* (19), 4830-4833.
(8) Zhang, M.; Xie, J.; Zhu, C. A general deoxygenation approach for synthesis of ketones from aromatic carboxylic acids and alkenes. *Nat. Commun.* **2018**, *9* (1).
(9) De Pedro Beato, E.; Mazzarella, D.; Balletti, M.; Melchiorre, P. Photochemical generation of acyl and carbamoyl radicals using a nucleophilic organic catalyst: applications and mechanism thereof. *Chem. Sci.* **2020**, *11* (24), 6312-6324.
(10) Burpitt, B. E.; Crawford, L. P.; Davies, B. J.; Mistry, J.; Mitchell, M. B.; Pancholi, K. D.; Coates, W. J. 6-(substituted phenyl)-5-methyl-4,5-dihydro-pyridazin-3(2H)-ones of medicinal interest. The synthesis of SK&F 94836 and SK&F 95654. *J. Heterocycl. Chem.* **1988**, *25* (6), 1689-1695.
(11) Papadopoulos, G. N.; Voutyritsa, E.; Kaplaneris, N.; Kokotos, C. G. Green Photo-Organocatalytic C−H Activation of Aldehydes: Selective Hydroacylation of Electron-Deficient Alkenes. *Chem. Eur. J.* **2018**, *24* (7), 1726-1731.
(12) Vellakkaran, M.; Andappan, M. M. S.; Nagaiah, K.; Nanubolu, J. B. Direct Synthesis of γ-Keto Sulfones from Allylic Alcohols: One-Pot Palladium(II)-Catalyzed Generation of Enones Followed by Water-Mediated 1,4-Addition of Organosulfinates. *Eur. J. Med. Chem.* **2016**, *2016* (21), 3575-3583.
(13) Asaoka, M.; Kosaka, A.; Tanaka, M.; Ueda, T.; Houkawa, T.; Takei, H. Chelation controlled cis-selective acylation of 2-(alkoxycarbonyl)cyclopentylzinc iodides. *J. Chem. Soc., Perkin Trans. 1* **1997**, (20), 2949-2950.
(14) Ding, Z.; Liu, Z.; Wang, Z.; Yu, T.; Xu, M.; Wen, J.; Yang, K.; Zhang, H.; Xu, L.; Li, P. Catalysis with Diboron(4)/Pyridine: Application to the Broad-Scope [3 + 2] Cycloaddition of Cyclopropanes and Alkenes. *J. Am. Chem. Soc.* **2022**, *144* (19), 8870-8882.
(15) Bercot, E. A.; Rovis, T. Highly Efficient Nickel-Catalyzed Cross-Coupling of Succinic and Glutaric Anhydrides with Organozinc Reagents. *J. Am. Chem. Soc.* **2005**, *127* (1), 247-254.
(16) Pálvölgyi, Á. M.; Ehrschwendtner, F.; Schnürch, M.; Bica-Schröder, K. Photocatalyst-free hydroacylations of electron-poor alkenes and enones under visible-light irradiation. *Org. Biomol. Chem.* **2022**, *20* (36), 7245-7249.
(17) Zhou, J.; Wang, Z.; Shen, Z.; Liu, X. Decarboxylative 1,4-Addition of α-Oxocarboxylic Acids with Michael Acceptors Enabled by Direct Excitation of Flavin-Dependent “Ene”-Reductases. *ACS Sustainable Chem. Eng.* **2023**, *11* (10), 4064-4072.
(18) Van der Mey, M.; Bommelé, K. M.; Boss, H.; Hatzelmann, A.; Van Slingerland, M.; Sterk, G. J.; Timmerman, H. Synthesis and Structure−Activity Relationships of cis-Tetrahydrophthalazinone/Pyridazinone Hybrids: A Novel Series of Potent Dual PDE3/PDE4 Inhibitory Agents. *J. Med. Chem.* **2003**, *46* (10), 2008-2016.
(19) Zheng, S.; Zhang, T.; Maekawa, H. Magnesium-Promoted Reductive Carboxylation of Aryl Vinyl Ketones: Synthesis of γ-Keto Carboxylic Acids. *J. Org. Chem.* **2022**, *87* (11), 7342-7349.

11. Appendix

Compound 3a – ¹H NMR (400 MHz, Chloroform-*d*):

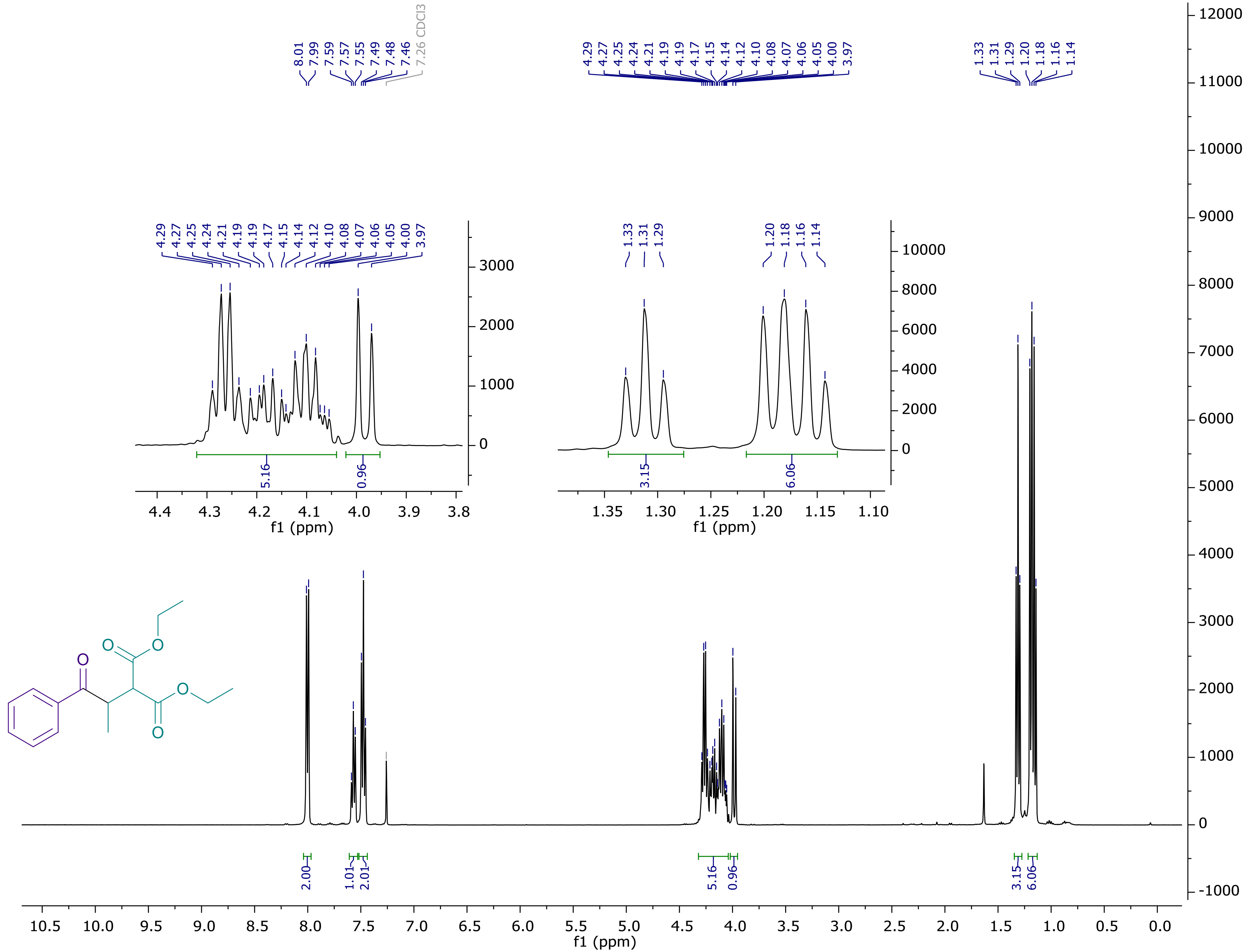

Compound 3a – $^{13}C\{^{1}H\}$ NMR (101 MHz, Chloroform-*d*):

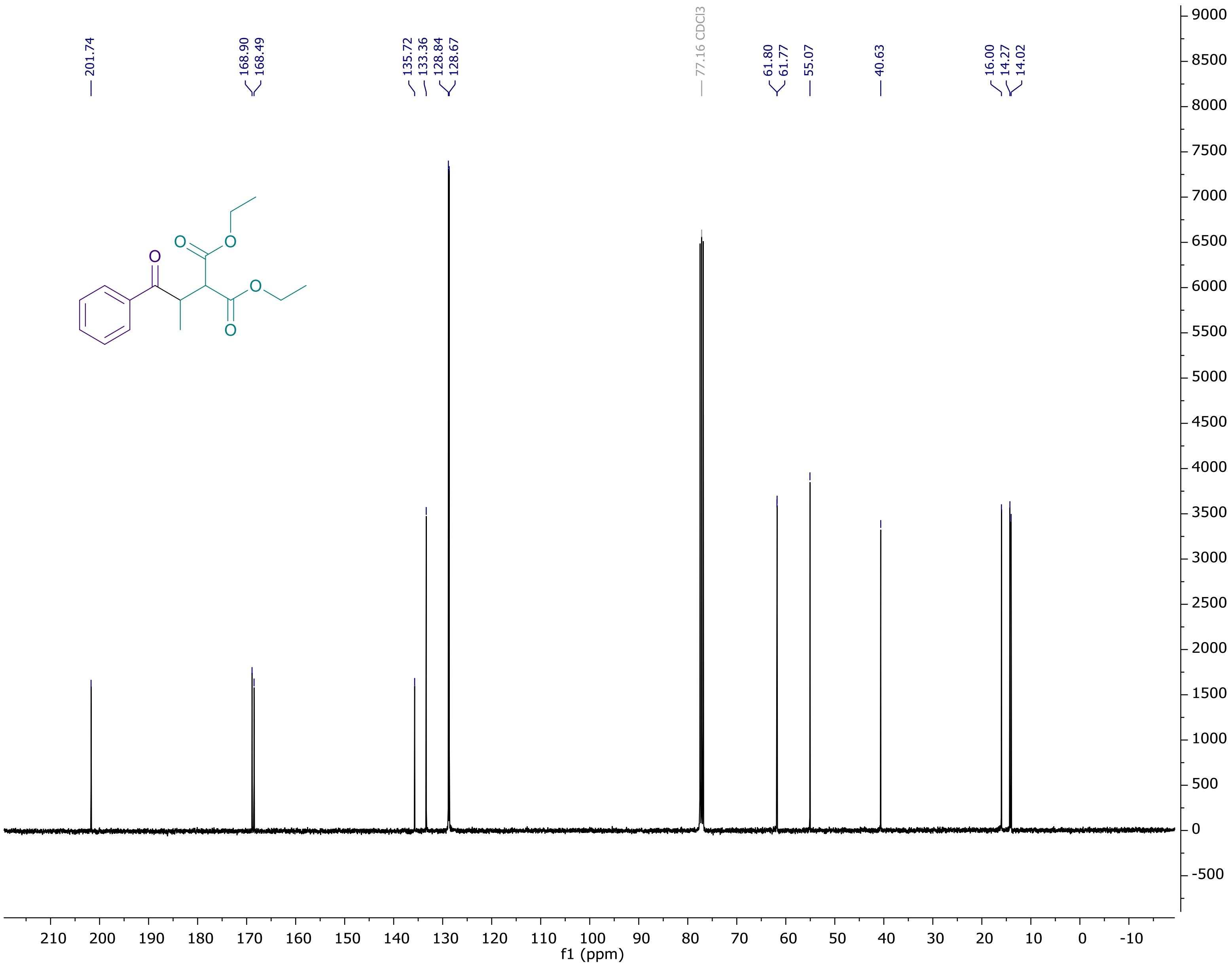

Compound 3b – ^{1}H NMR (400 MHz, Chloroform-*d*):

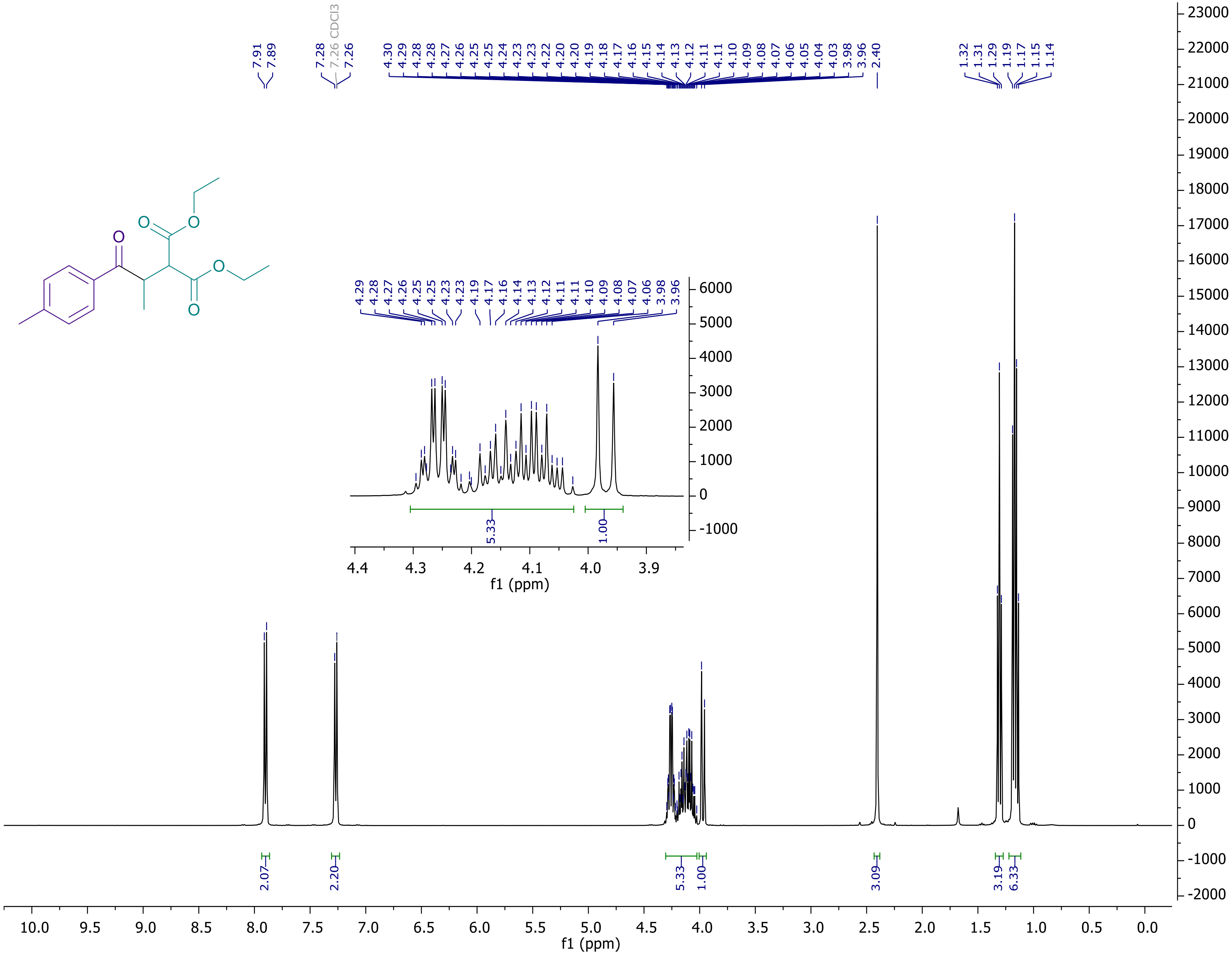

Compound 3b – $^{13}C\{^{1}H\}$ NMR (101 MHz, Chloroform-*d*):

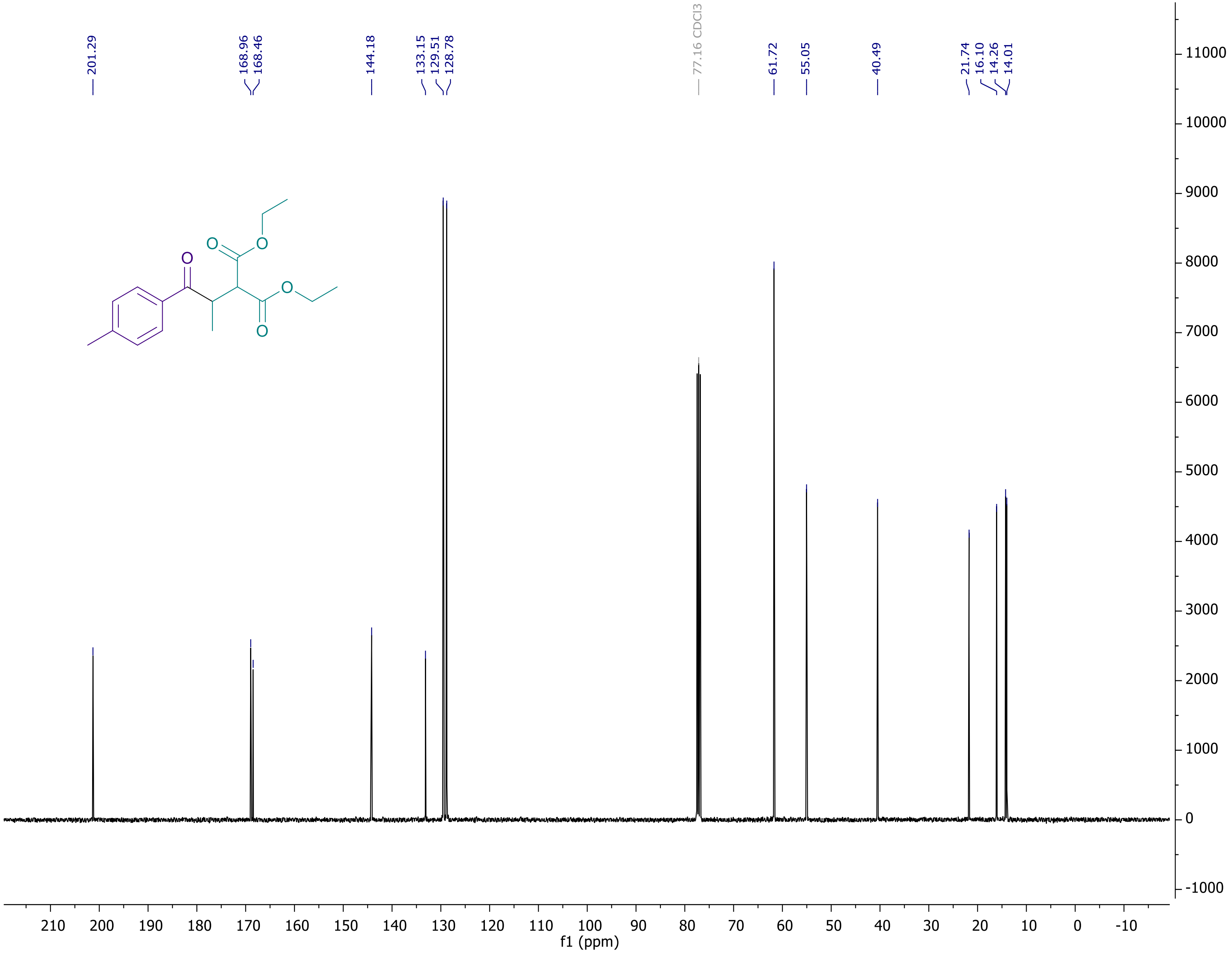

Compound 3c – ^{1}H NMR (400 MHz, Chloroform-*d*):

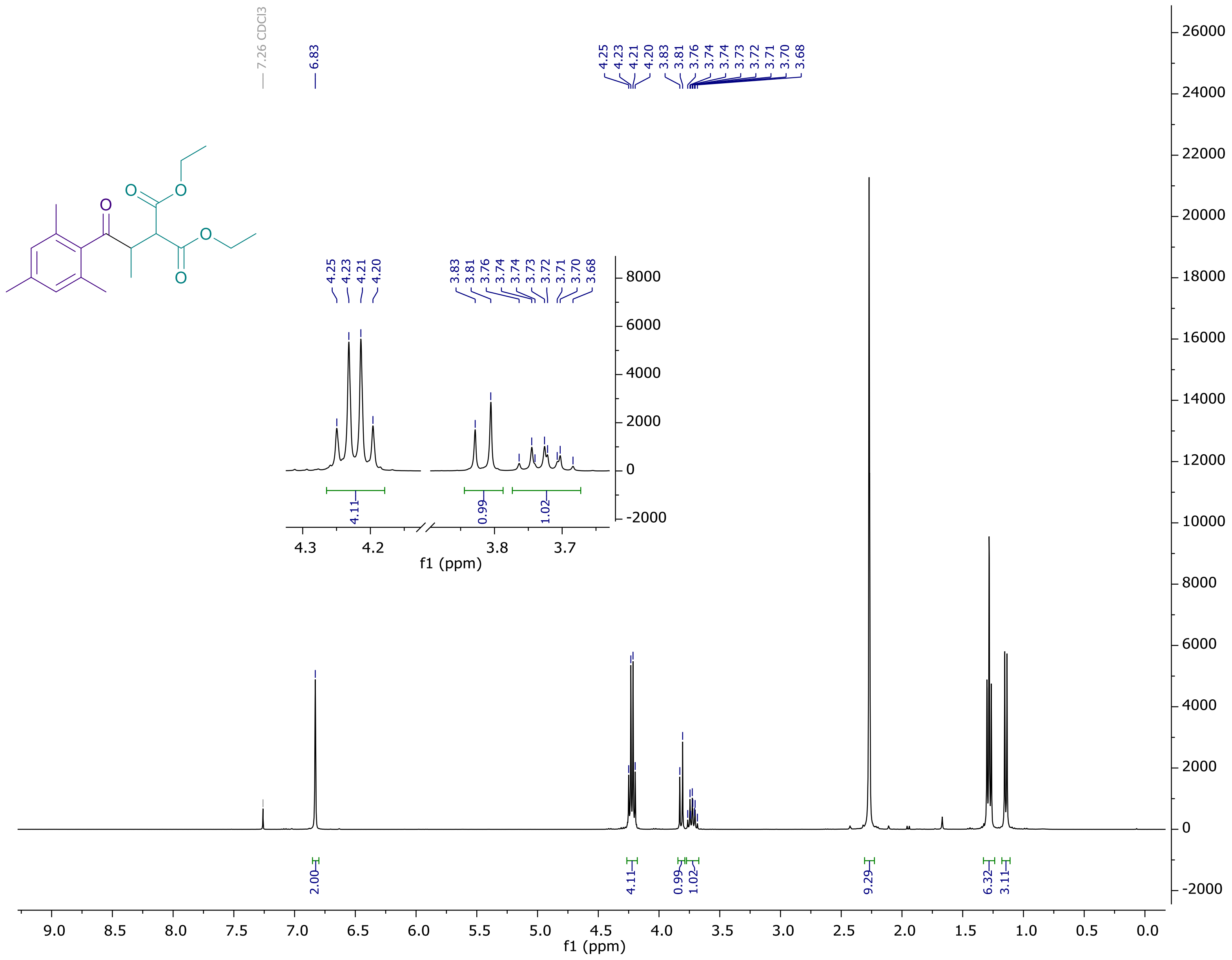

Compound 3c – $^{13}C\{^{1}H\}$ NMR (101 MHz, Chloroform-*d*):

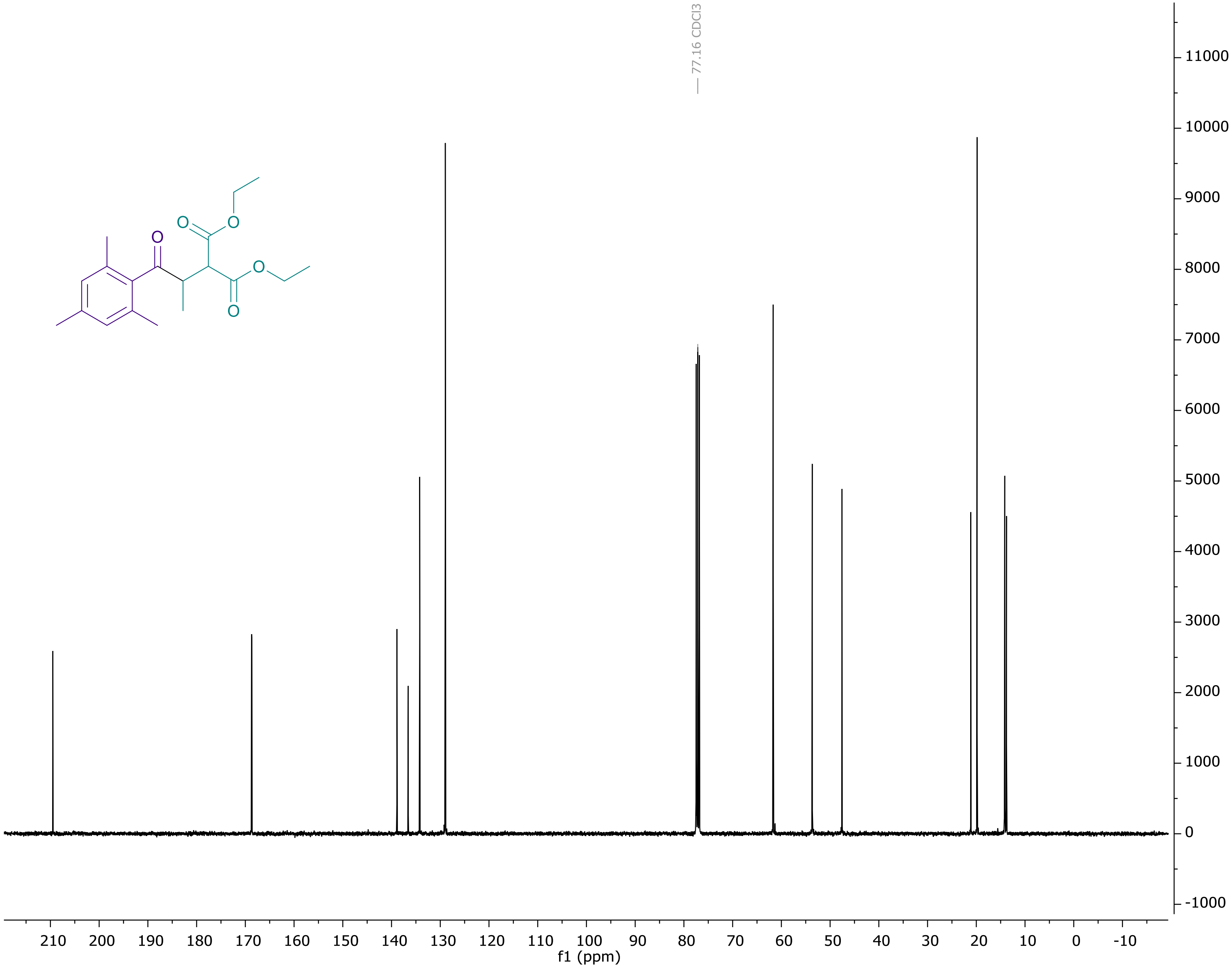

Compound 3d – ^{1}H NMR (400 MHz, Chloroform-*d*):

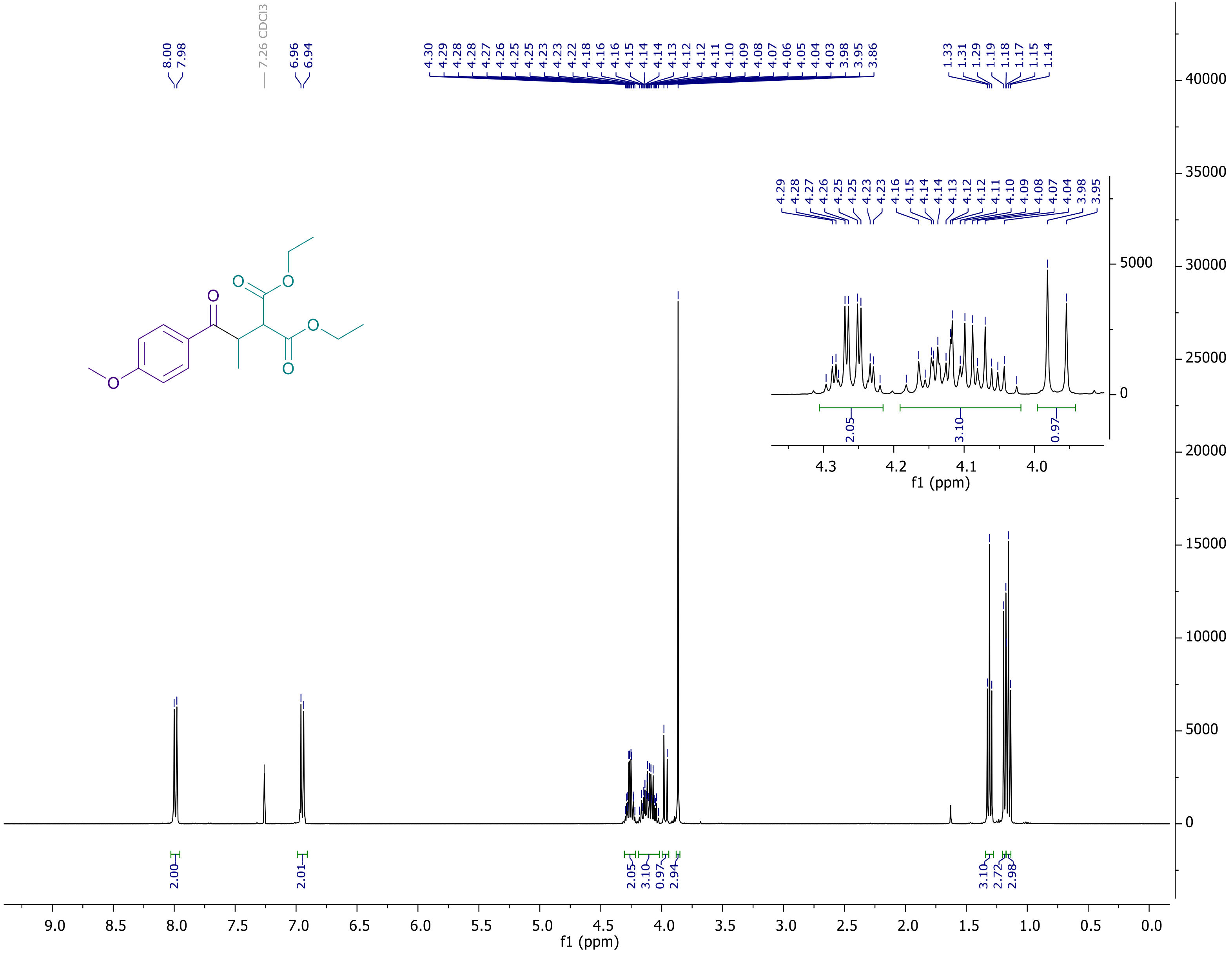

Compound 3d – $^{13}C\{^{1}H\}$ NMR (101 MHz, Chloroform-*d*):

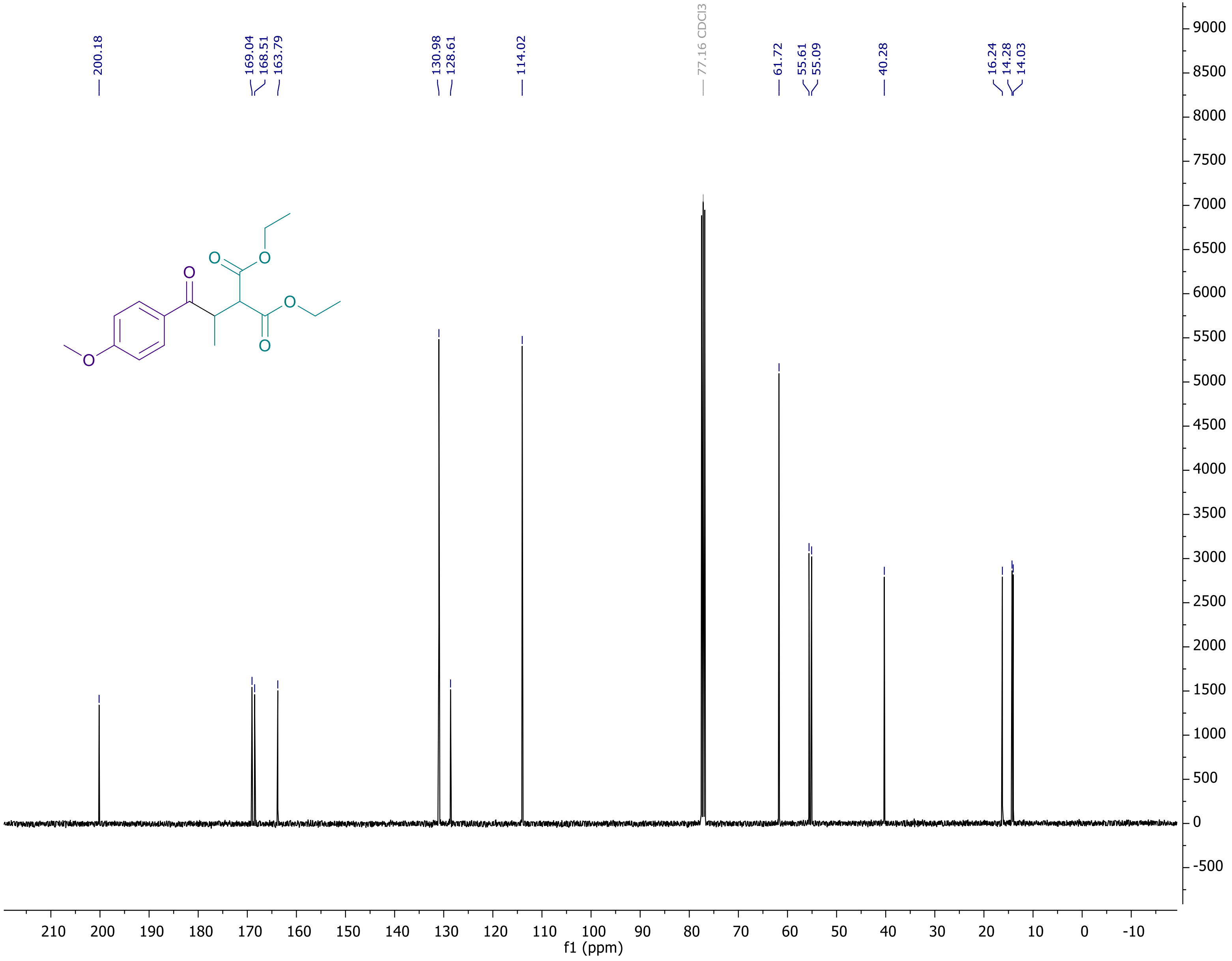

Compound 3e – ^{1}H NMR (400 MHz, Chloroform-d):

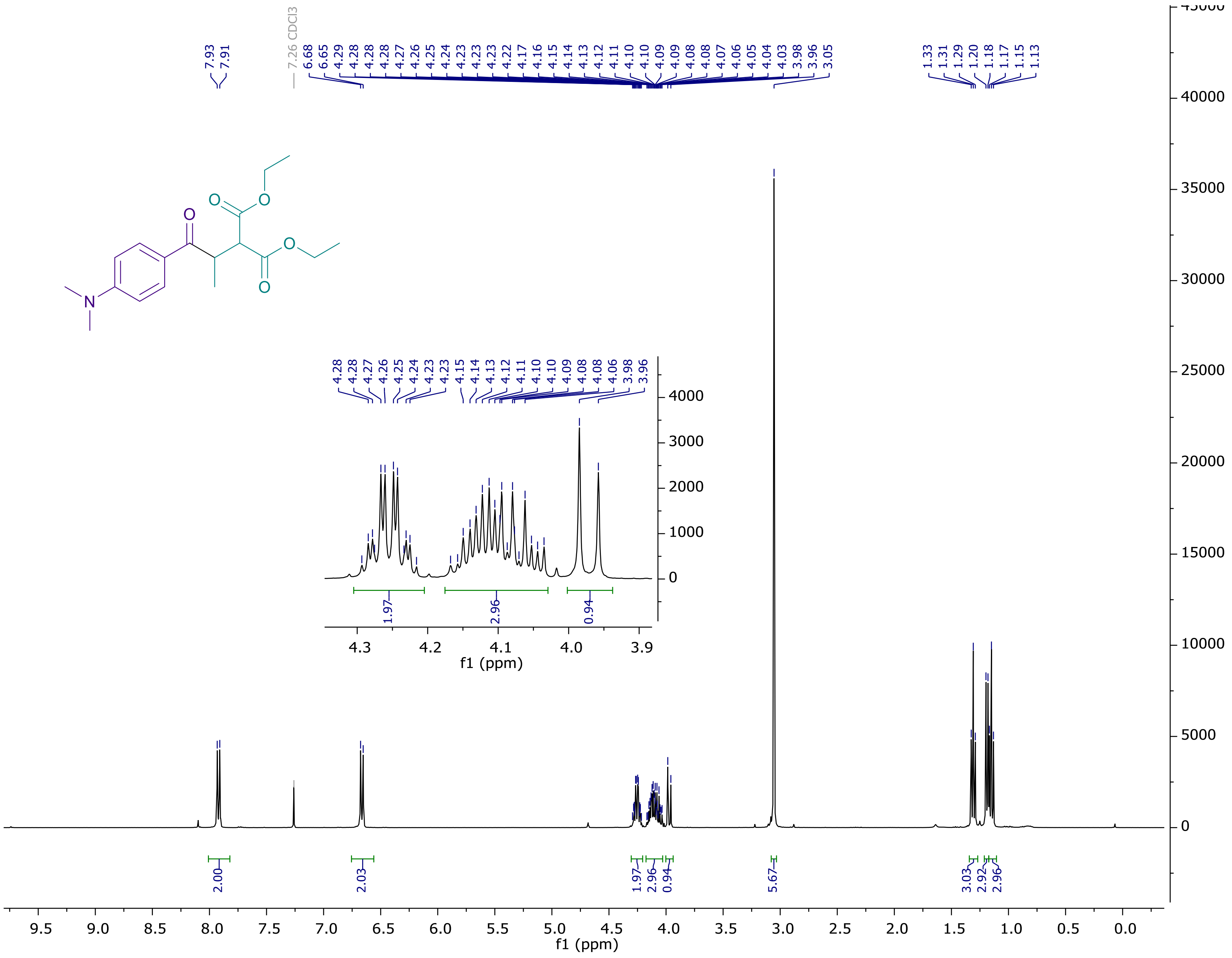

Compound 3e – $^{13}C\{^{1}H\}$ NMR (101 MHz, Chloroform-*d*):

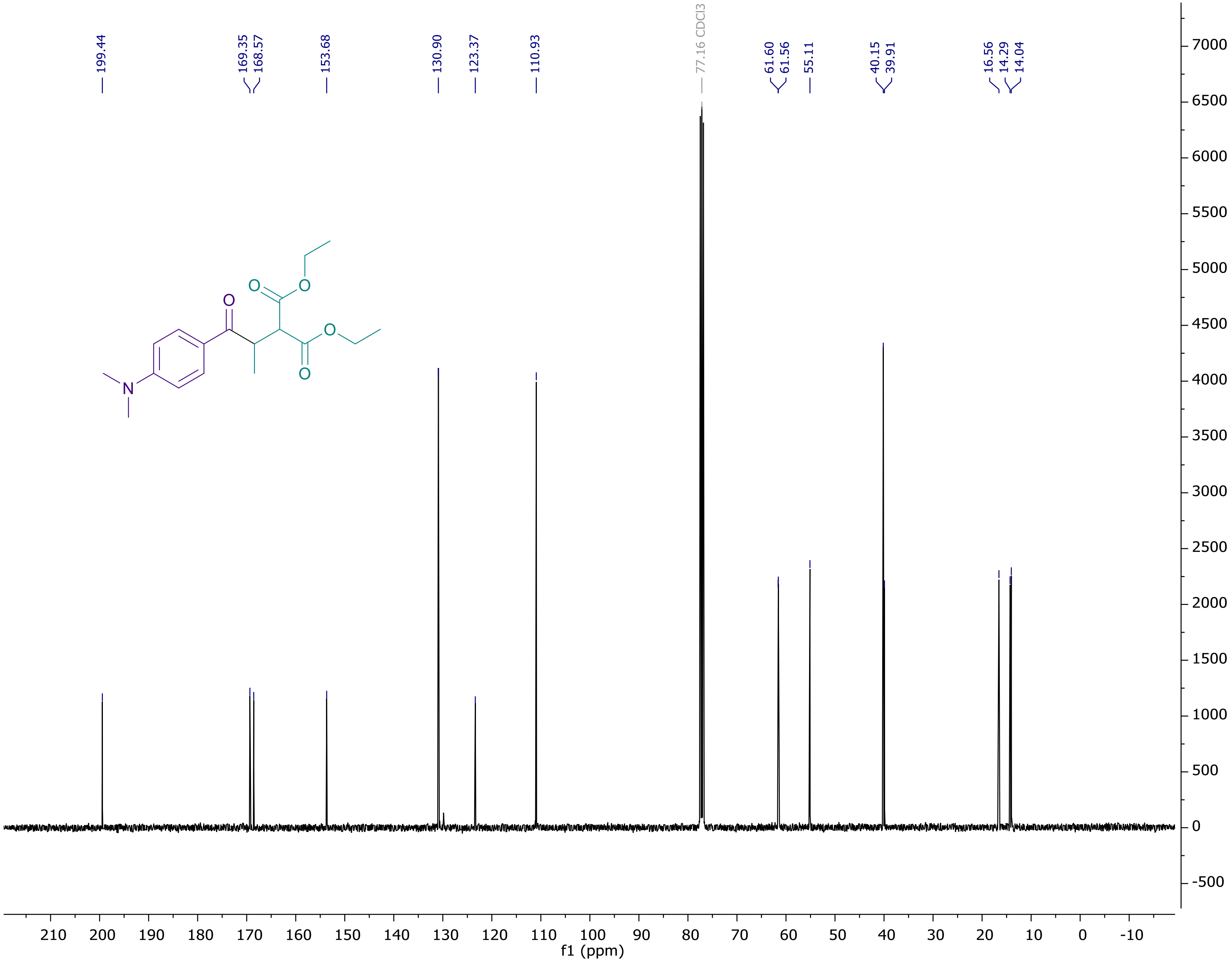

Compound 3f – ^{1}H NMR (400 MHz, Chloroform-*d*):

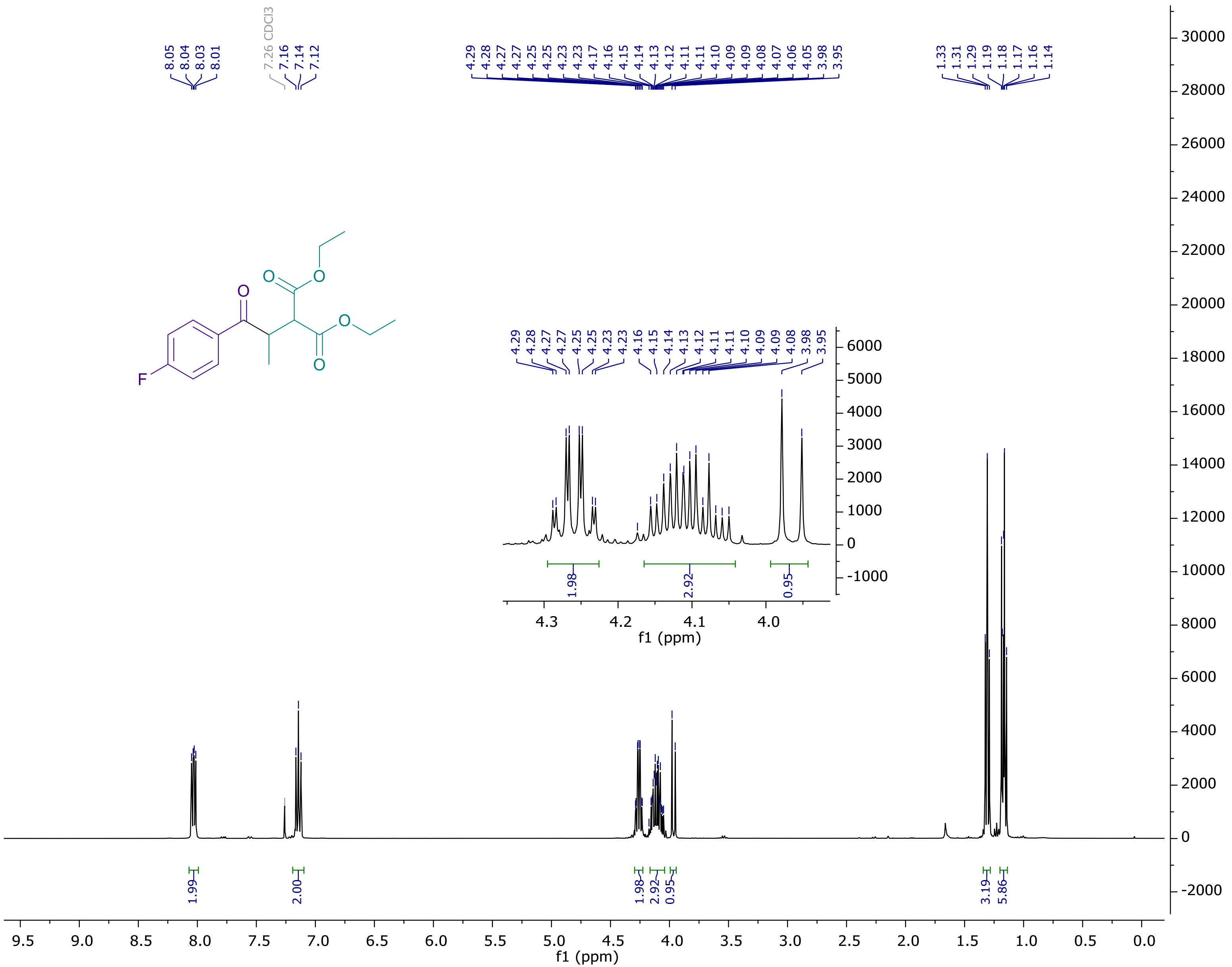

Compound 3f – $^{13}C\{^{1}H\}$ NMR (101 MHz, Chloroform-*d*):

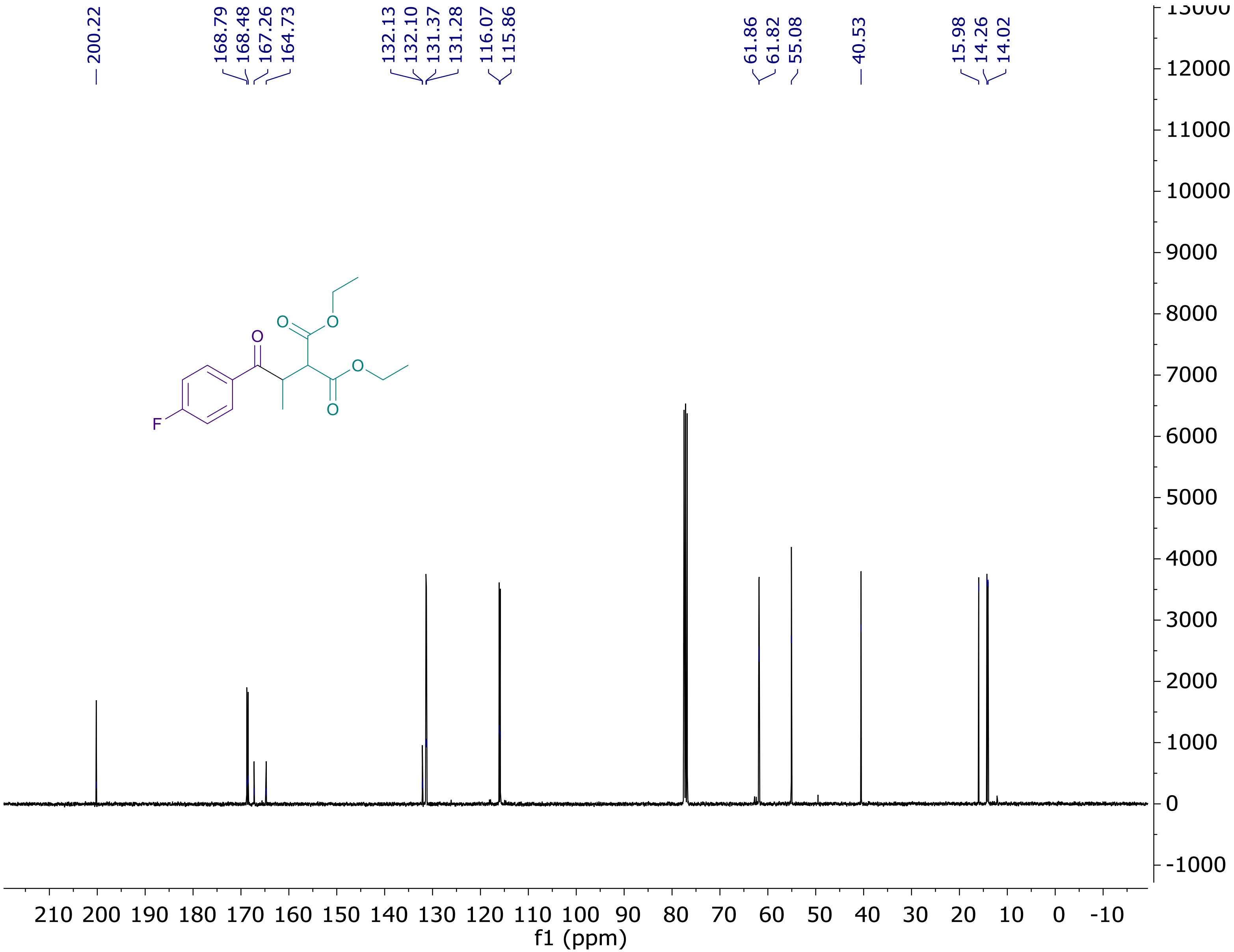

Compound 3f – ^{19}F NMR (376 MHz, Chloroform-*d*):

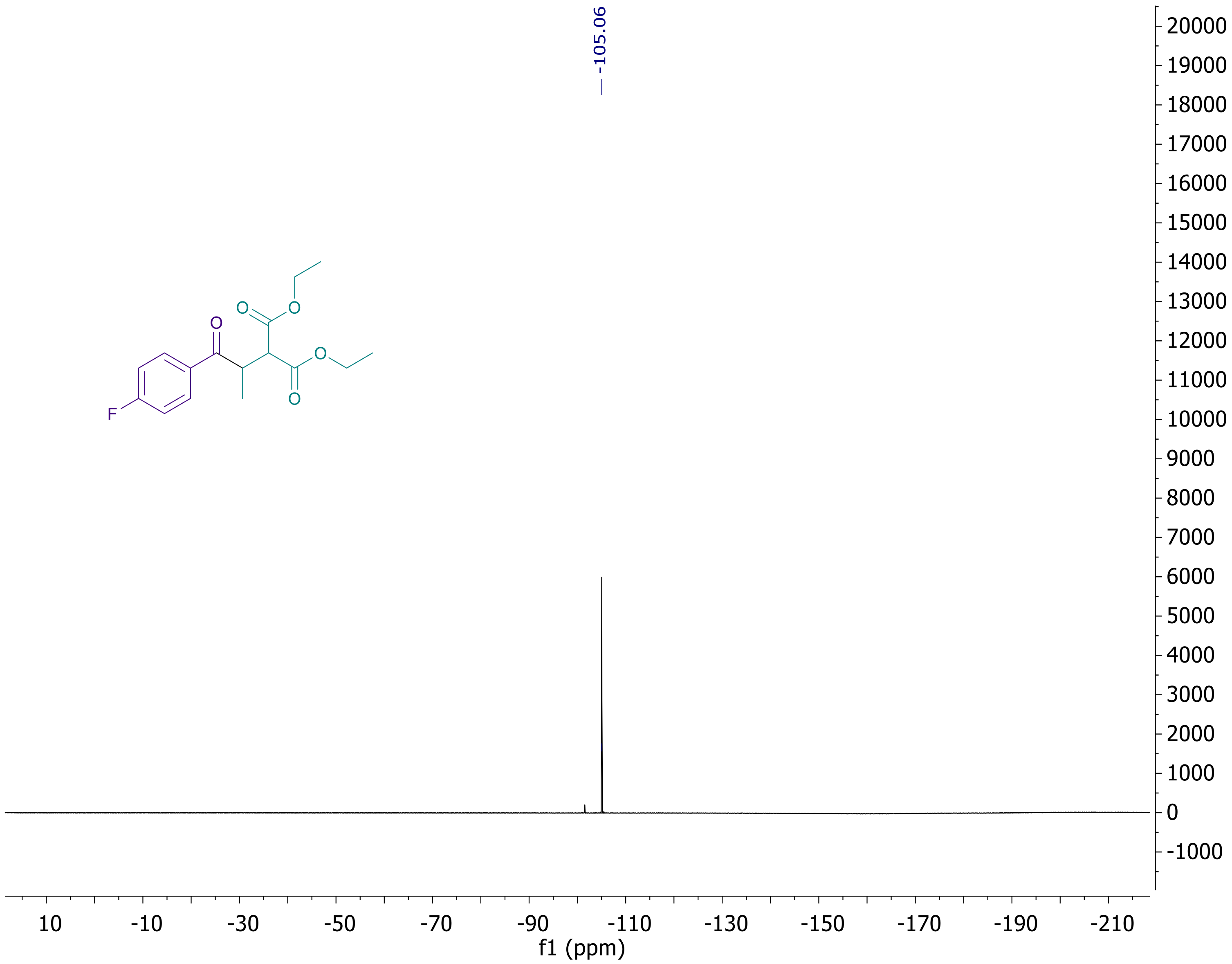

Compound 3g – ^{1}H NMR (400 MHz, Chloroform-*d*):

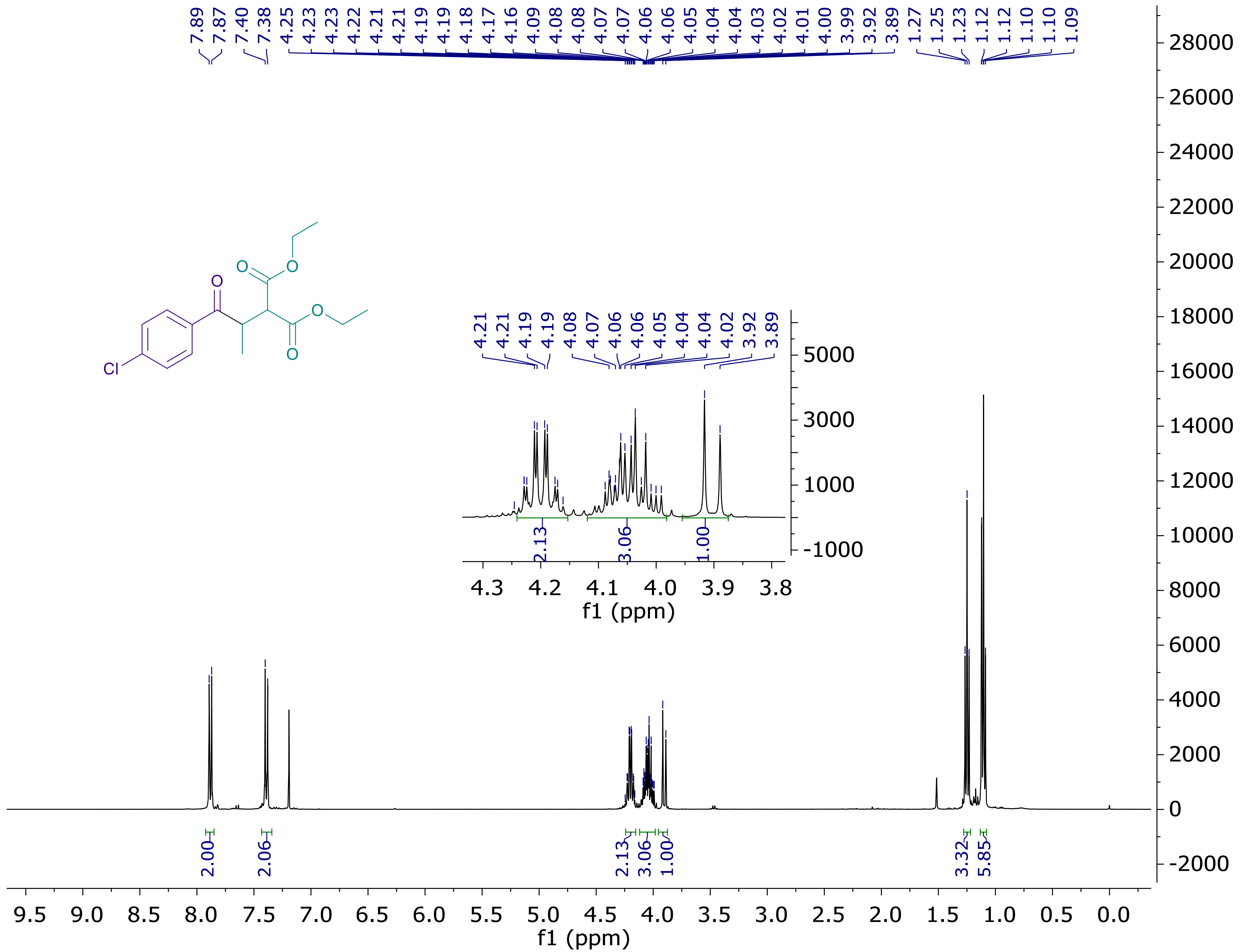

Compound 3g – $^{13}C\{^{1}H\}$ NMR (101 MHz, Chloroform-*d*):

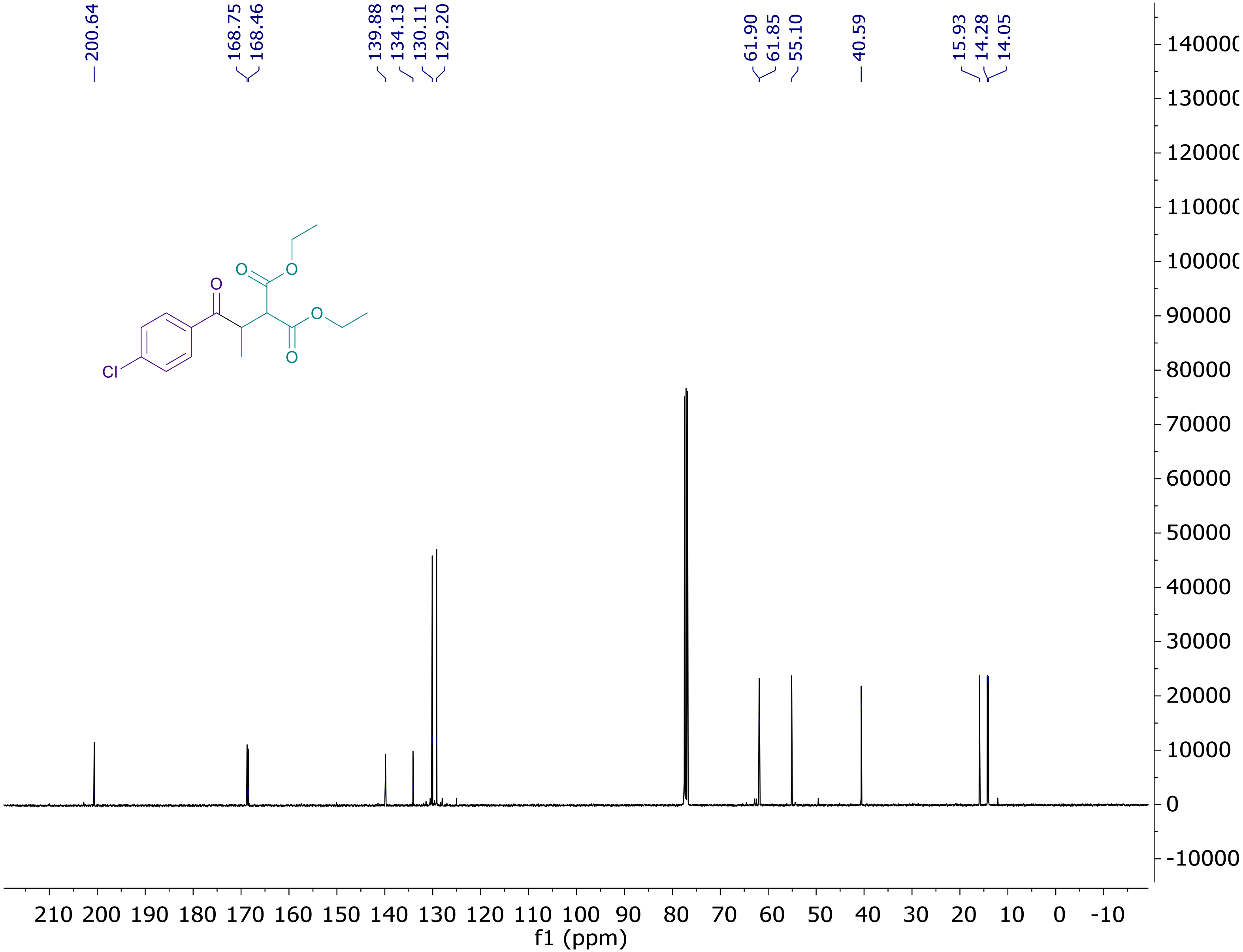

Compound 3h– ^{1}H NMR (400 MHz, Chloroform-*d*):

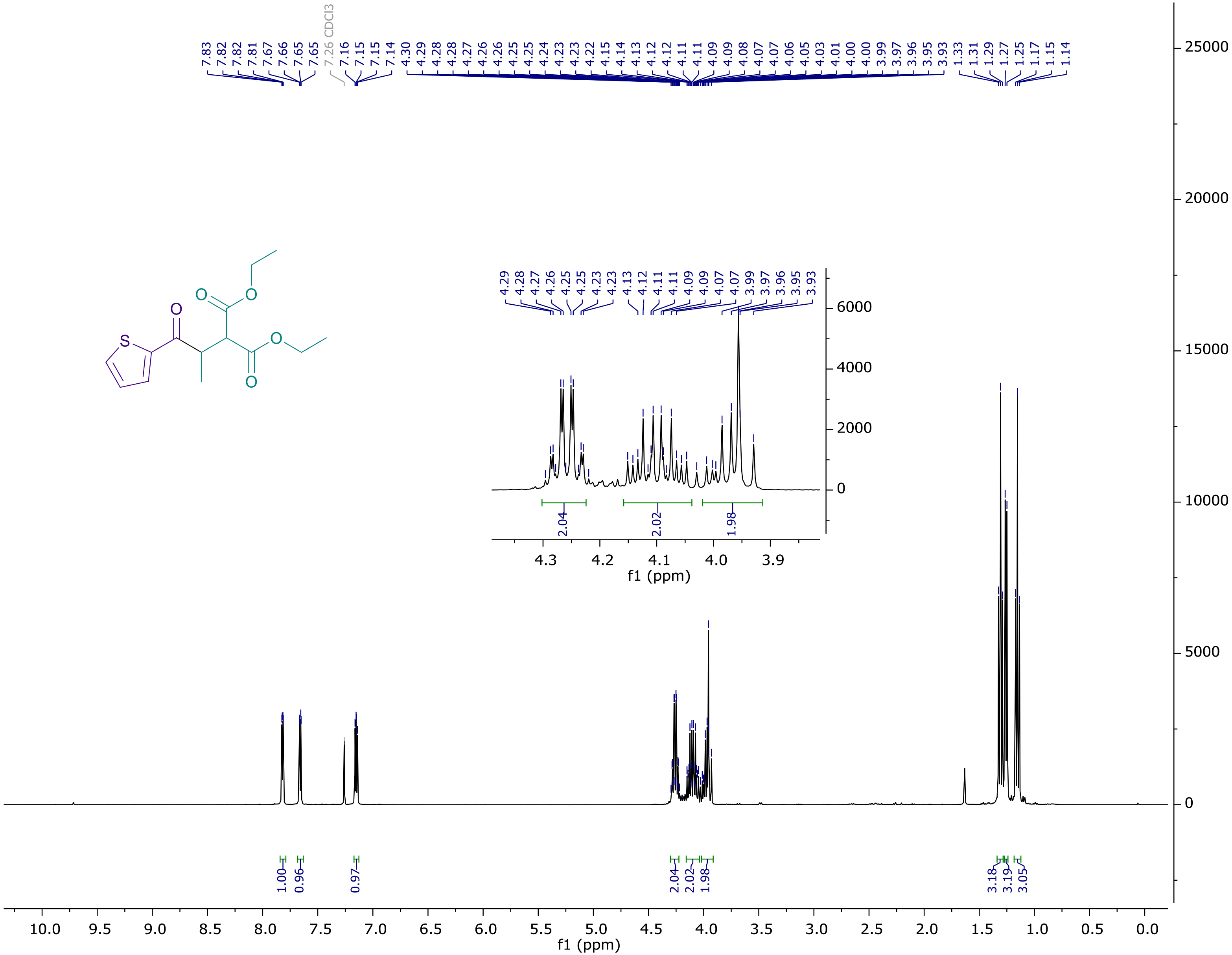

Compound 3h – $^{13}C\{^{1}H\}$ NMR (101 MHz, Chloroform-*d*):

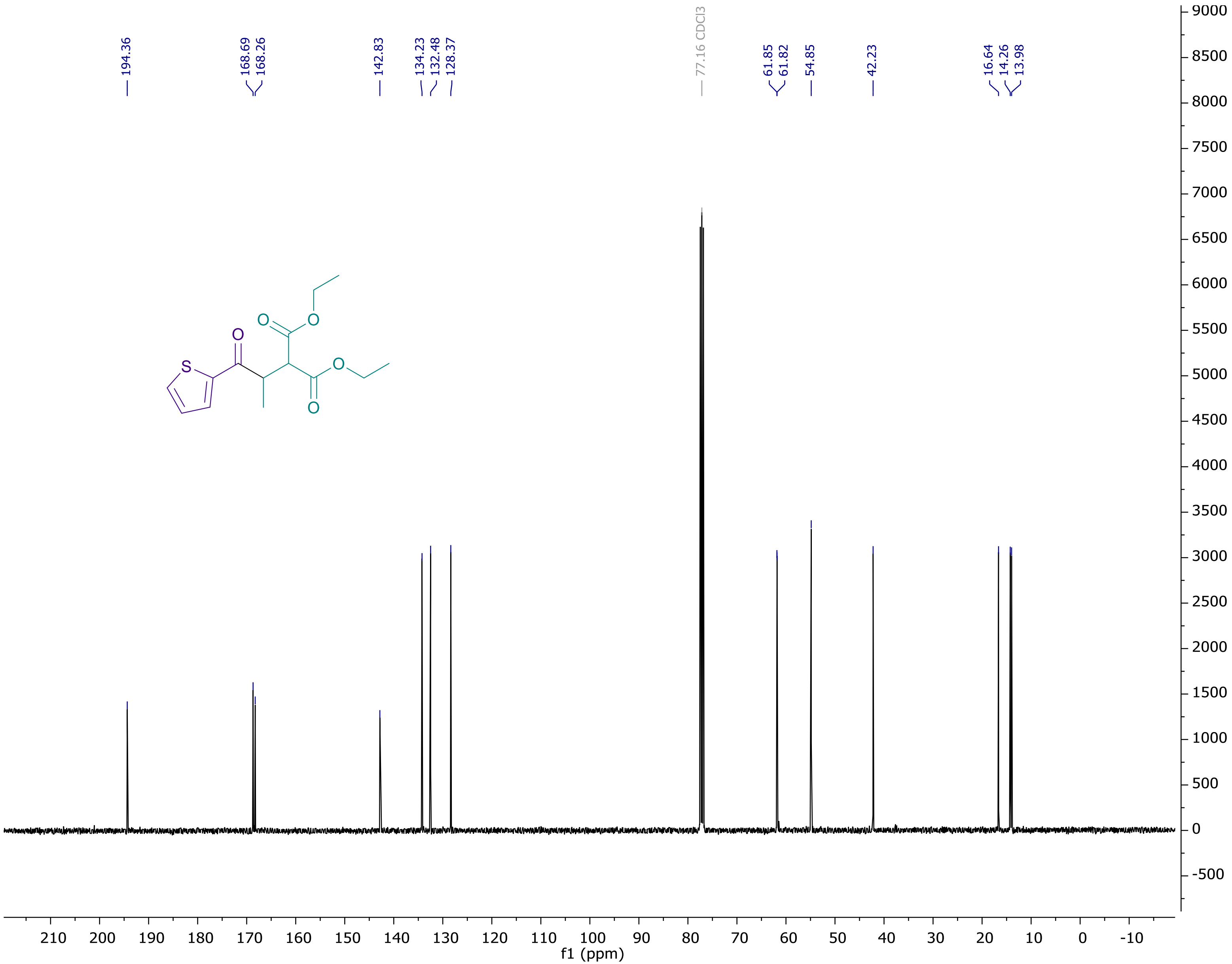

Compound 3i – ^{1}H NMR (400 MHz, Chloroform-*d*):

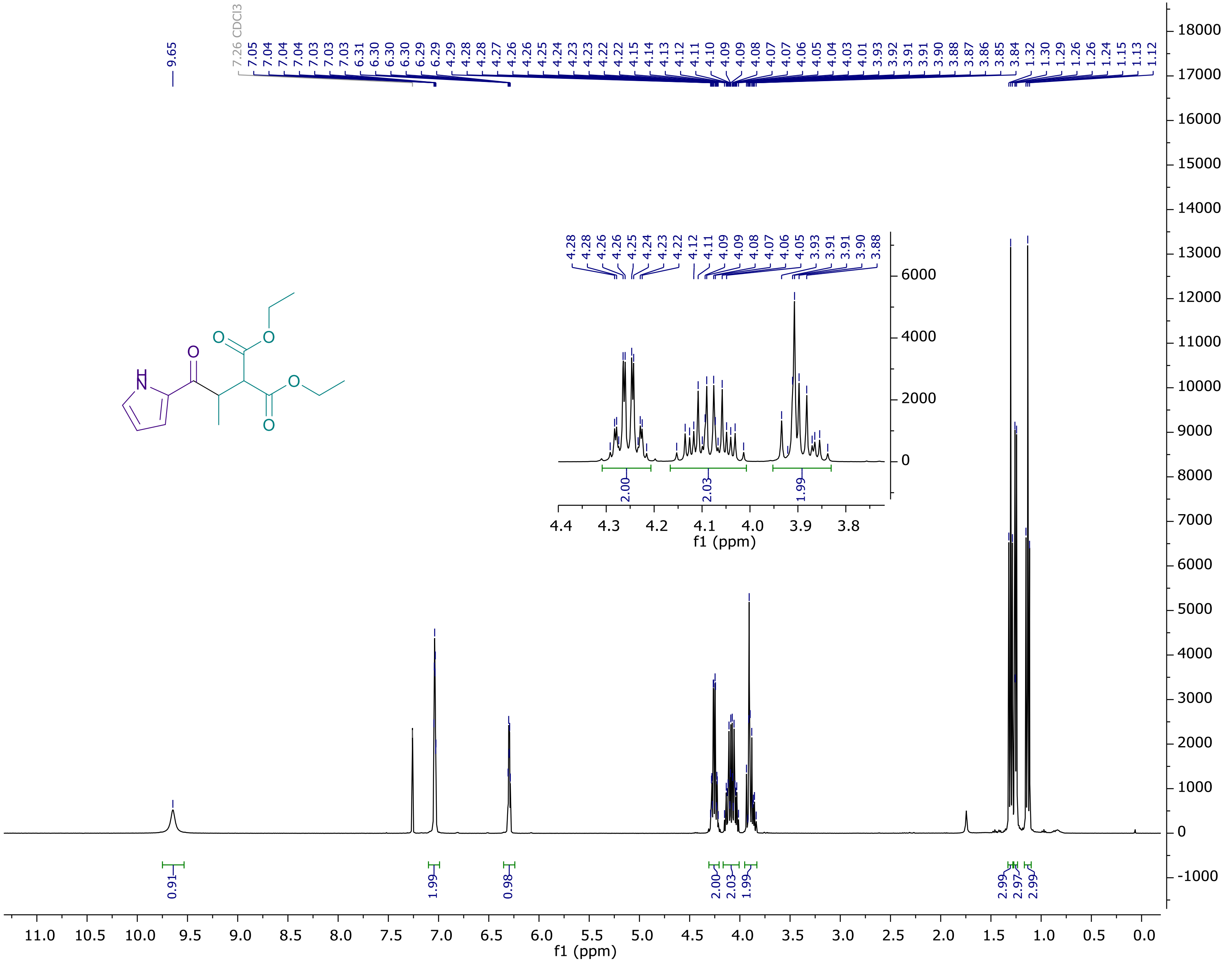

Compound 3i – $^{13}C\{^{1}H\}$ NMR (101 MHz, Chloroform-*d*):

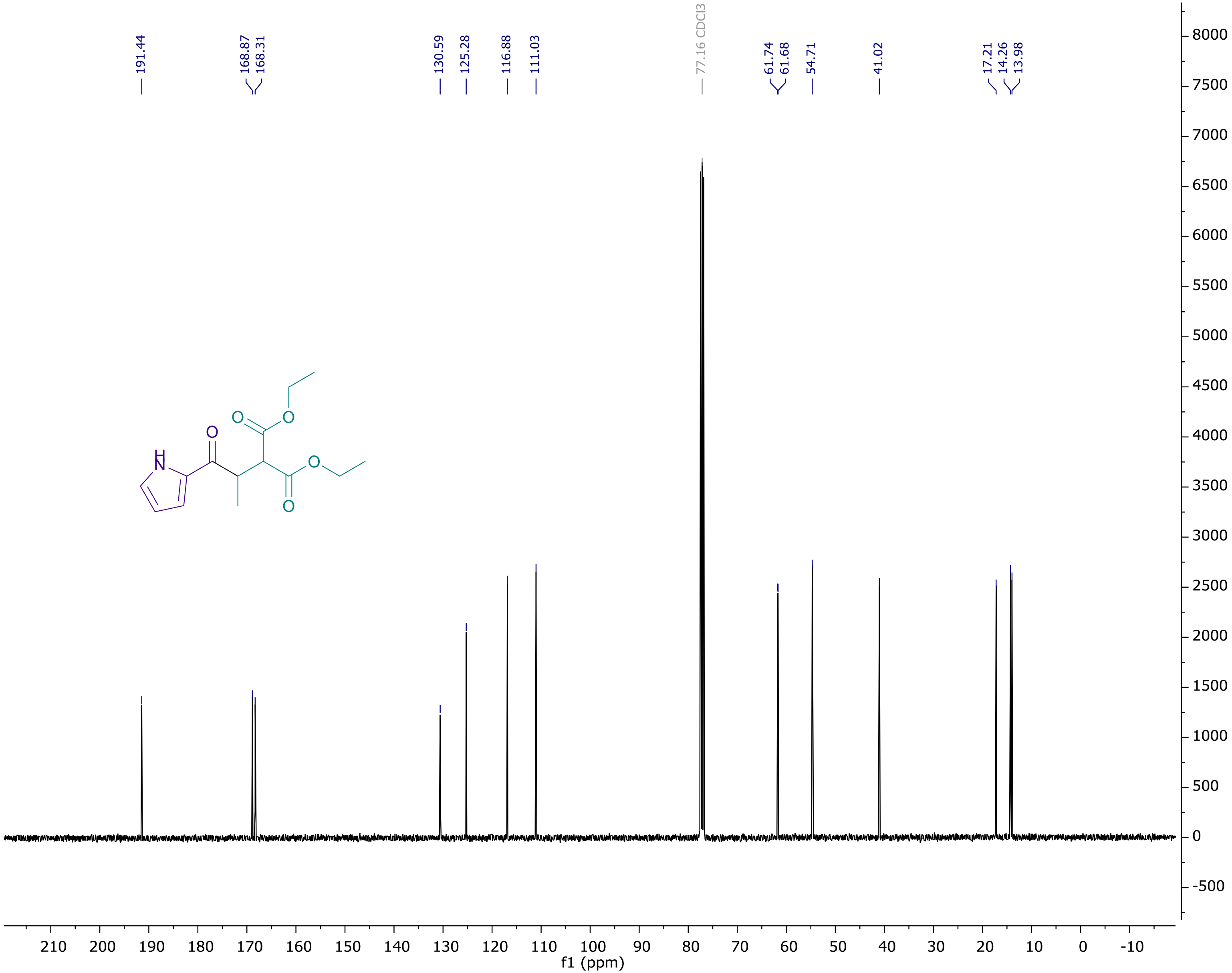

Compound 3j – ^{1}H NMR (400 MHz, Chloroform-*d*):

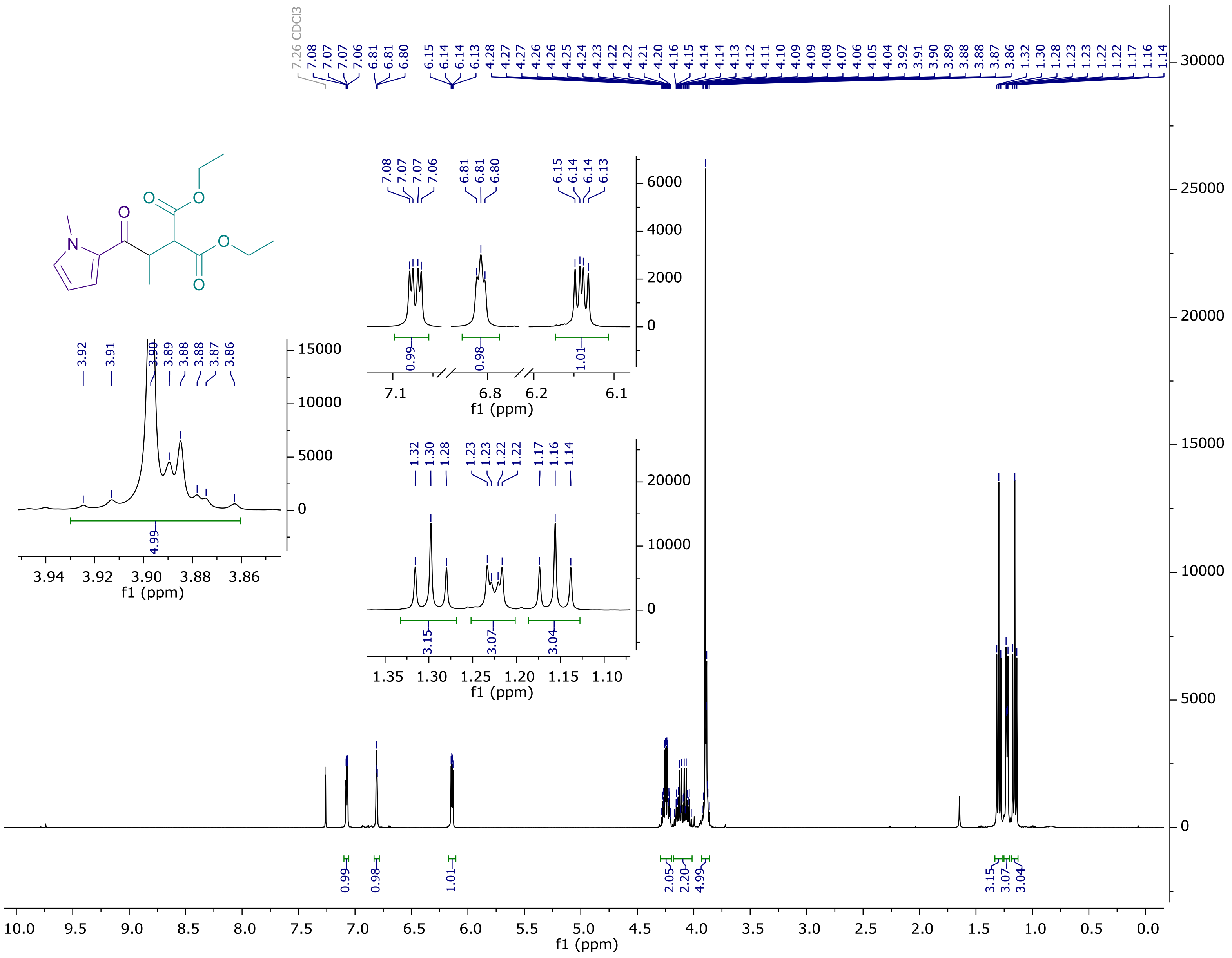

Compound 3j – $^{13}C\{^{1}H\}$ NMR (101 MHz, Chloroform-*d*):

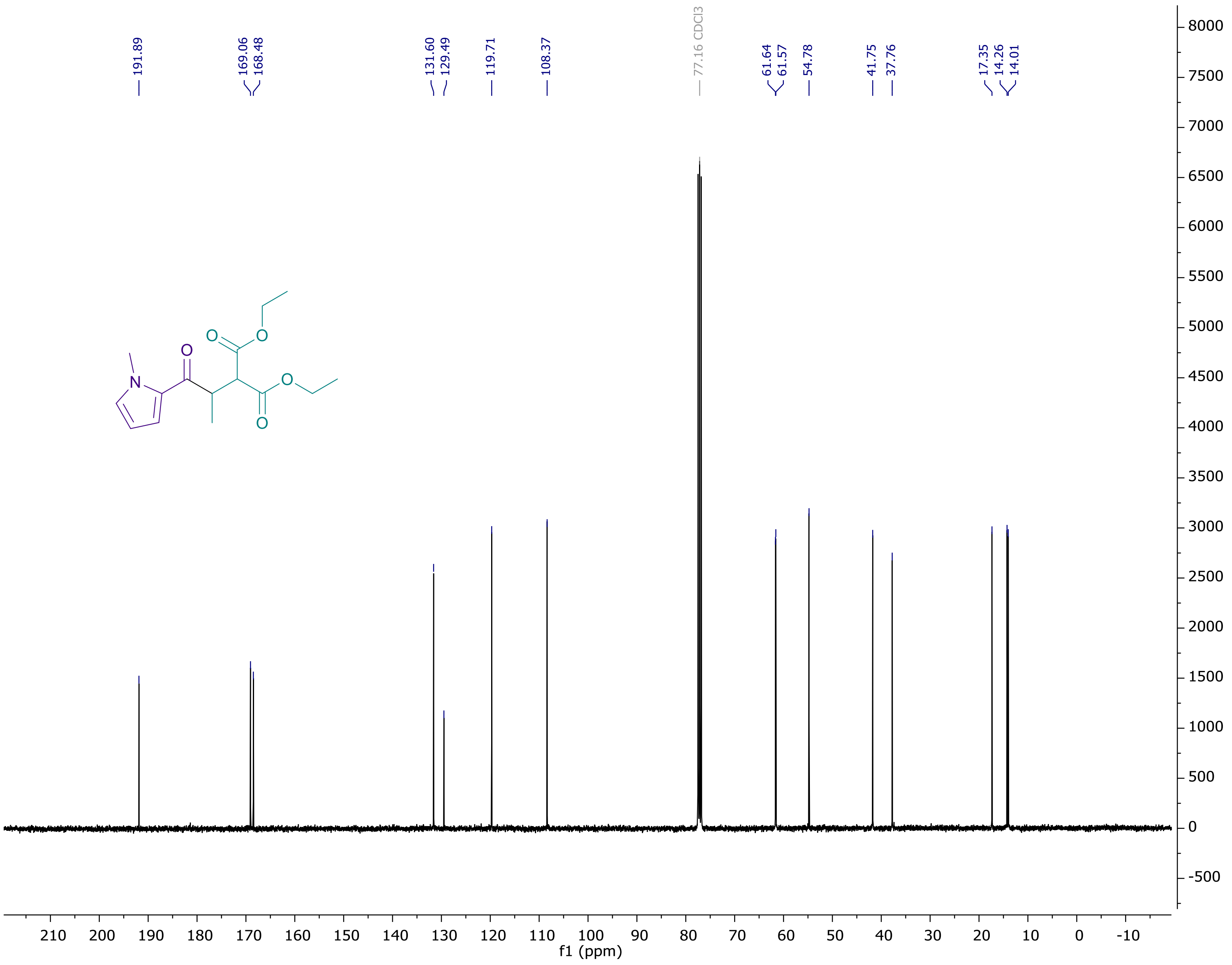

Compound 3k – ^{1}H NMR (400 MHz, Chloroform-*d*):

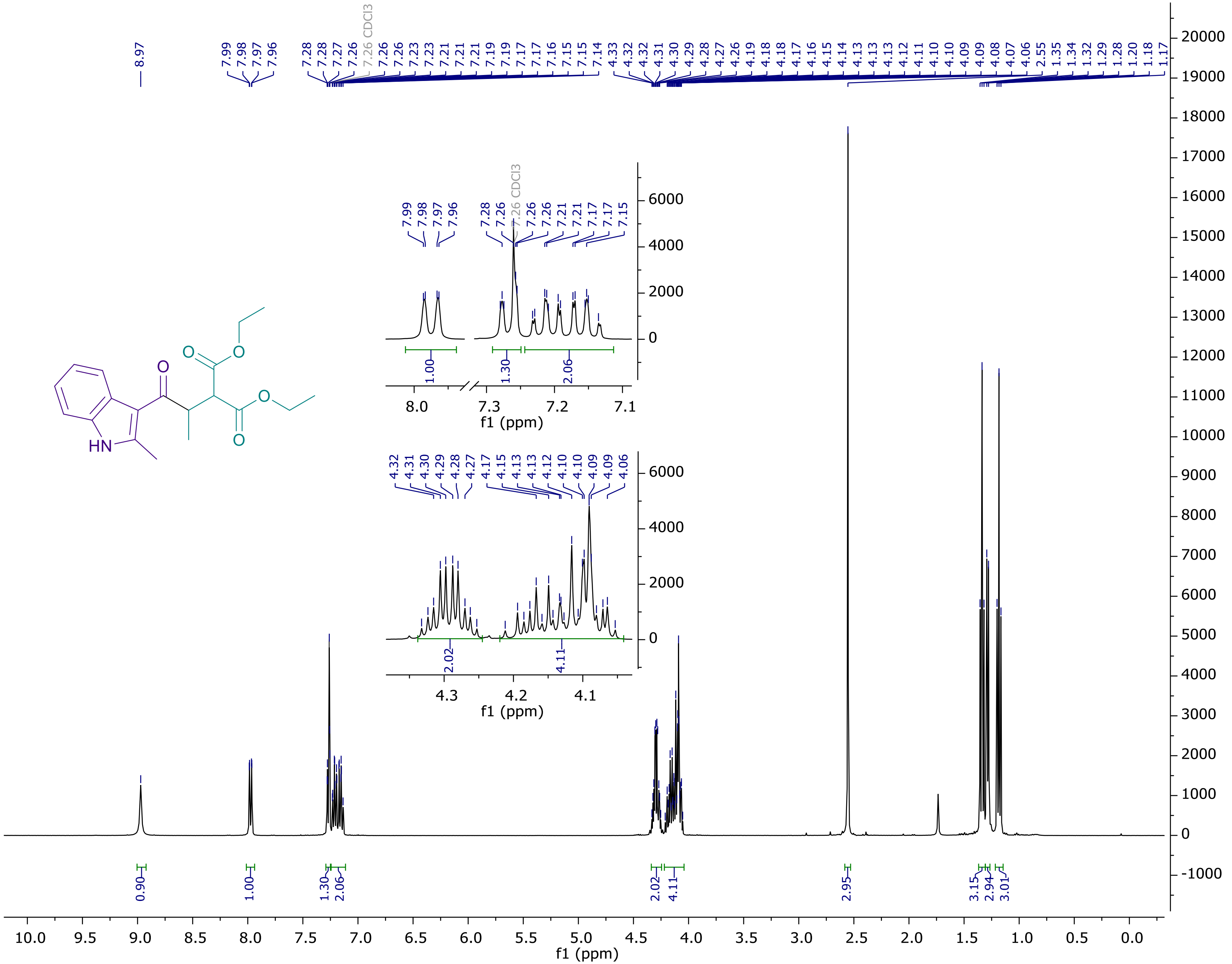

Compound 3k – $^{13}C\{^{1}H\}$ NMR (101 MHz, Chloroform-*d*):

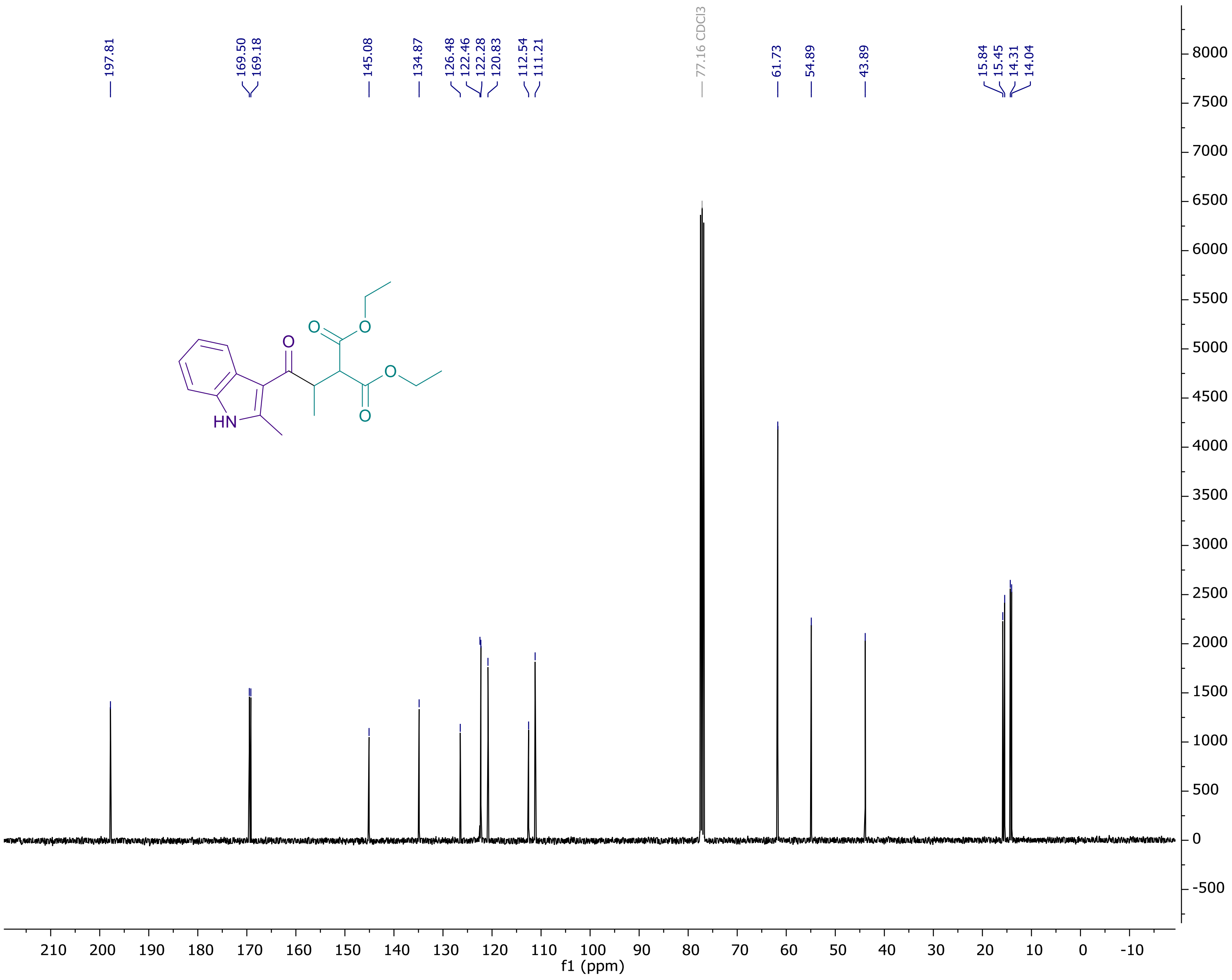

Compound 3m – ^{1}H NMR (400 MHz, Chloroform-*d*):

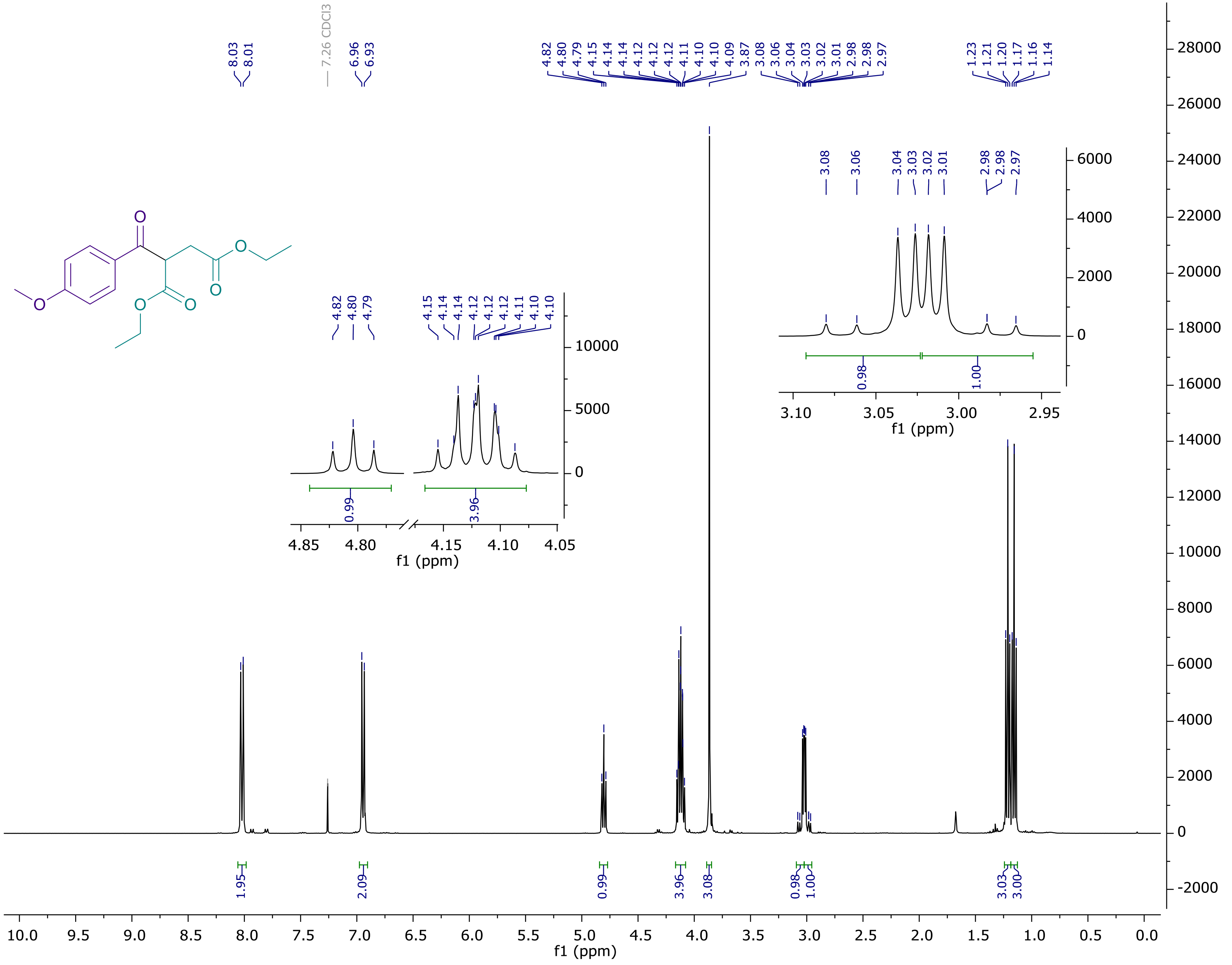

Compound 3m – $^{13}C\{^{1}H\}$ NMR (101 MHz, Chloroform-*d*):

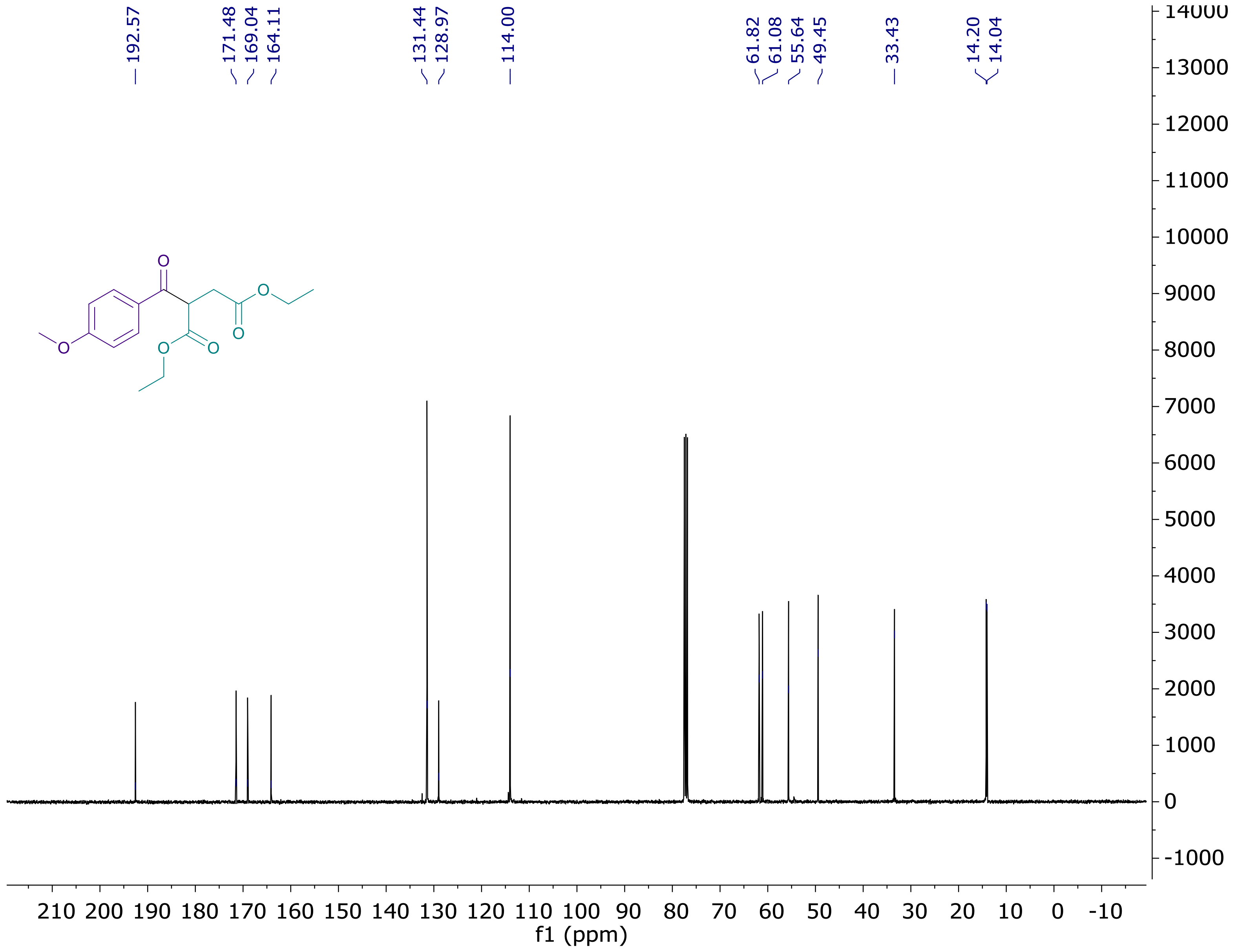

Compound 3n – ¹H NMR (400 MHz, Chloroform-*d*):

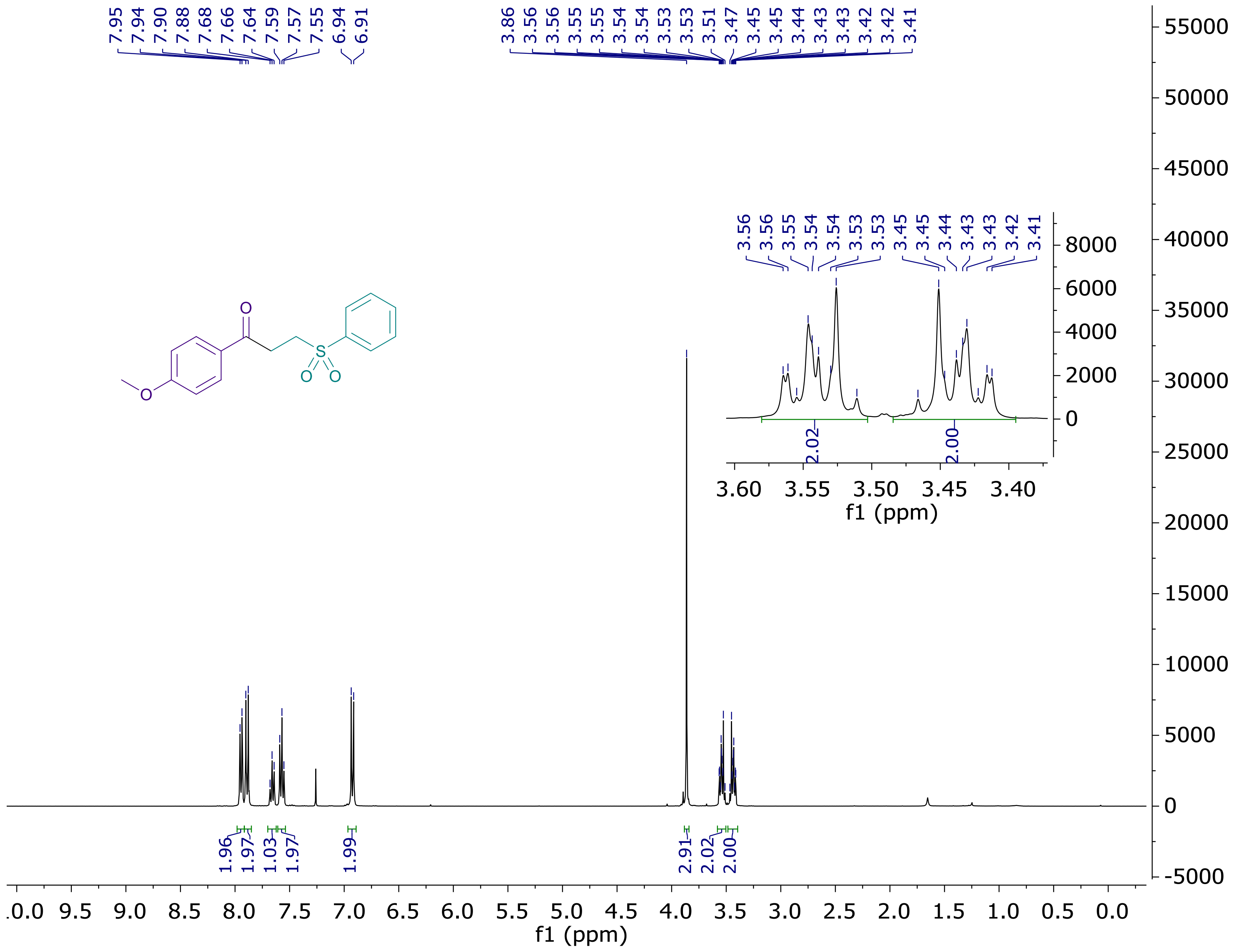

Compound 3n – $^{13}C\{^{1}H\}$ NMR (101 MHz, Chloroform-*d*):

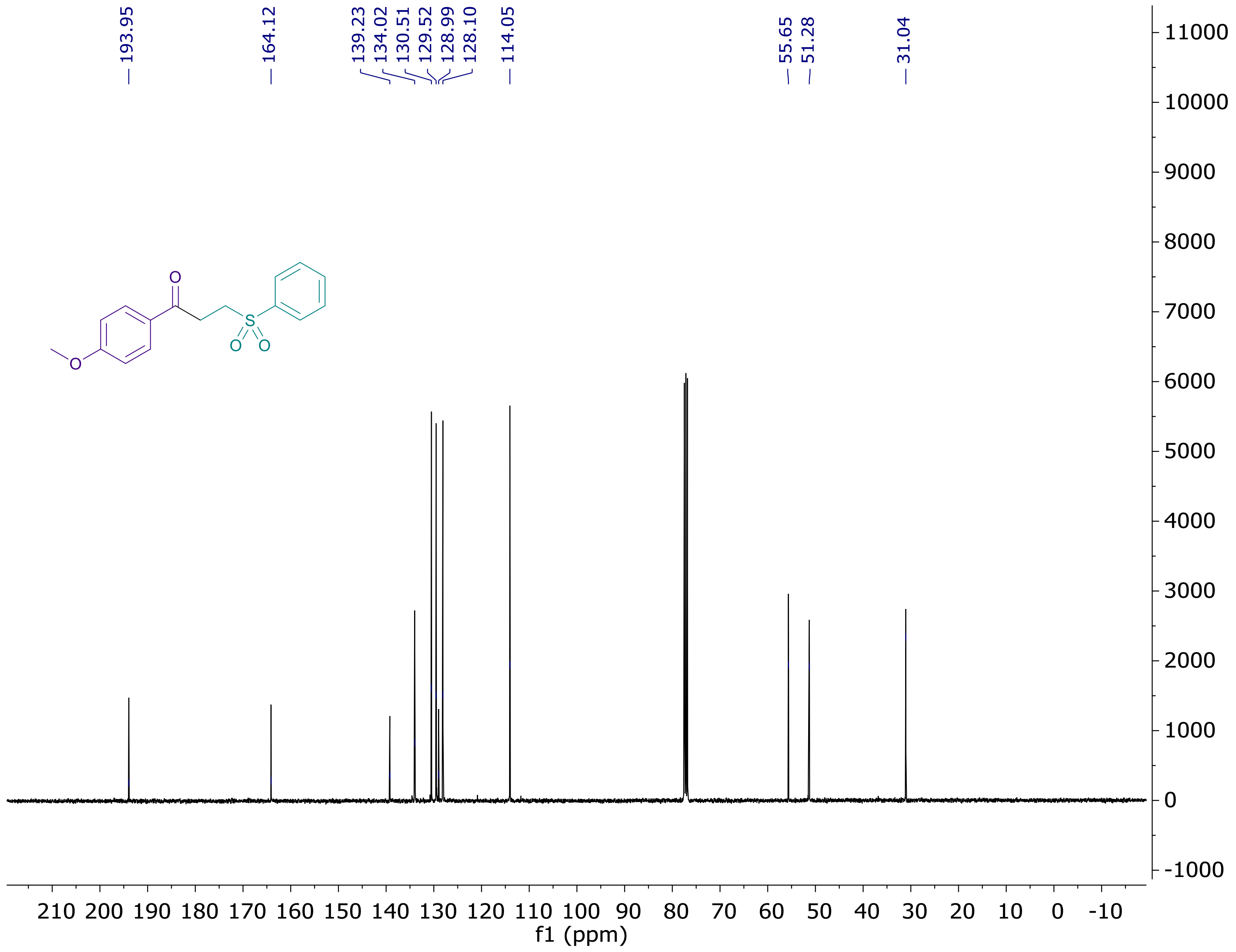

Compound 3o – ^{1}H NMR (400 MHz, Chloroform-*d*):

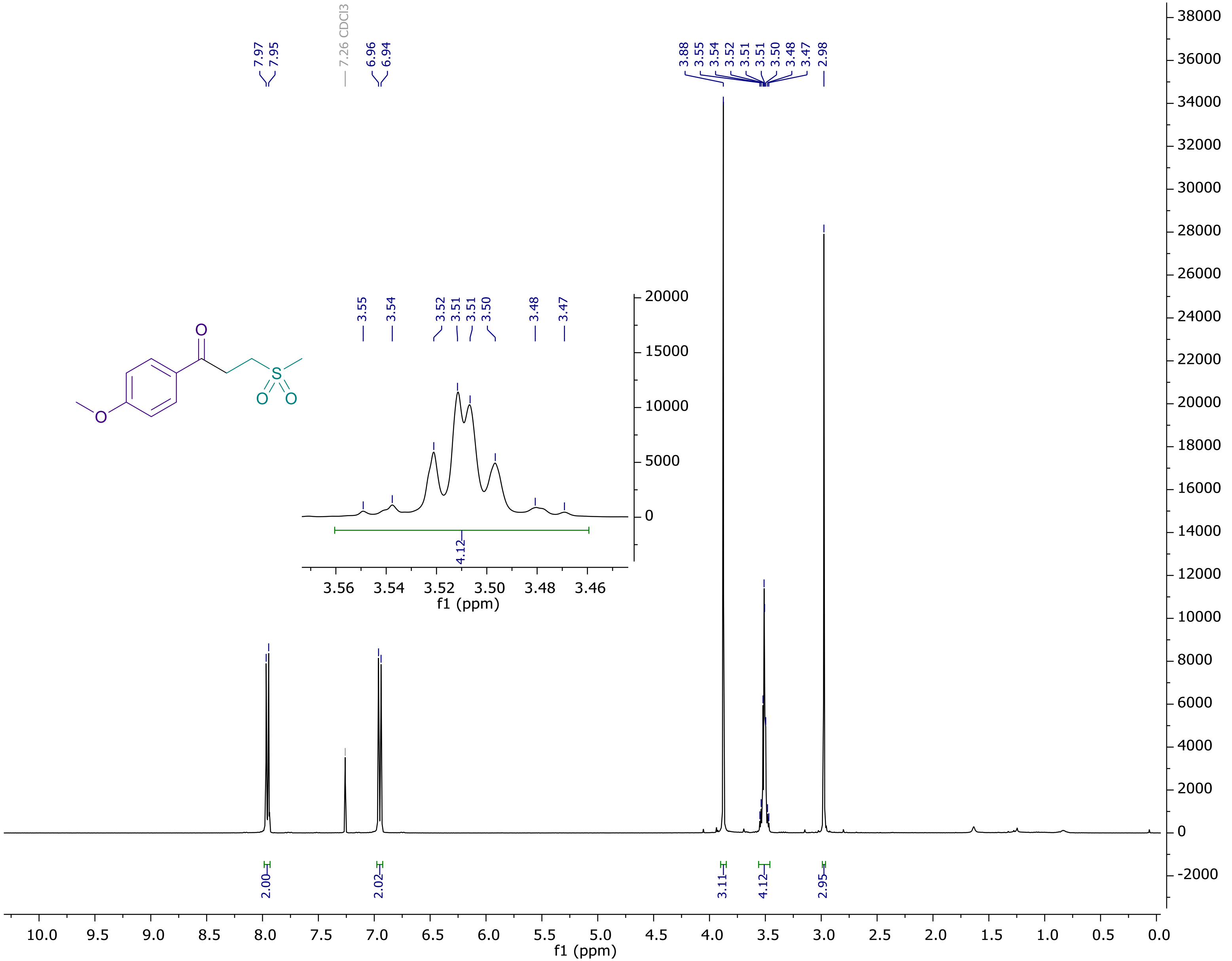

Compound 3o – $^{13}C\{^1H\}$ NMR (101 MHz, Chloroform-*d*):

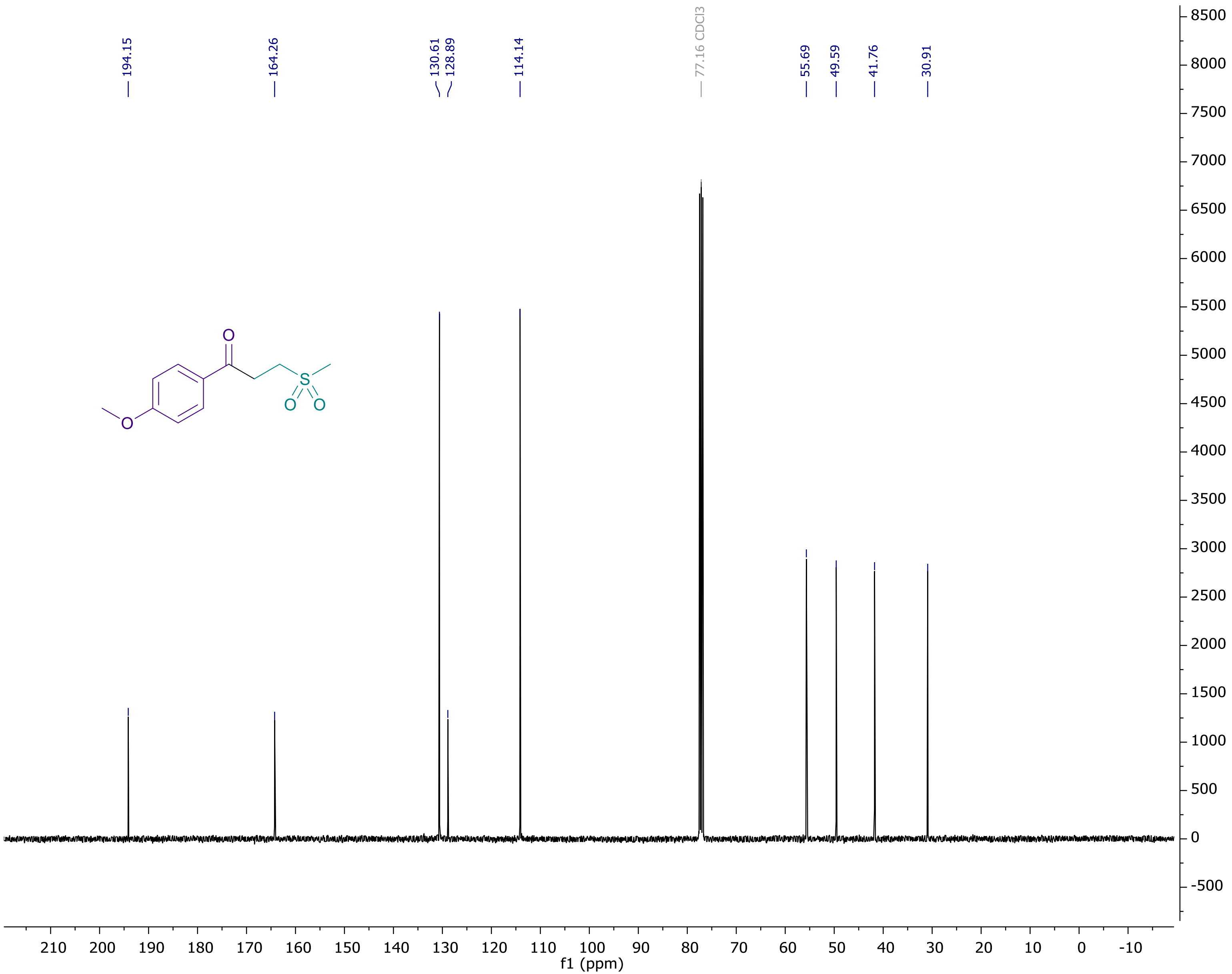

Compound 3p – ^{1}H NMR (400 MHz, Chloroform-*d*):

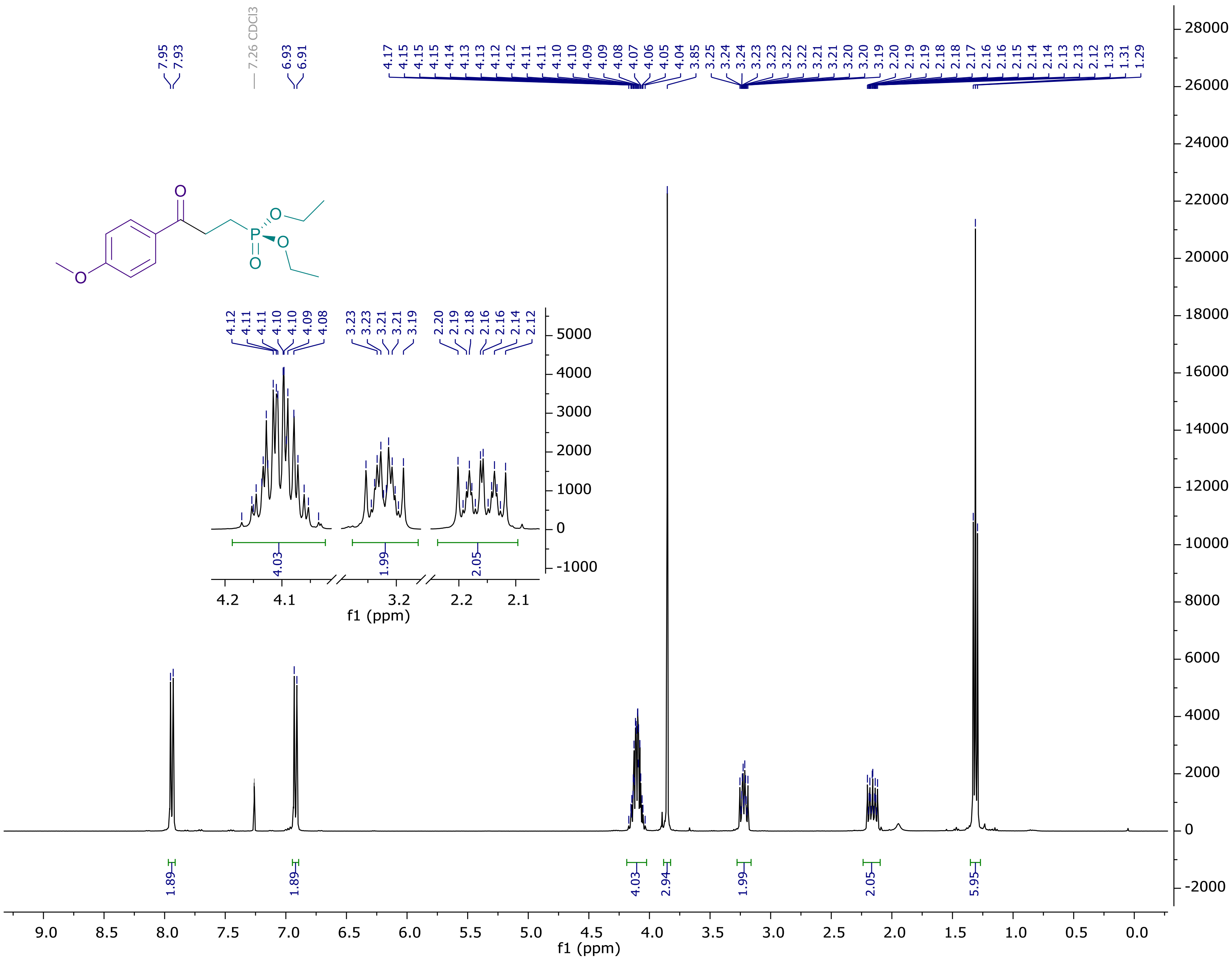

Compound 3p – $^{13}C\{^{1}H\}$ NMR (101 MHz, Chloroform-*d*):

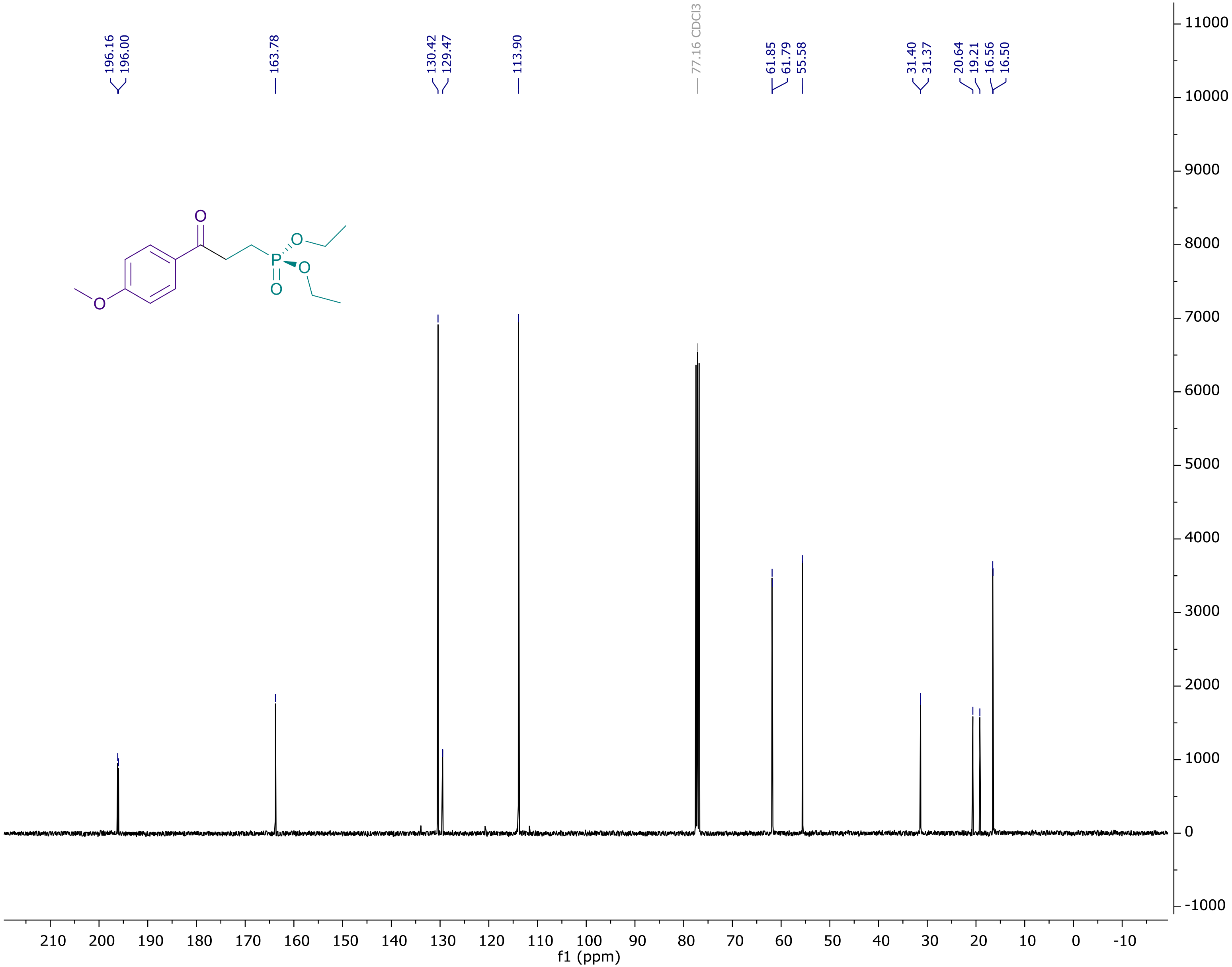

Compound 3p – ^{31}P NMR (162 MHz, Chloroform-*d*):

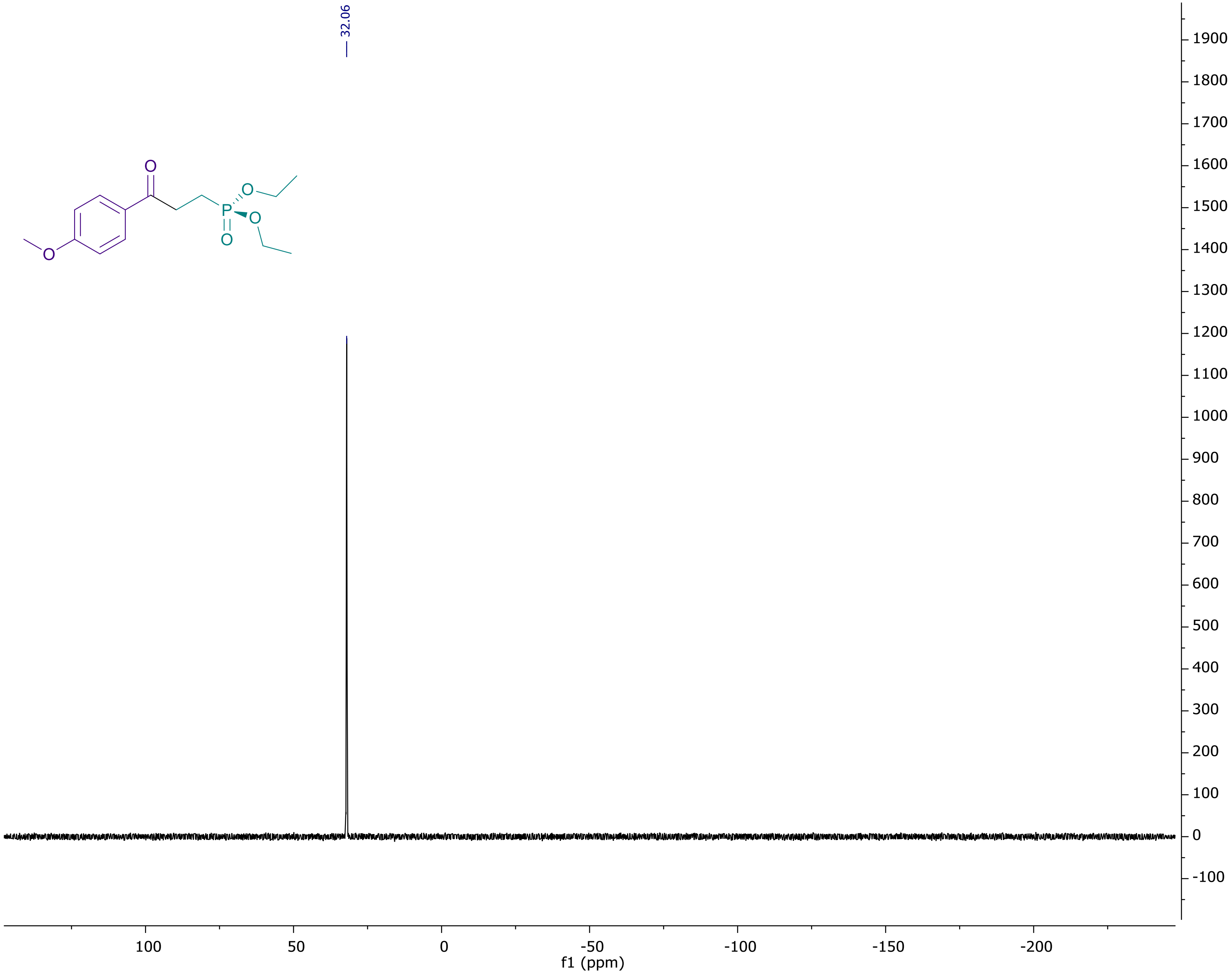

Compound 3q (major diasteromer) – ^{1}H NMR (400 MHz, Chloroform-*d*):

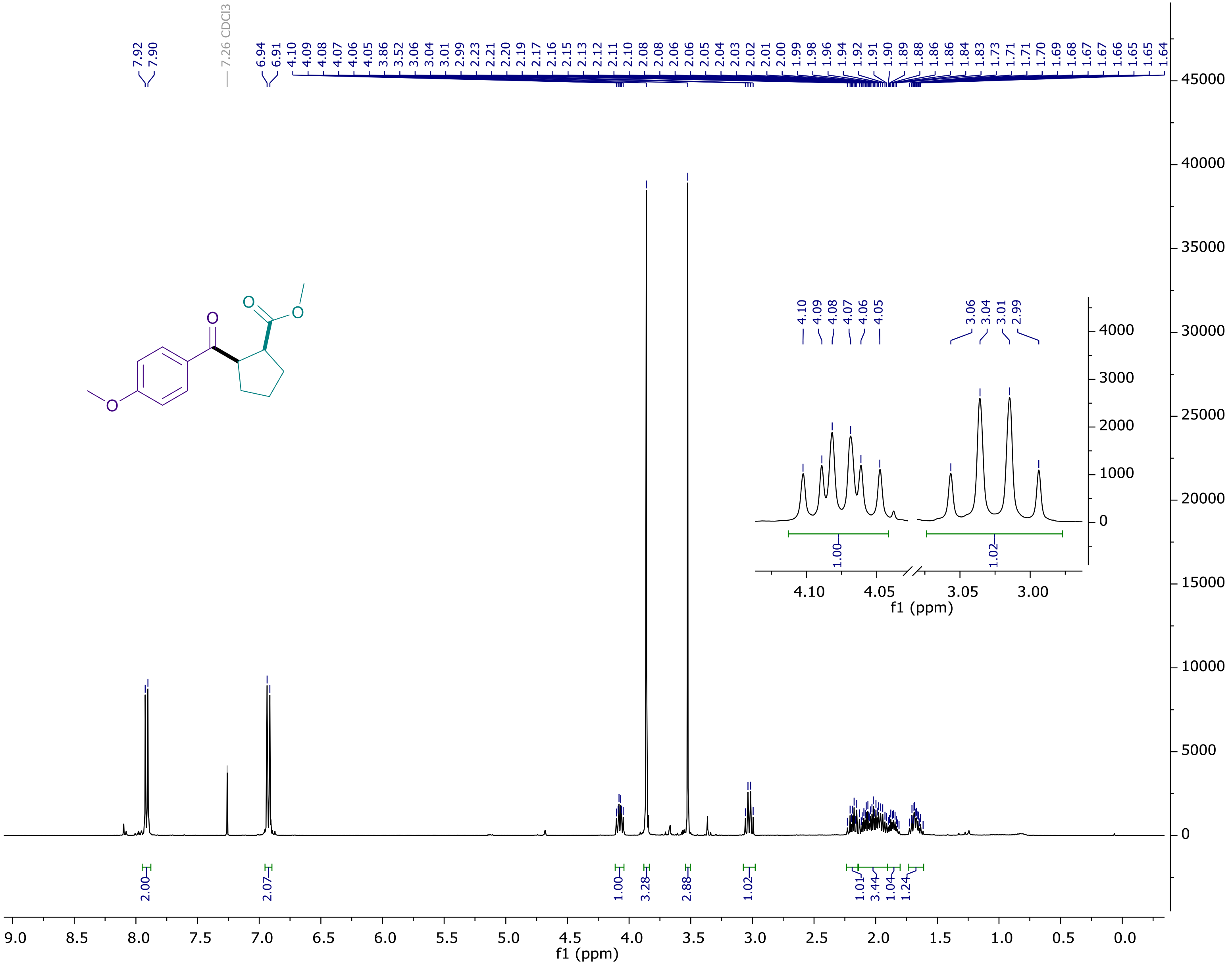

Compound 3q (major diastereomer) – $^{13}C\{^1H\}$ NMR (101 MHz, Chloroform-*d*):

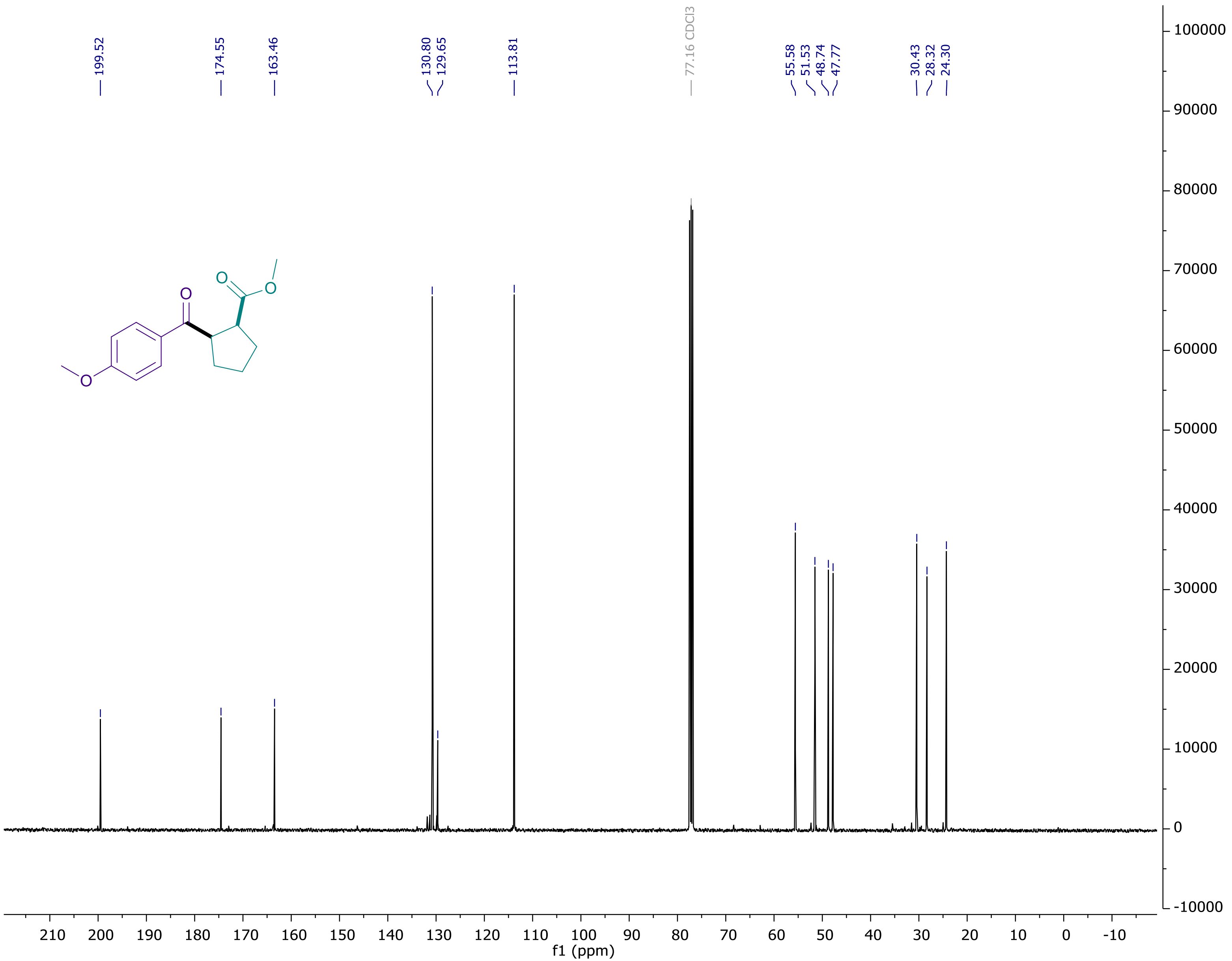

Compound 3q (minor diastereomer) – ^{1}H NMR (400 MHz, Chloroform-*d*):

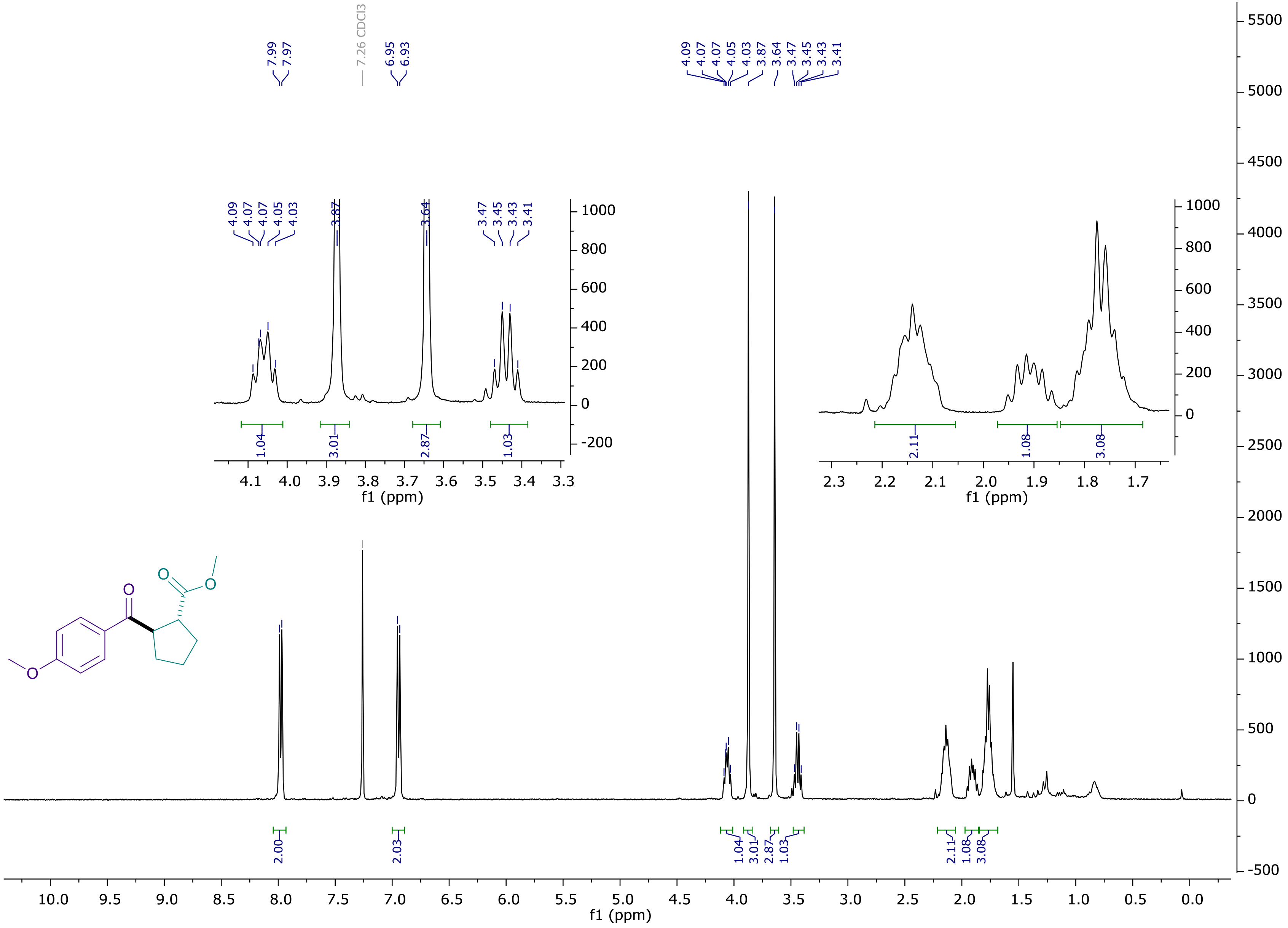

Compound 3q (minor diastereomer) – $^{13}C\{^1H\}$ NMR (101 MHz, Chloroform-*d*):

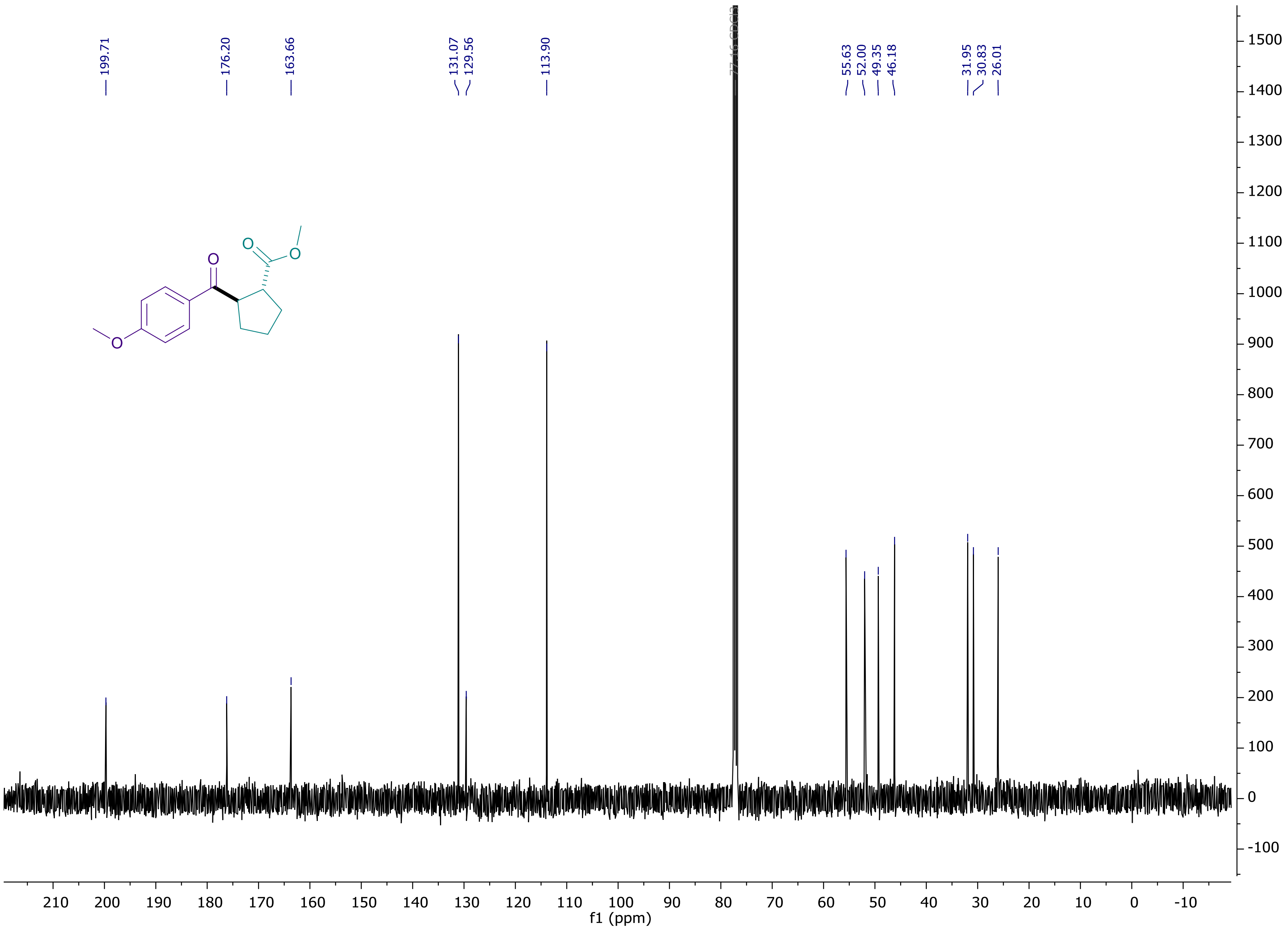

Compound 3r (major diastereomer) – ^{1}H NMR (400 MHz, Chloroform-*d*):

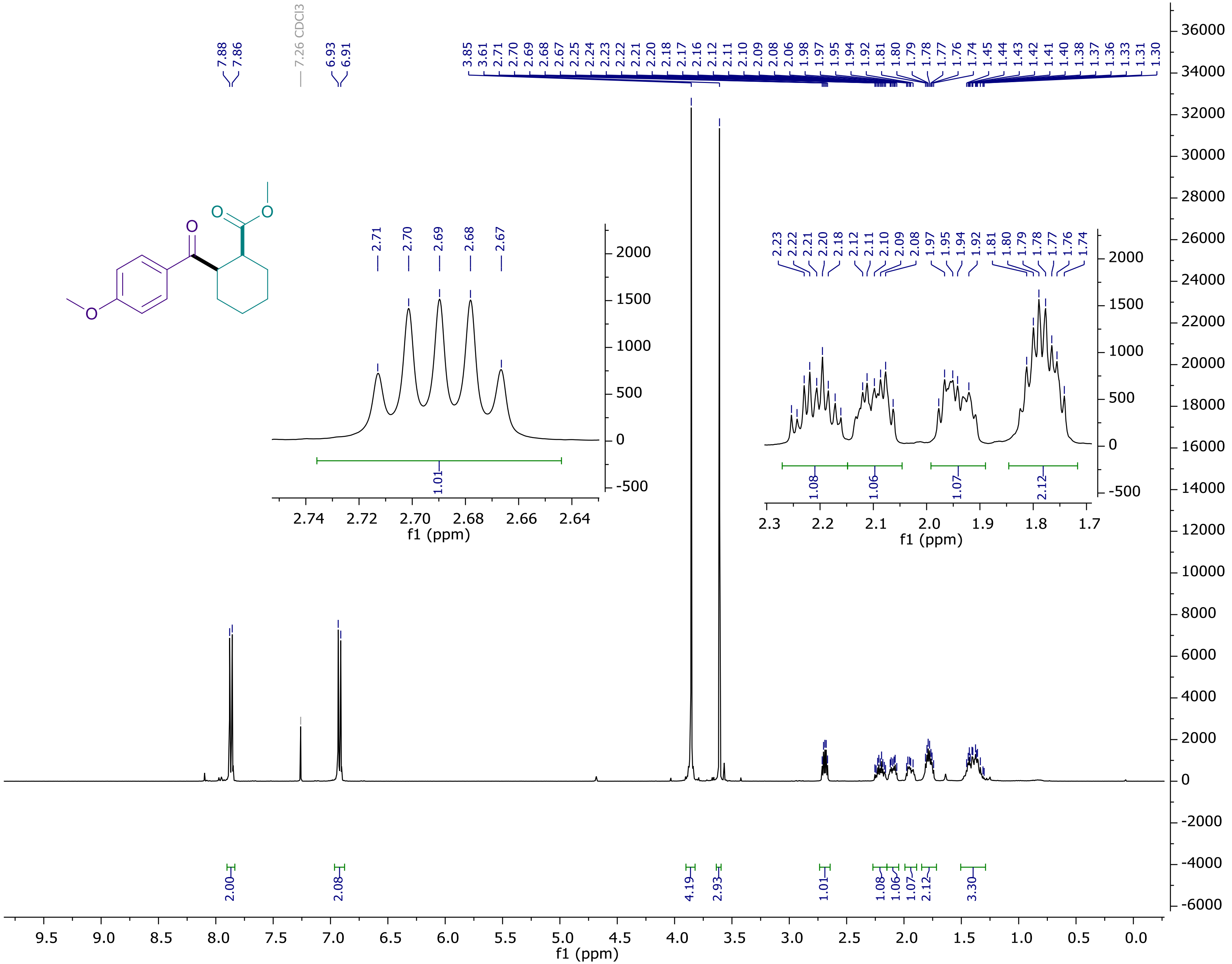

Compound 3r (major diastereomer) – $^{13}C\{^{1}H\}$ NMR (101 MHz, Chloroform-*d*):

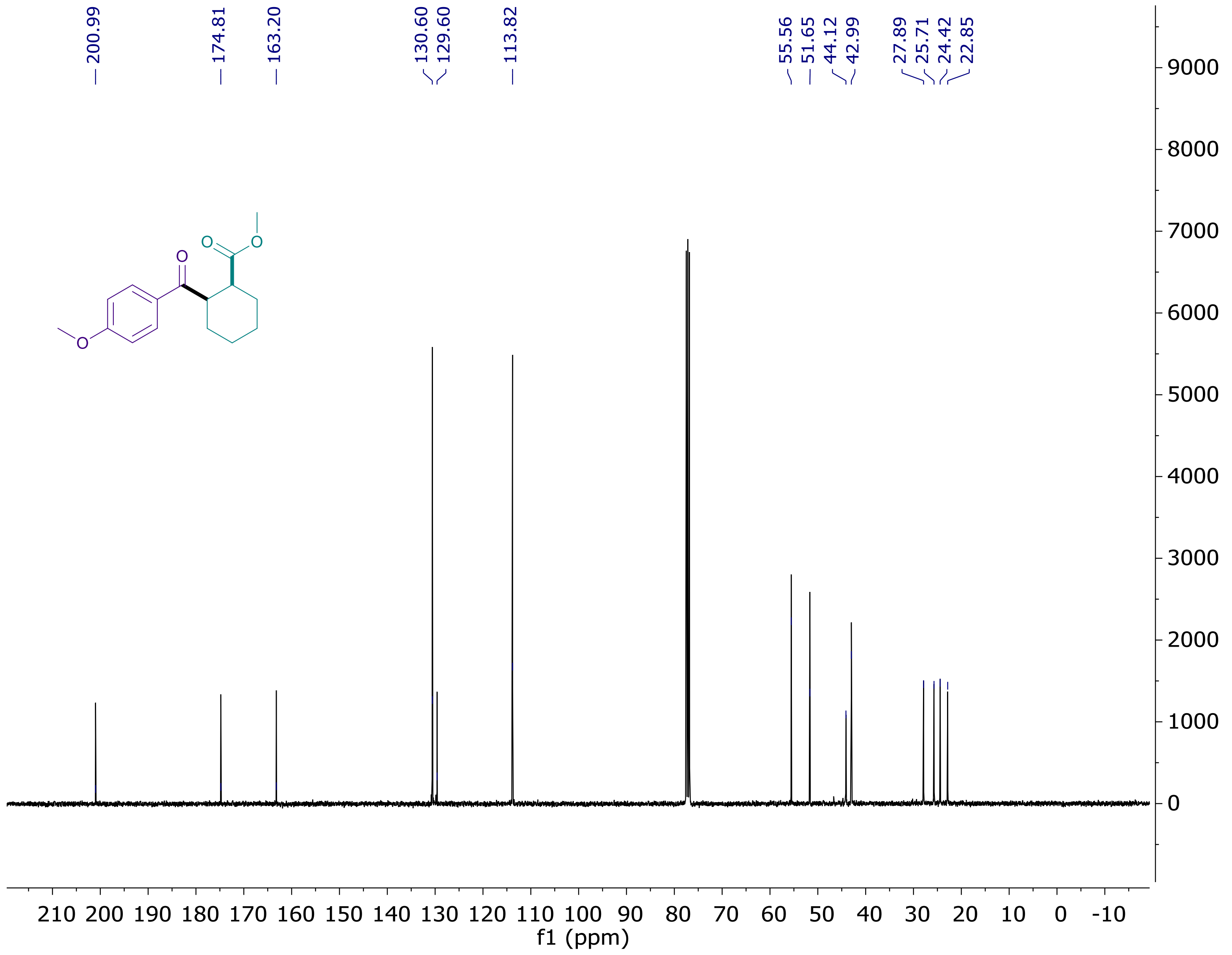

Compound 3r (minor diastereomer) – ^{1}H NMR (400 MHz, Chloroform-*d*):

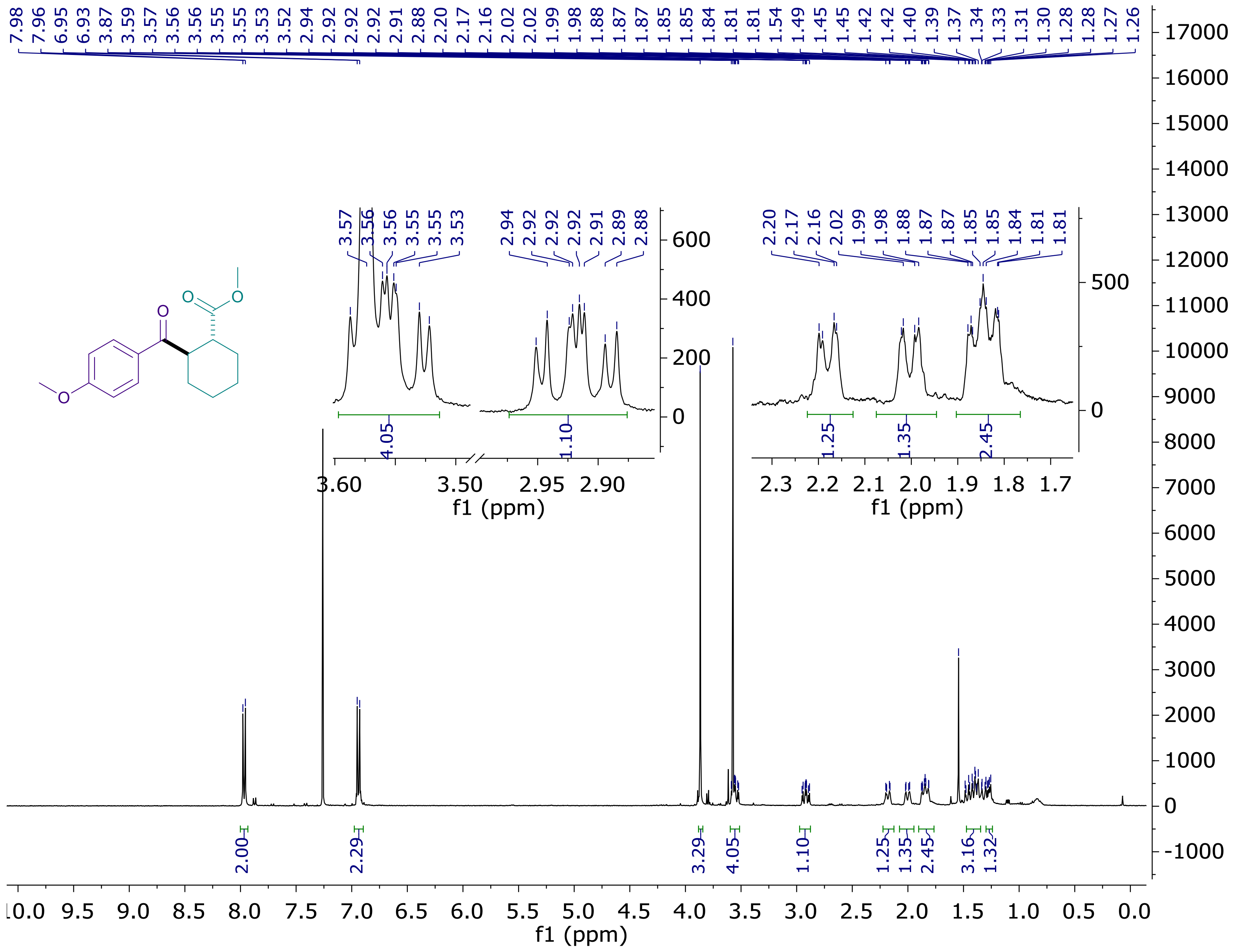

Compound 3r (minor distereomer) – $^{13}C\{^{1}H\}$ NMR (101 MHz, Chloroform-*d*):

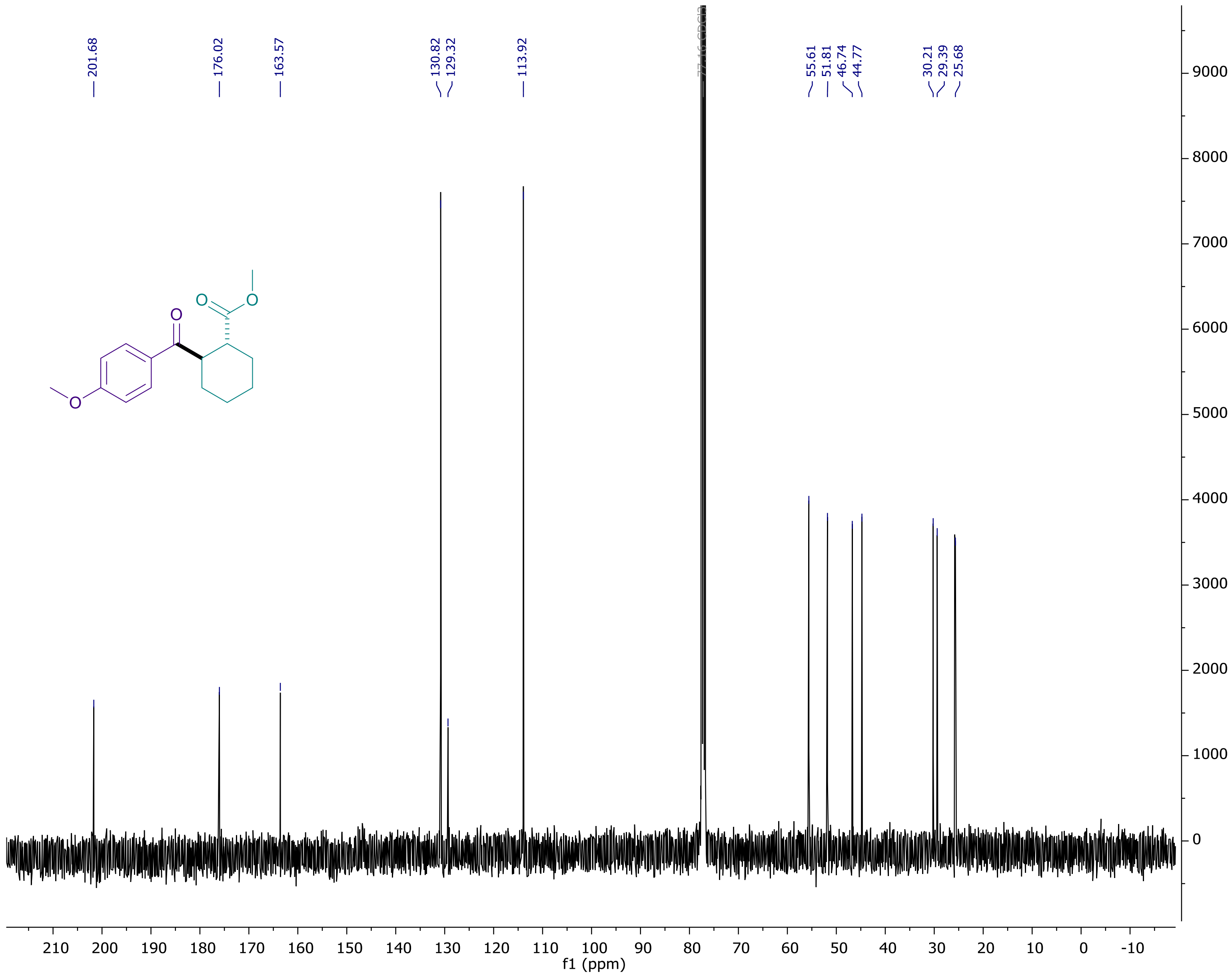

Compound 3s – ^{1}H NMR (400 MHz, Chloroform-*d*):

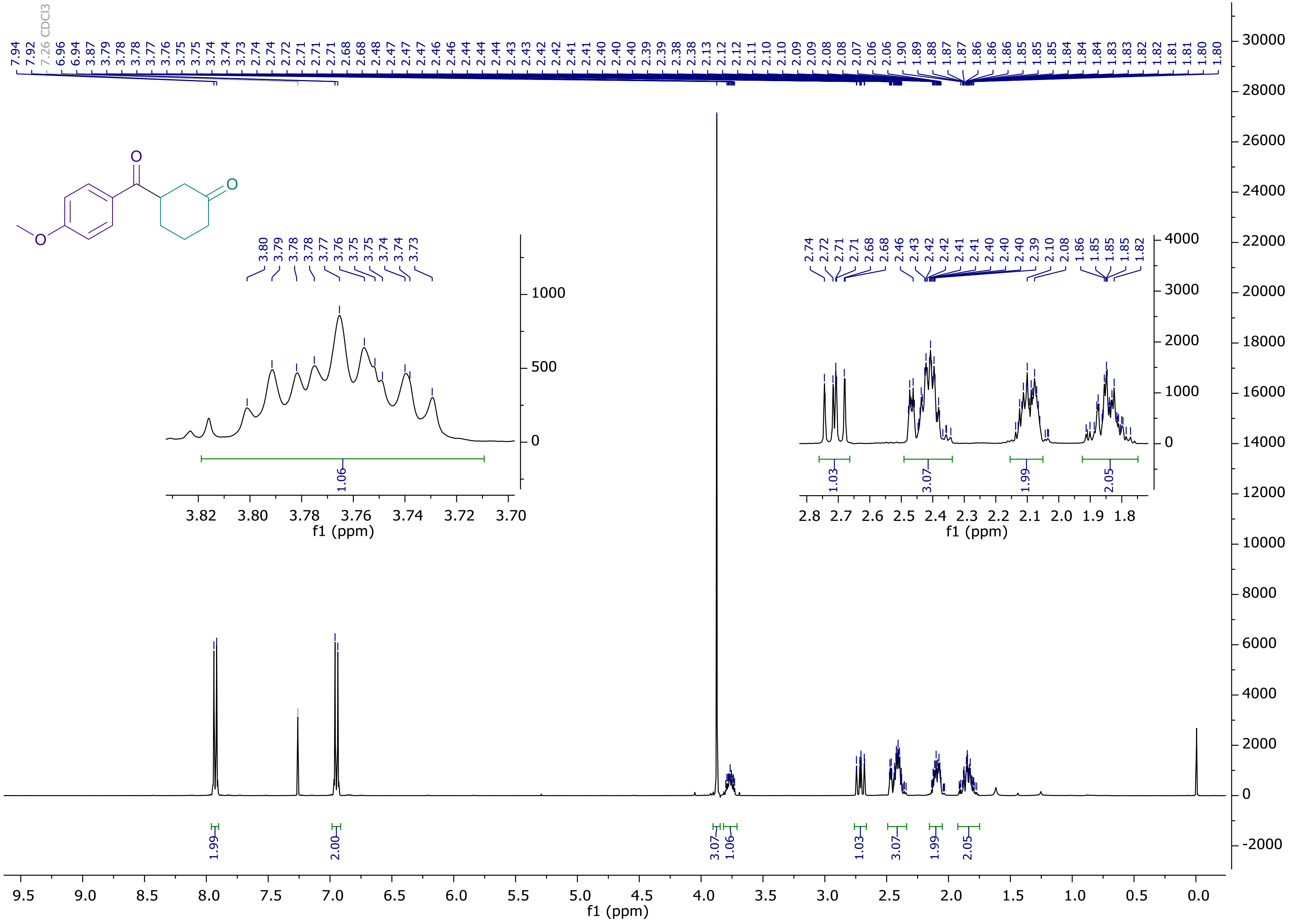

Compound 3s – $^{13}C\{^{1}H\}$ NMR (101 MHz, Chloroform-*d*):

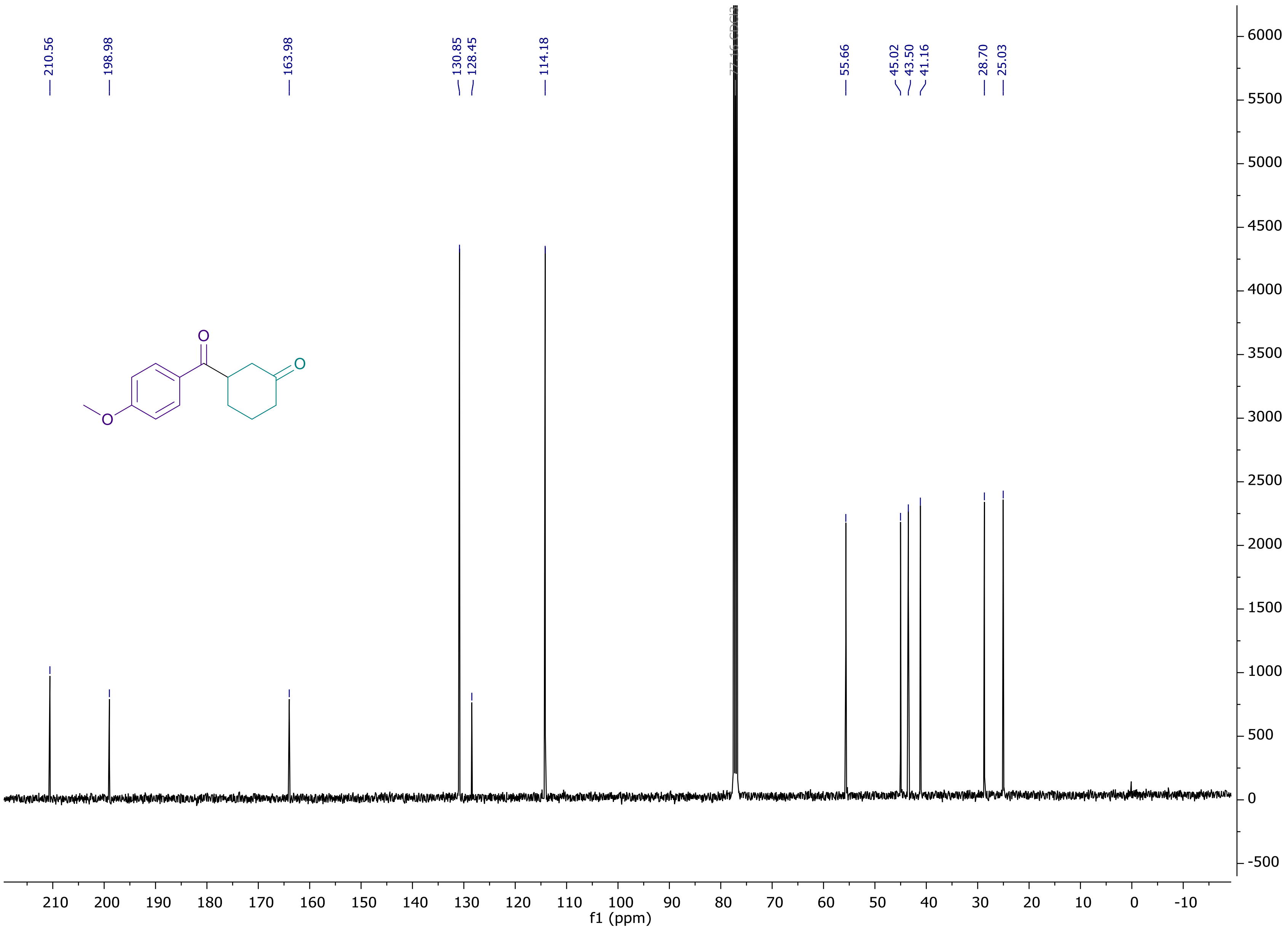

Compound 3t (major diastereomer) – ^{1}H NMR (400 MHz, Chloroform-*d*):

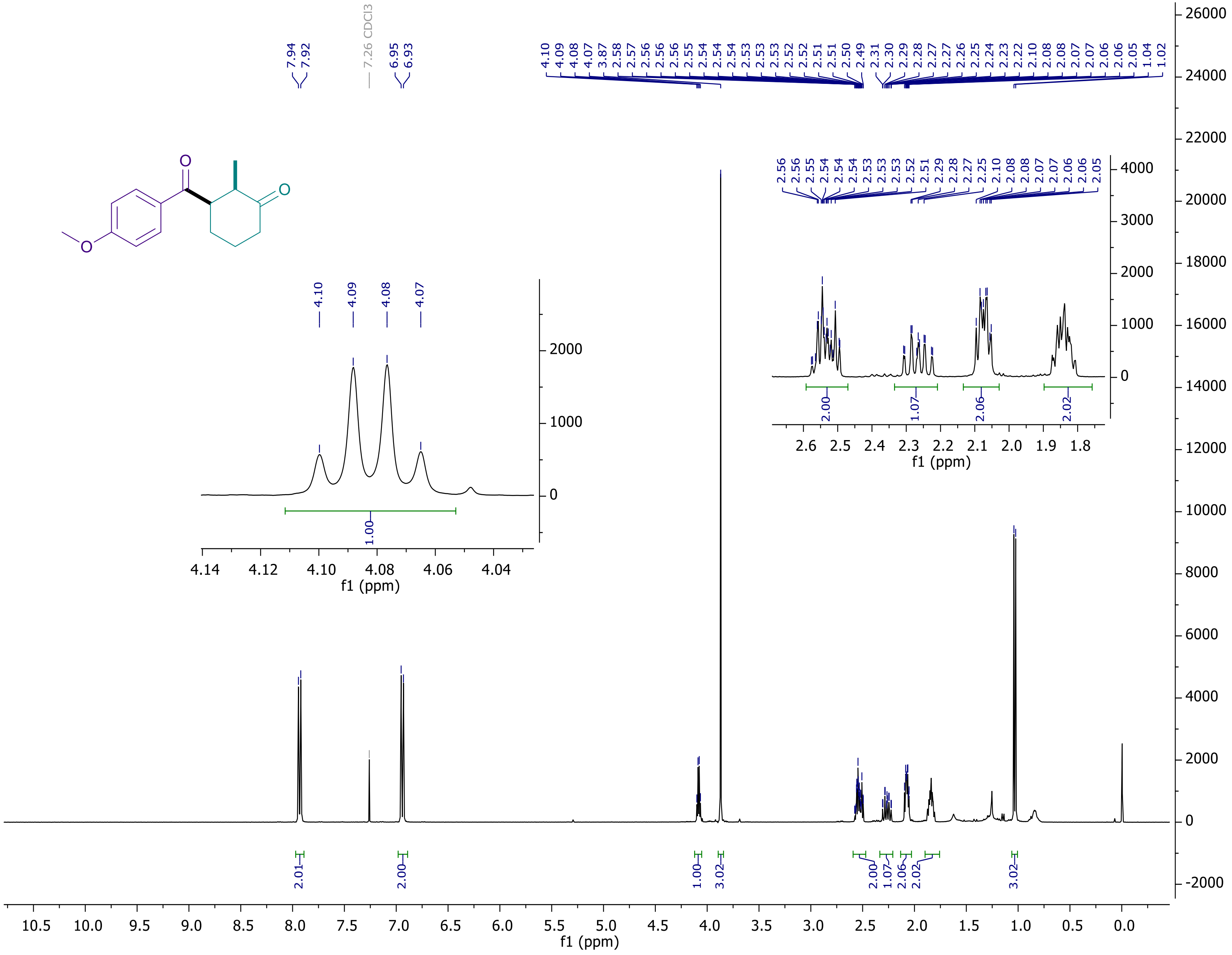

Compound 3t (major diastereomer) – $^{13}C\{^{1}H\}$ NMR (101 MHz, Chloroform-*d*):

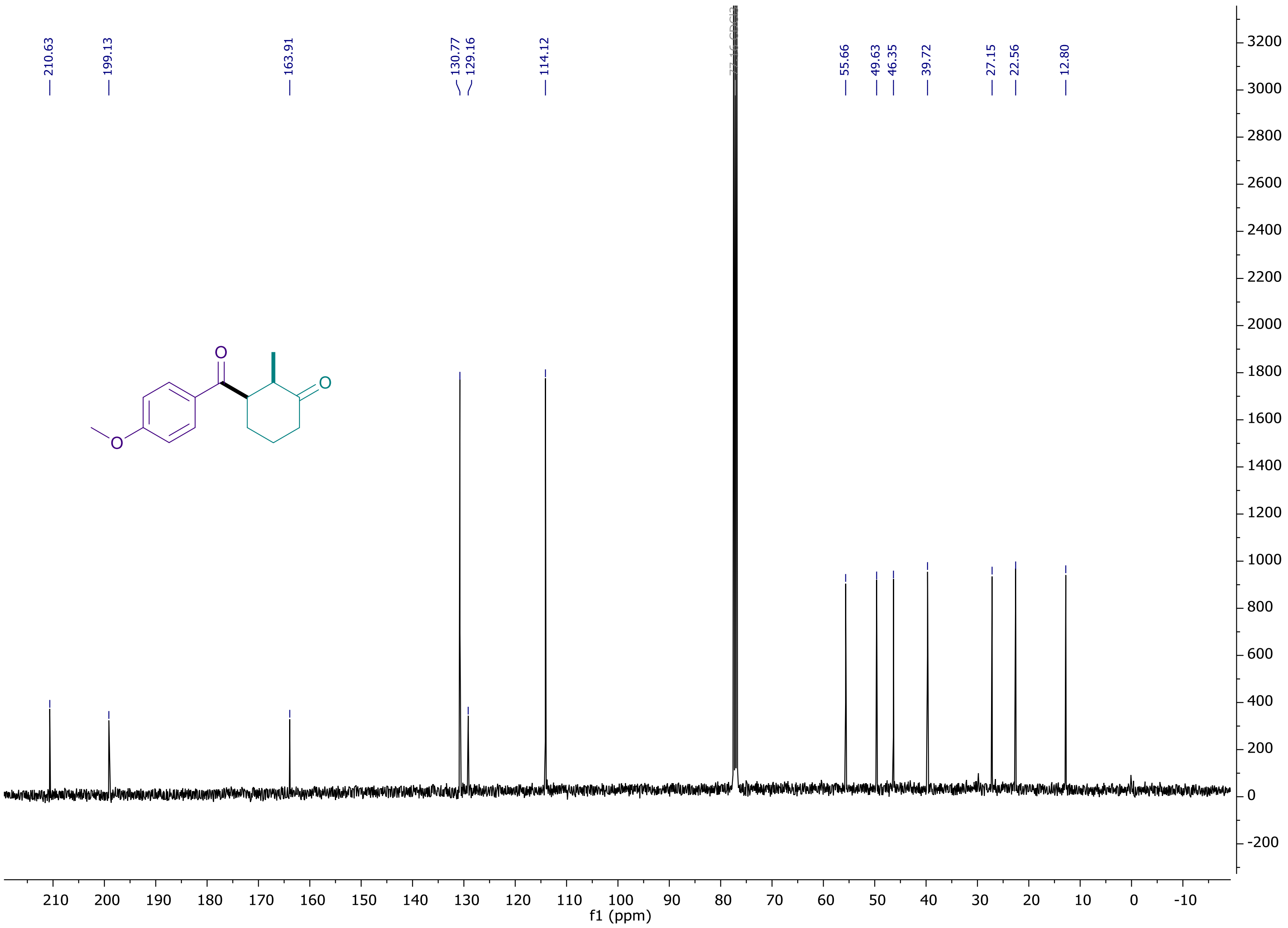

Compound 3t (minor diastereomer) – ^{1}H NMR (400 MHz, Chloroform-*d*):

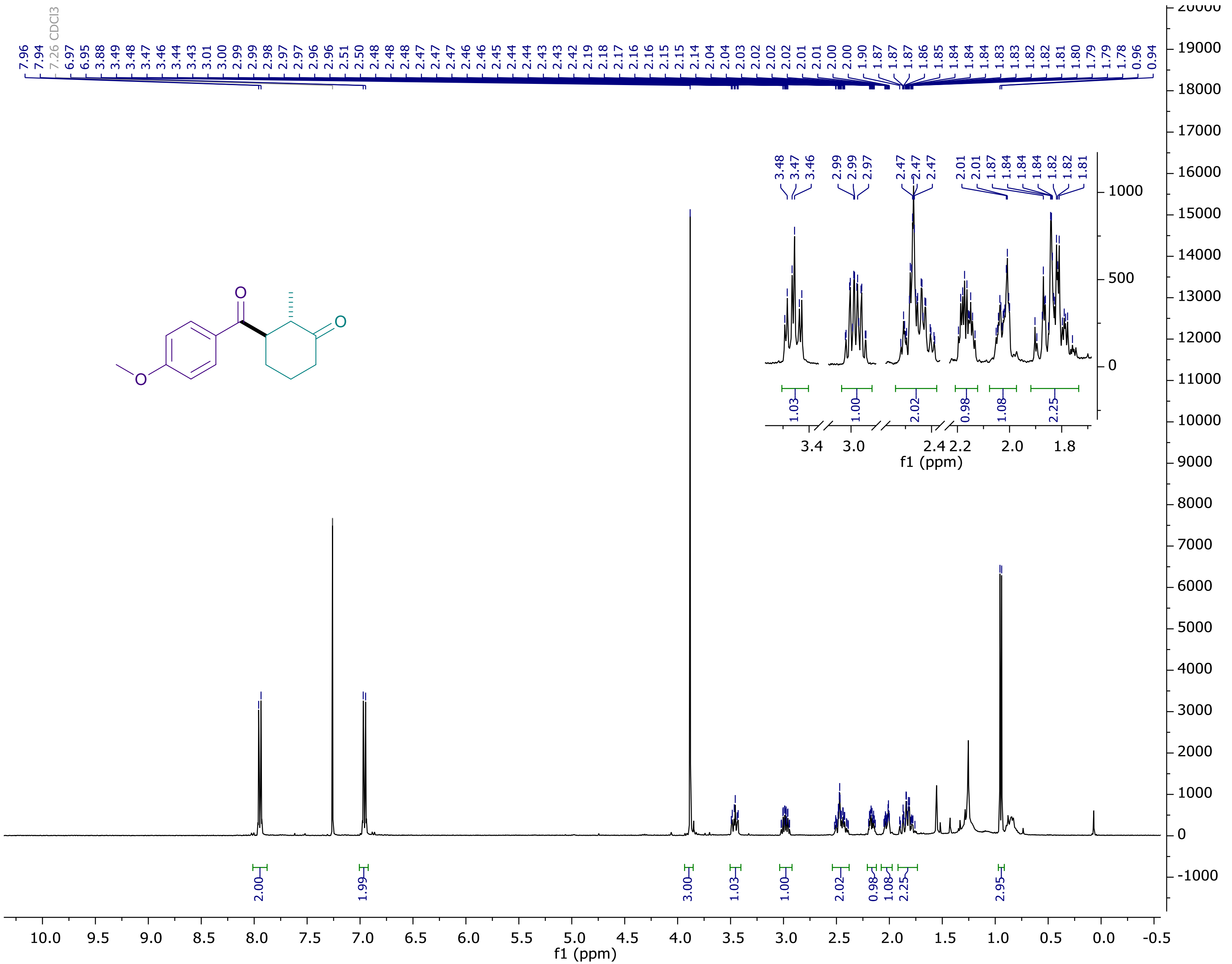

Compound 3t (minor diastereomer) – $^{13}C\{^{1}H\}$ NMR (101 MHz, Chloroform-*d*):

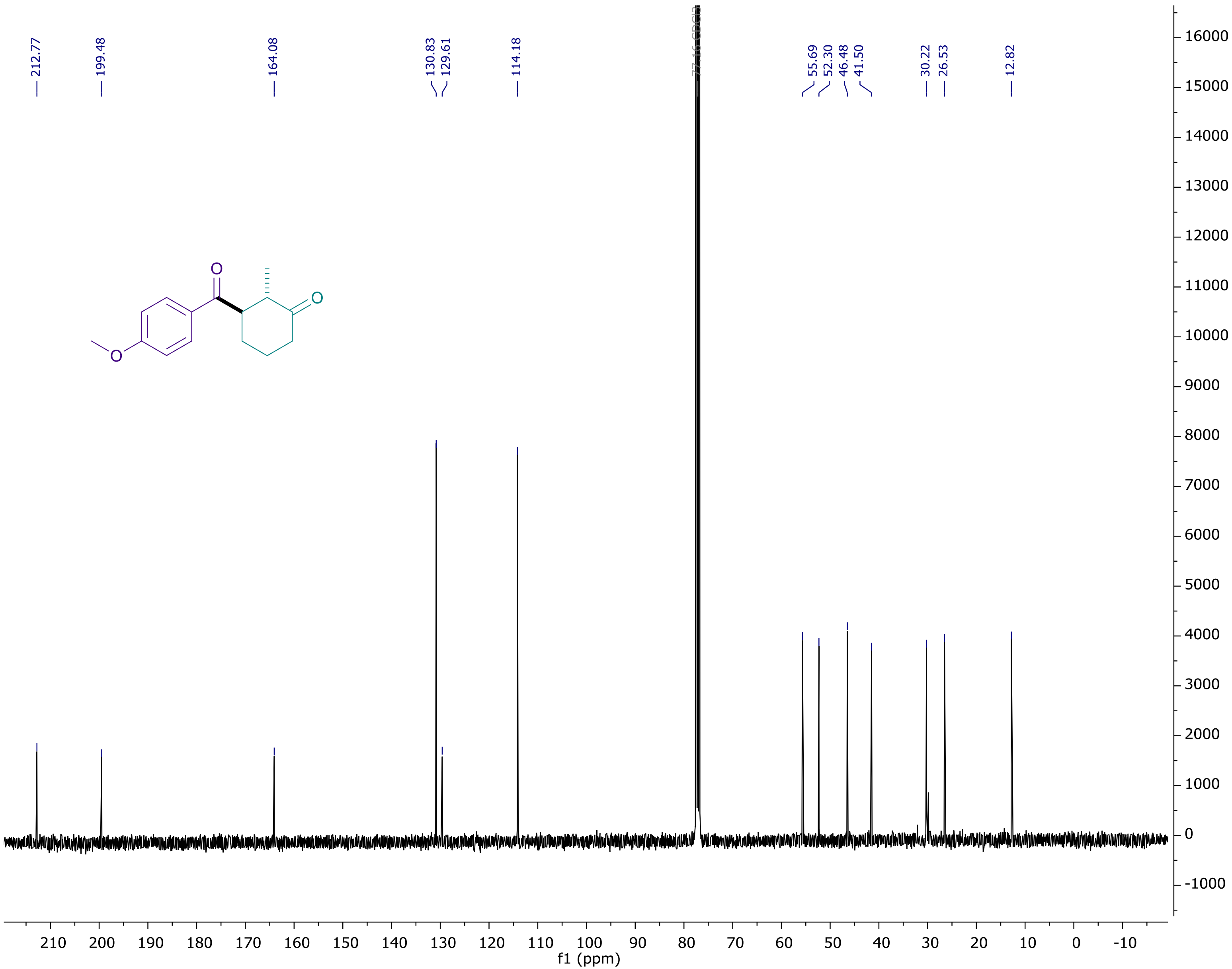

Compound 3u – ^{1}H NMR (400 MHz, Chloroform-*d*):

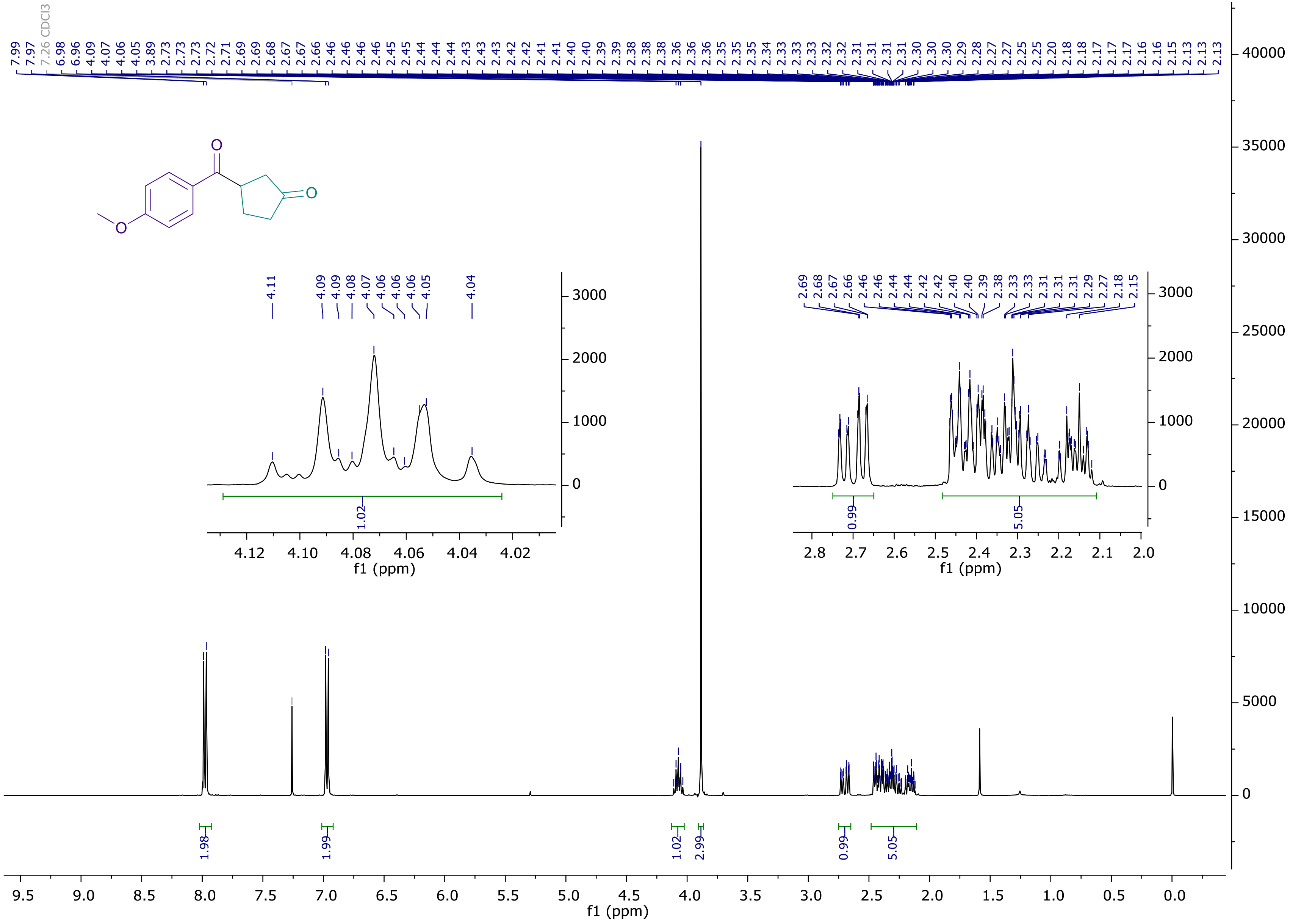

Compound 3u – $^{13}C\{^1H\}$ NMR (101 MHz, Chloroform-*d*):

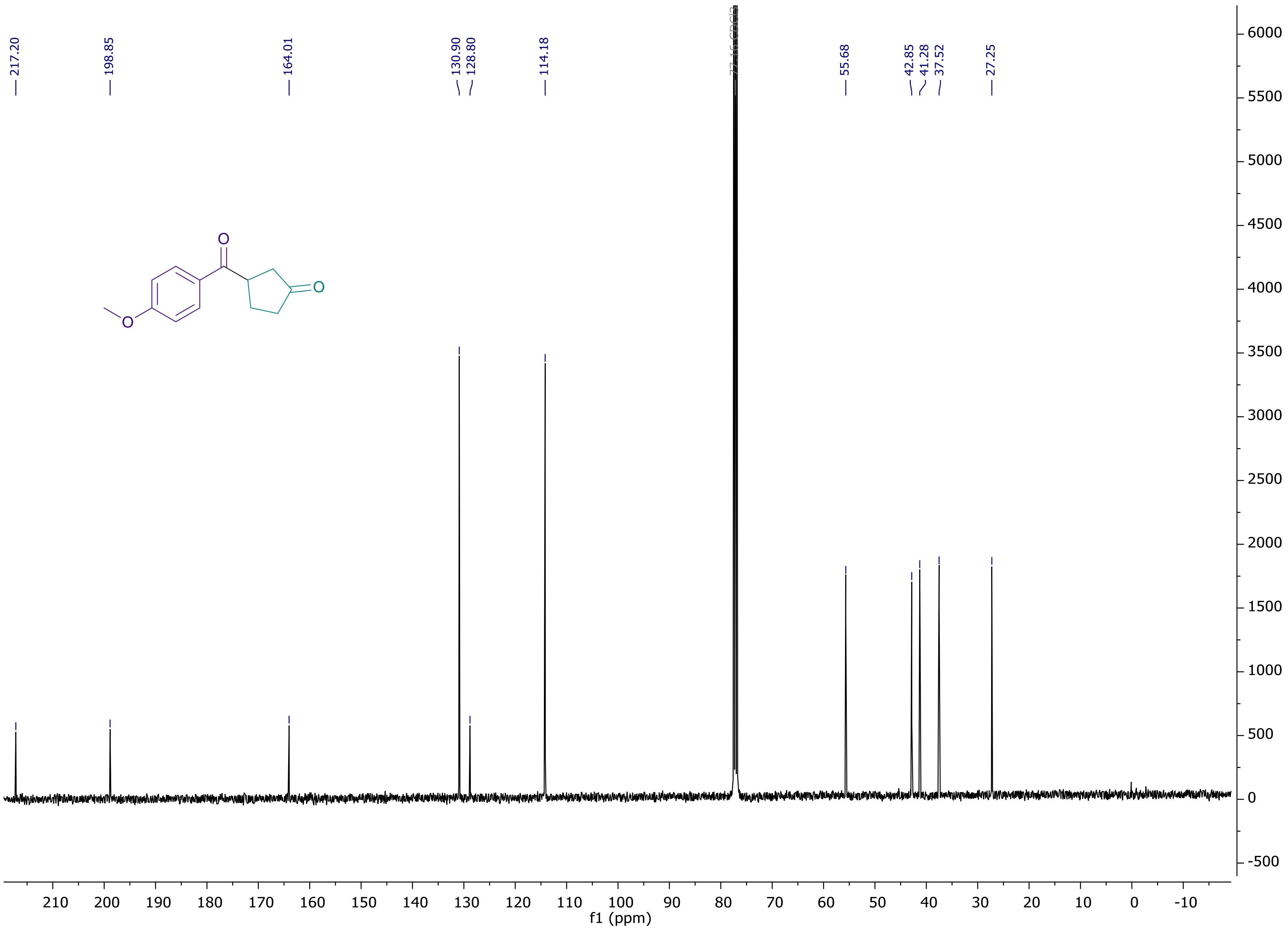

Compound 3v – ^{1}H NMR (400 MHz, Chloroform-*d*):

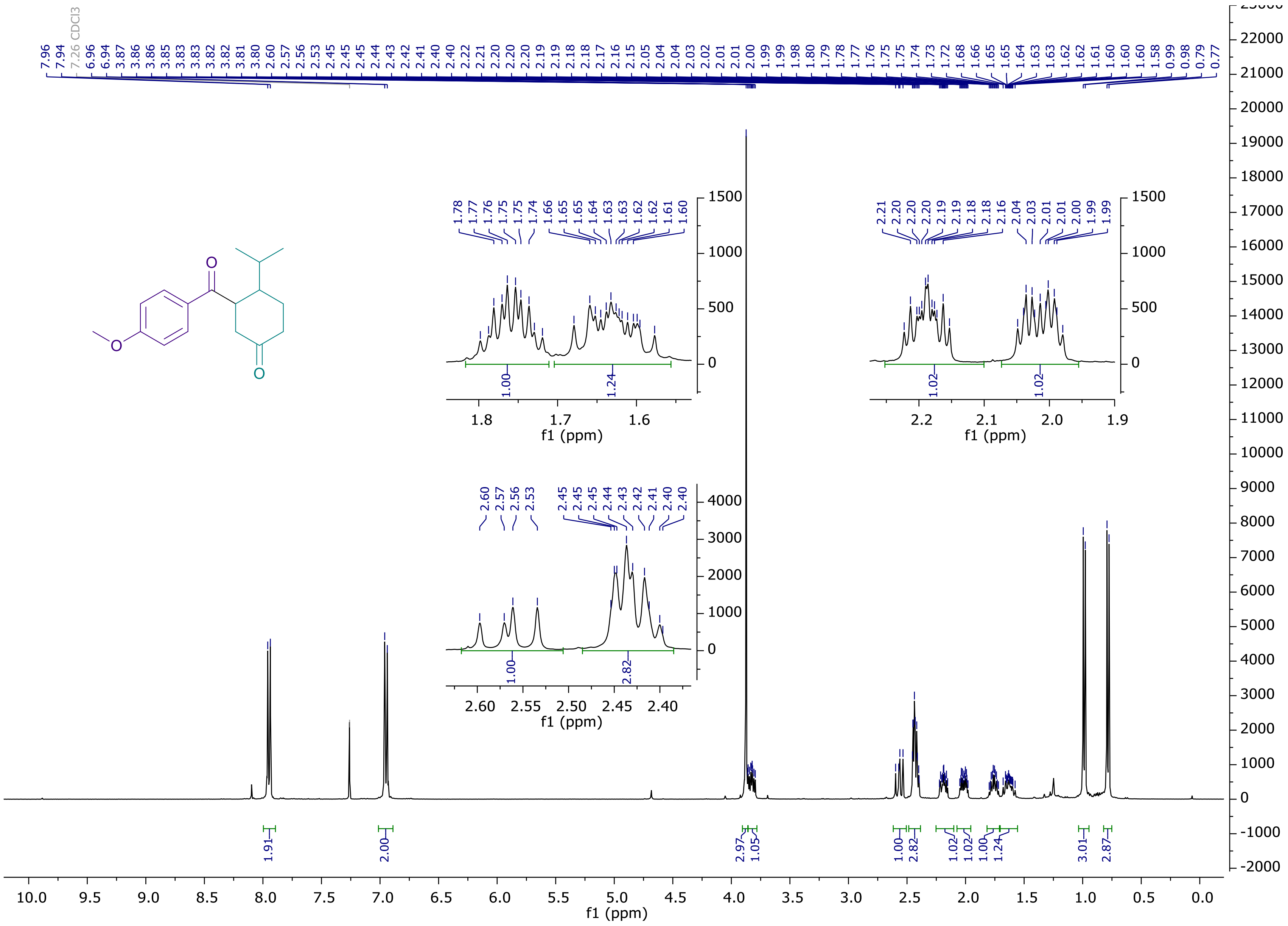

Compound 3v – $^{13}C\{^1H\}$ NMR (101 MHz, Chloroform-*d*):

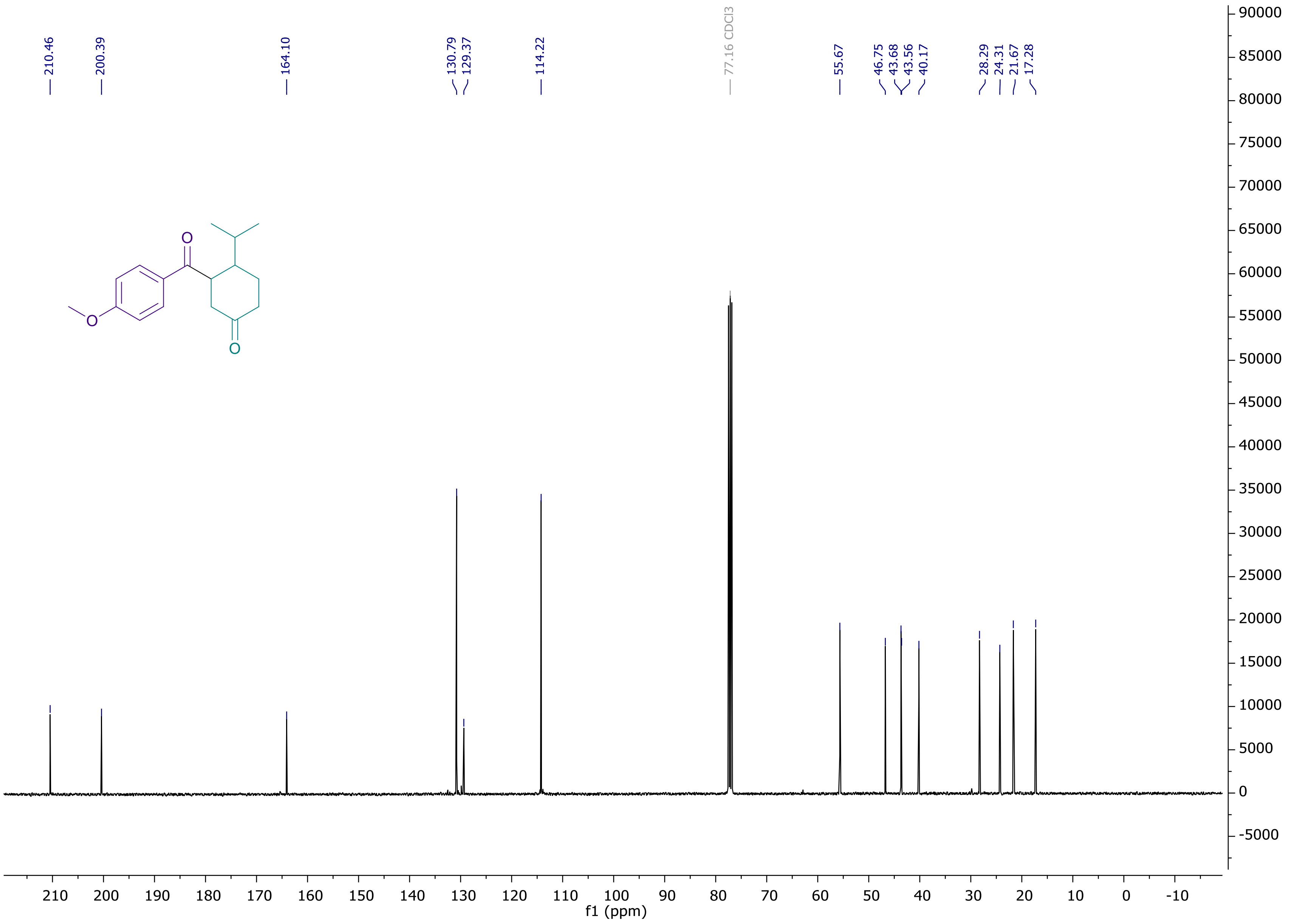

Compound 8 – ^{1}H NMR (400 MHz, Chloroform-d):

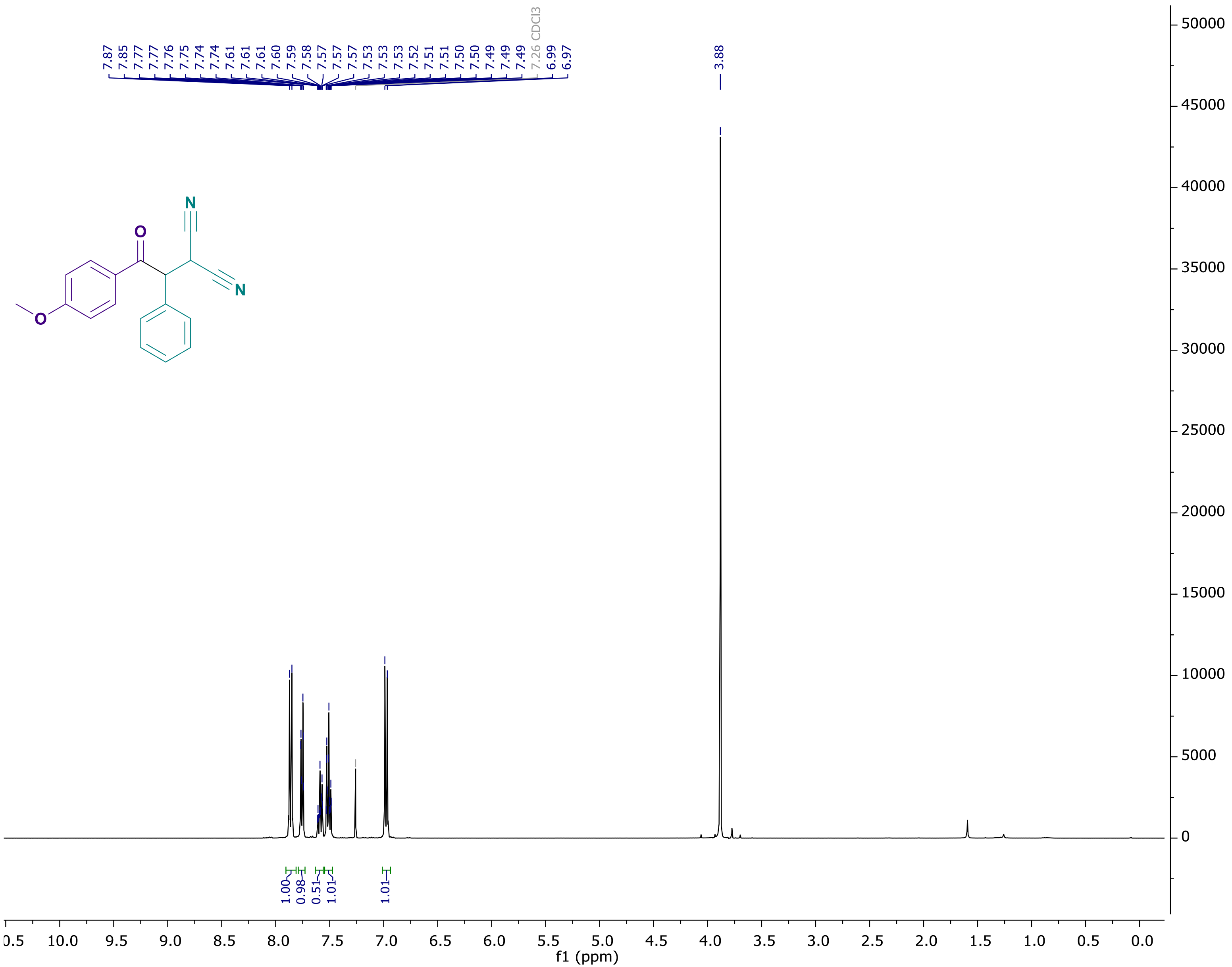

Compound 8 – $^{13}C\{^{1}H\}$ NMR (101 MHz, Chloroform-*d*):

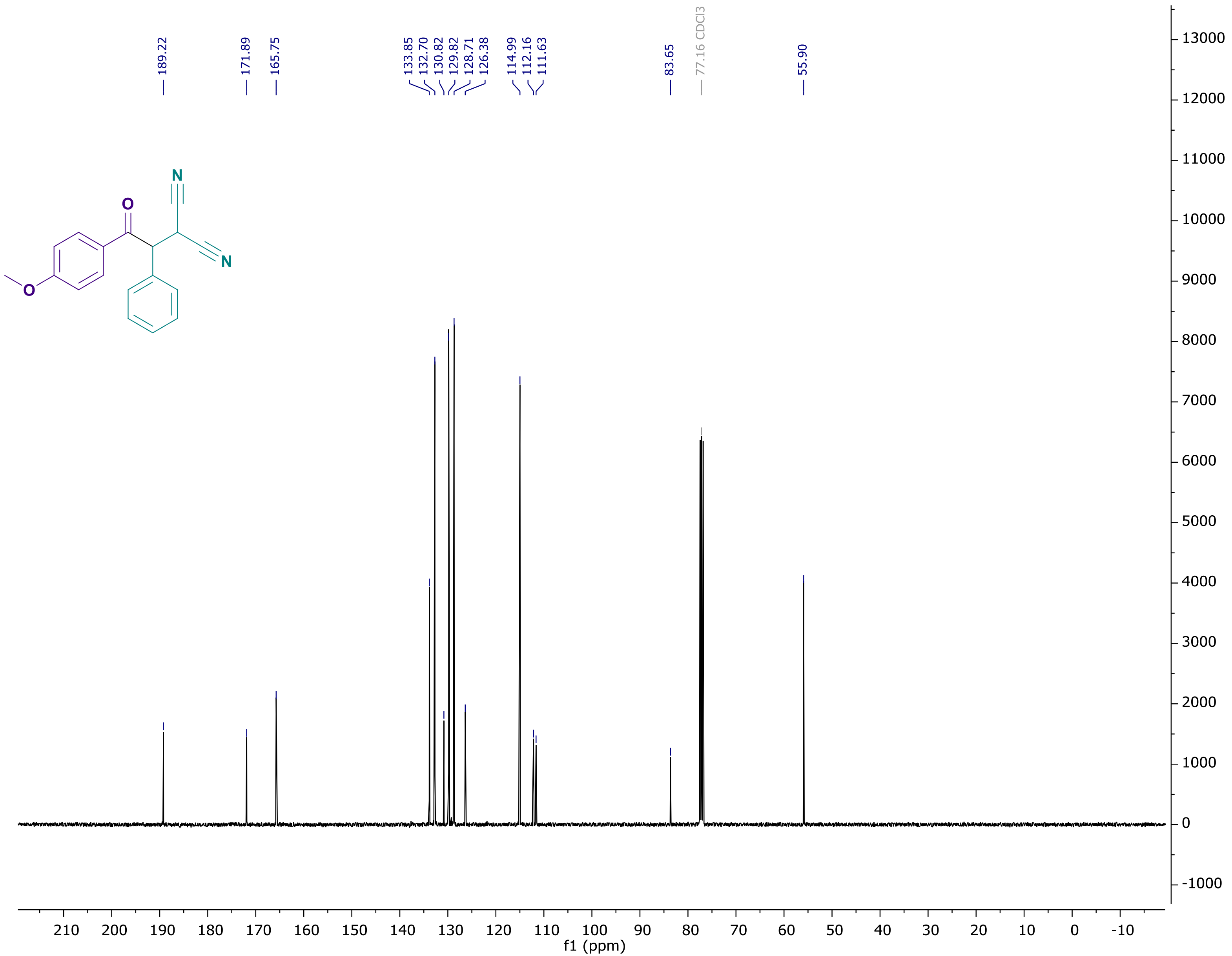

Compound 9 – ^{1}H NMR (400 MHz, Chloroform-*d*):

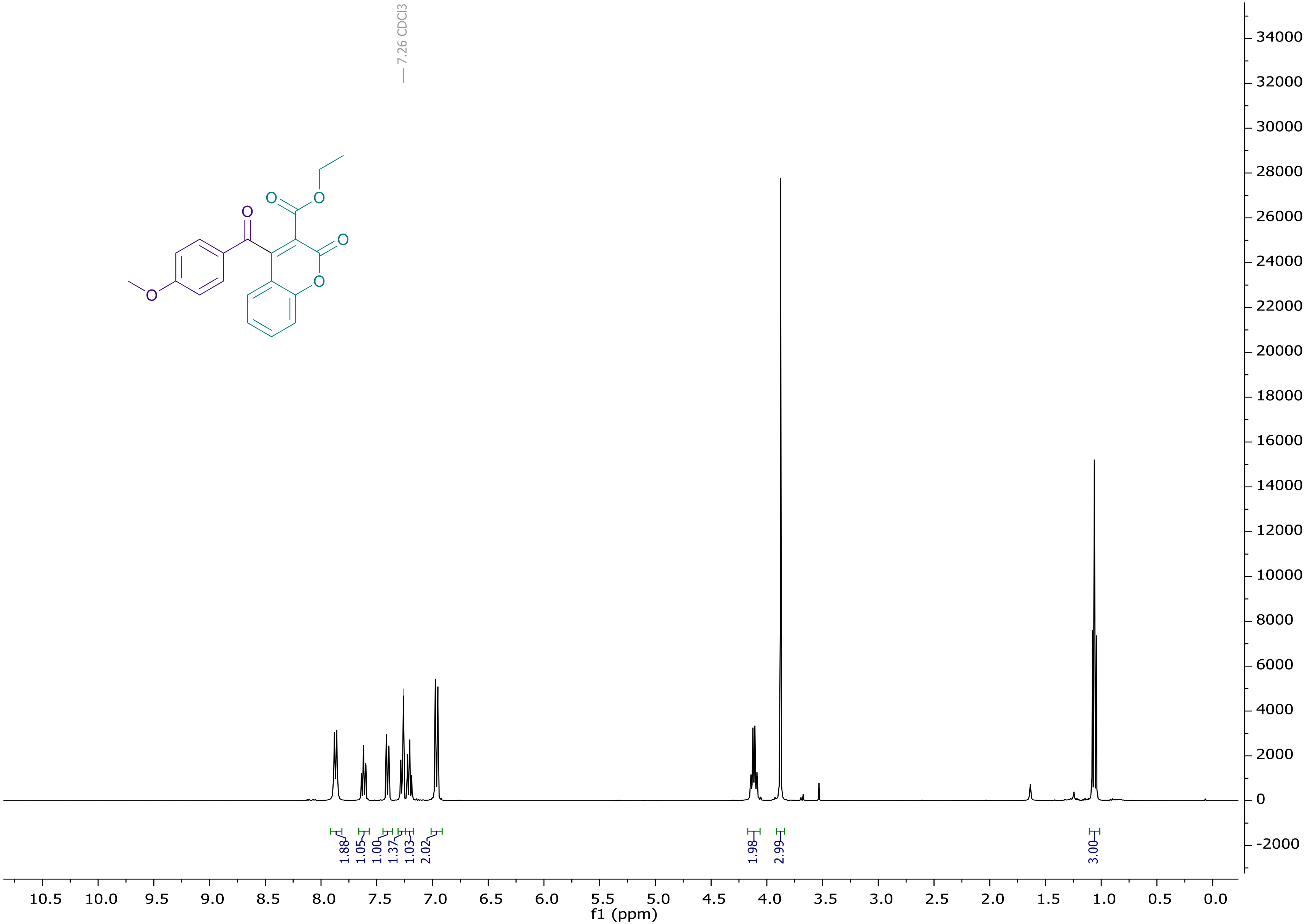

Compound 9 – $^{13}C\{^{1}H\}$ NMR (101 MHz, Chloroform-*d*):

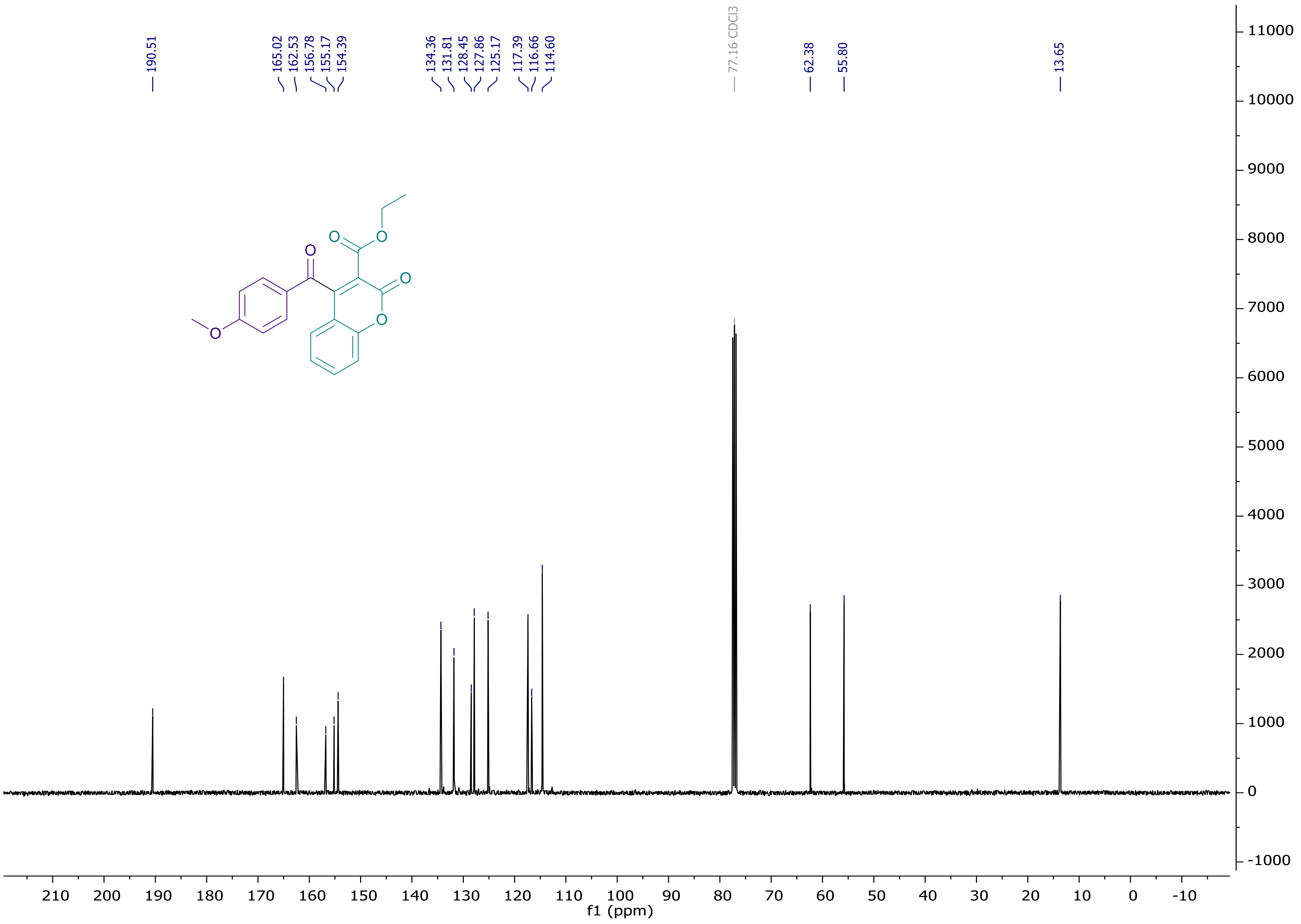

Compound 10 – ^{1}H NMR (400 MHz, Chloroform-*d*):

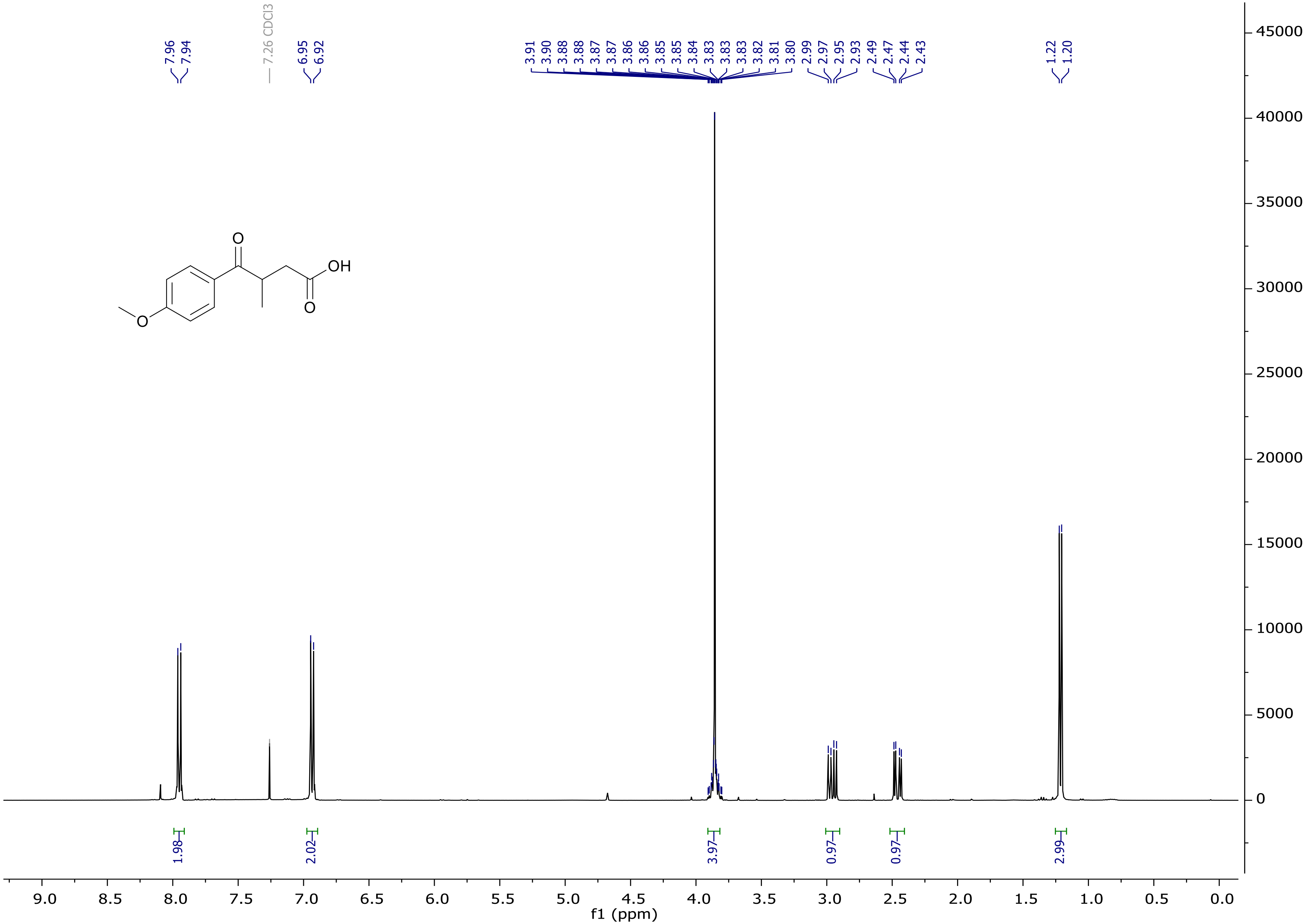

Compound 10 – $^{13}C\{^{1}H\}$ NMR (101 MHz, Chloroform-*d*):

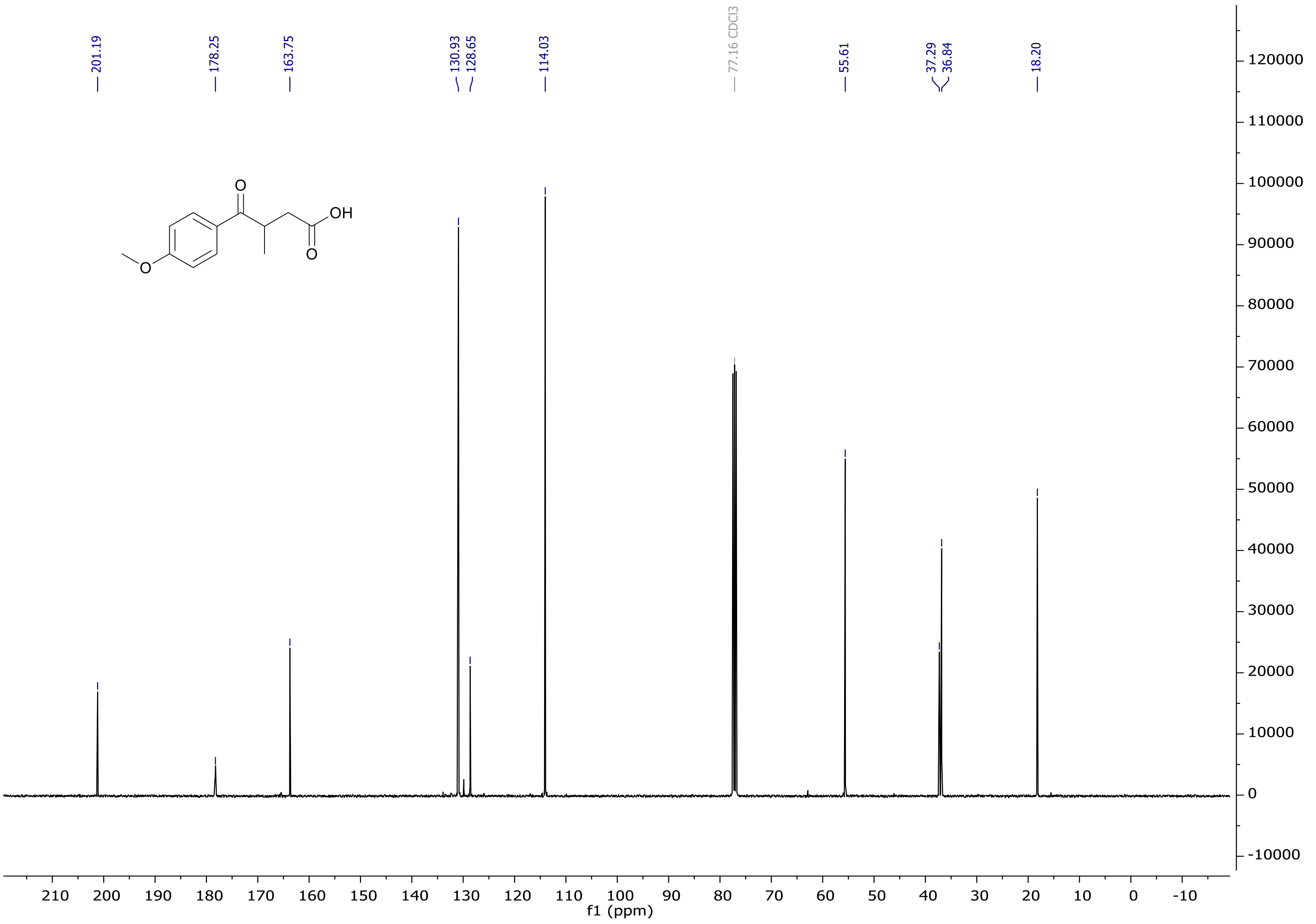

Compound 11 – ^{1}H NMR (400 MHz, Chloroform-*d*):

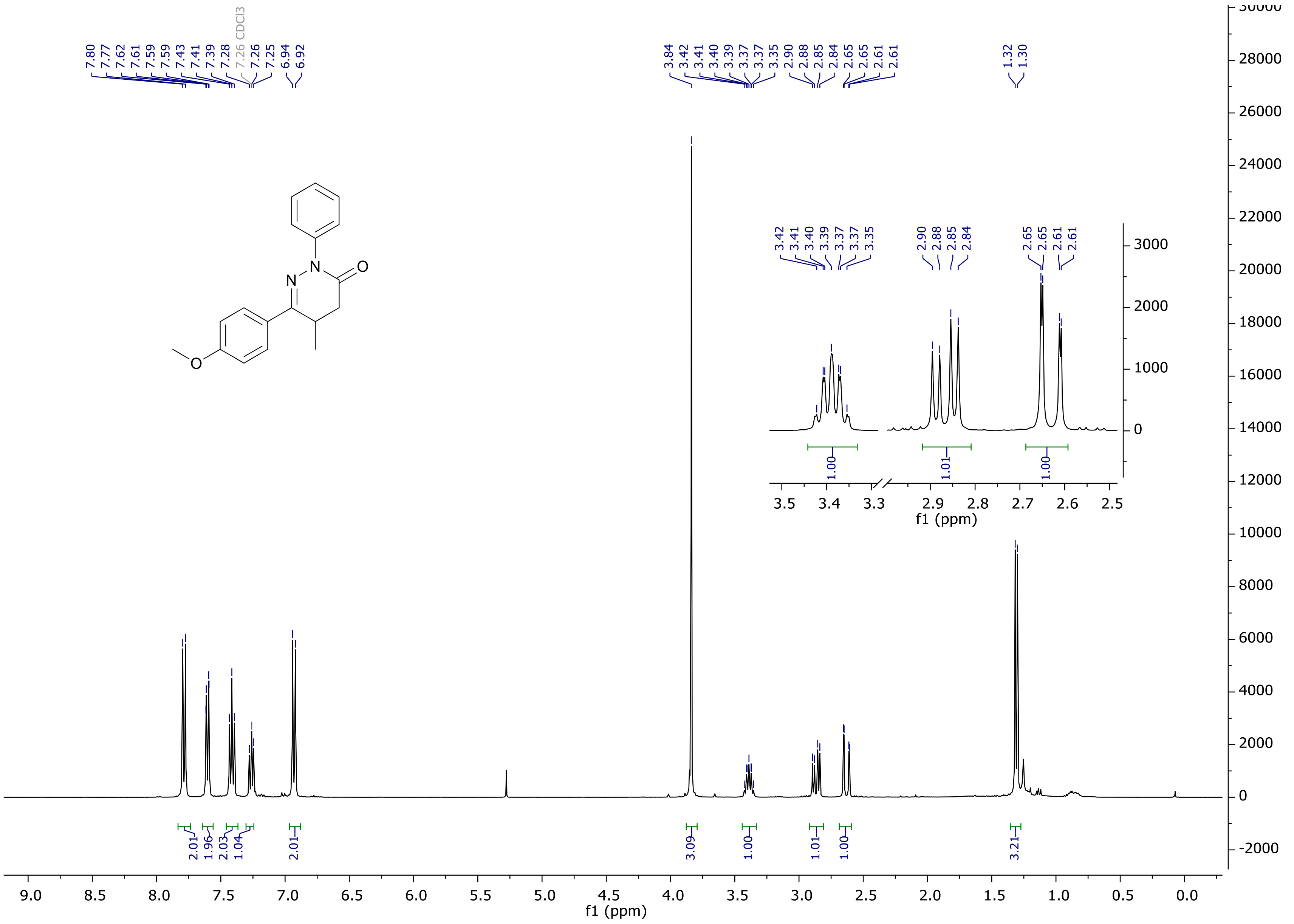

Compound 11 – $^{13}C\{^{1}H\}$ NMR (101 MHz, Chloroform-*d*):

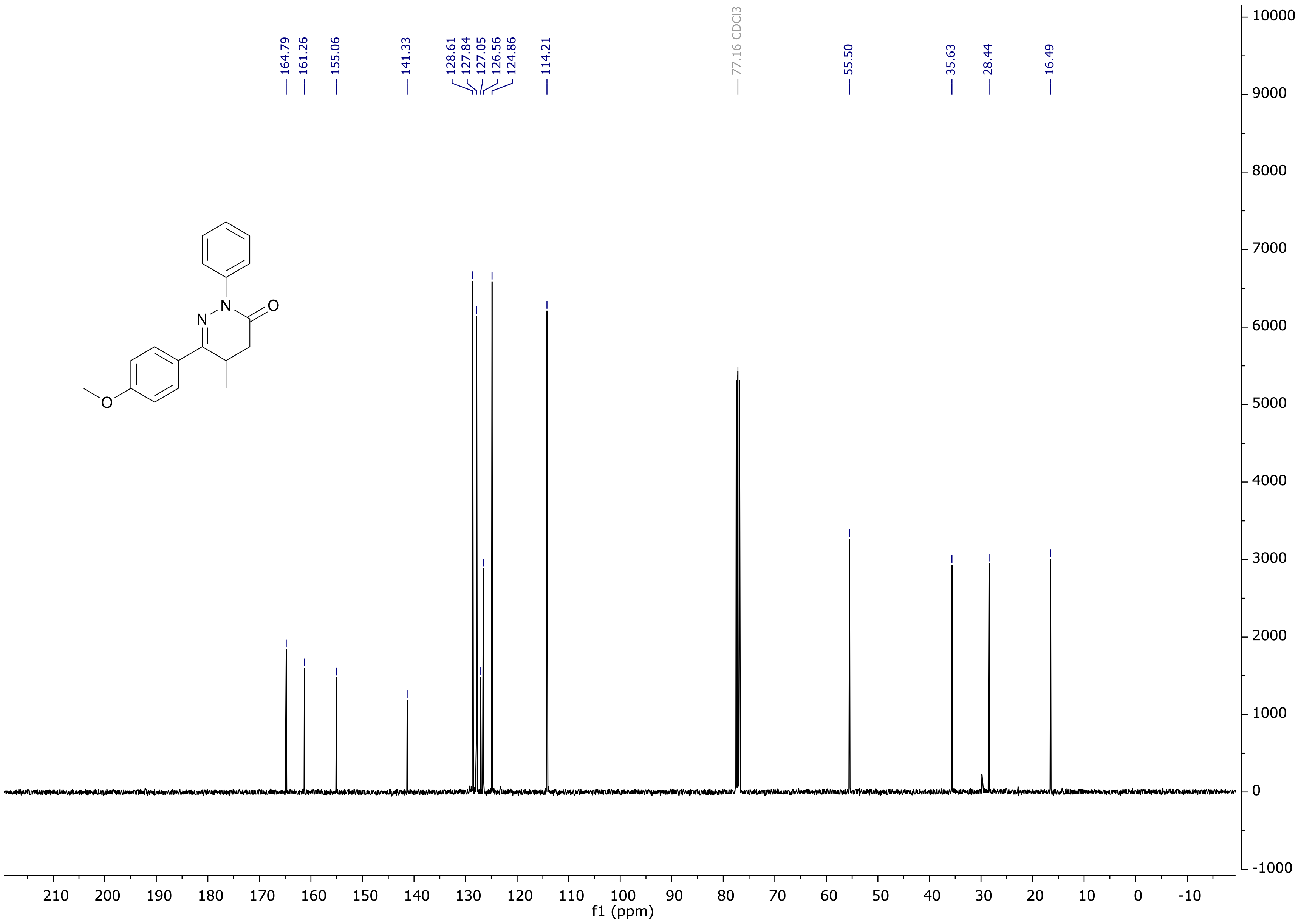

Supplement: Supplementary file 1 — jo4c02163_si_001.pdf [file jo4c02163_si_001.pdf]
